# Stapled helical *o*-OPE foldamers as new circularly polarized luminescence emitters based on carbophilic interactions with Ag(i)-sensitivity†
†We dedicate this work to Ettore Castiglioni who, with his high experience in the field of chiroptical spectroscopies, gave important contributions also to the development of CPL instrumentation.
‡
‡Electronic supplementary information (ESI) available: general experimental details, synthesis of all new substrates and complexes, ^1^H and ^13^C NMR spectra of the new compounds and the corresponding NMR titrations, photophysical and theoretical data. CCDC 1443131. For ESI and crystallographic data in CIF or other electronic format see DOI: 10.1039/c6sc01808d


**DOI:** 10.1039/c6sc01808d

**Published:** 2016-05-17

**Authors:** Sara P. Morcillo, Delia Miguel, Luis Álvarez de Cienfuegos, José Justicia, Sergio Abbate, Ettore Castiglioni, Christophe Bour, María Ribagorda, Diego J. Cárdenas, José Manuel Paredes, Luis Crovetto, Duane Choquesillo-Lazarte, Antonio J. Mota, M. Carmen Carreño, Giovanna Longhi, Juan M. Cuerva

**Affiliations:** a Institut de Chimie Moléculaire et des Matériaux d'Orsay , CNRS UMR 8182 , Univ. Paris-Sud Université Paris-Saclay bâtiment 420 , 91405 Orsay cedex , France; b Department of Organic Chemistry , University of Granada (UGR) , C. U. Fuentenueva , 18071 Granada , Spain . Email: jmcuerva@ugr.es ; Email: dmalvarez@ugr.es; c Dipartimento di Medicina Molecolare e Traslazionale , Università di Brescia , Viale Europa 11 , 25123 Brescia , Italy . Email: giovanna.longhi@unibs.it; d Departamento de Química Orgánica , Universidad Autónoma de Madrid , c/Francisco Tomás y Valiente n° 7, Cantoblanco , 28049 Madrid , Spain; e Department of Physical Chemistry , Faculty of Pharmacy , UGR , Cartuja Campus , 18071 Granada , Spain; f Laboratorio de Estudios Cristalográficos , Instituto Andaluz de Ciencias de la Tierra (CSIC-UGR) , 18100 Armilla , Granada , Spain; g Department of Inorganic Chemistry , UGR , C. U. Fuentenueva , 18071 Granada , Spain

## Abstract

*ortho*-Oligo(phenylene)ethynylenes (*o*-OPEs) stapled with enantiopure 2,3-dihydroxybutane diethers have highly intense circular dichroism (CD) spectra and excellent circular polarized luminescence (CPL) responses (*g*_lum_ values up to 1.1 × 10^–2^), which are consistent with homochiral helically folded structures. In the presence of Ag(i), a change in the CPL emission is observed, representing the first example of CPL active small organic molecular emitters, which can be modulated by carbophilic interactions in a reversible manner.

## Abstract 2

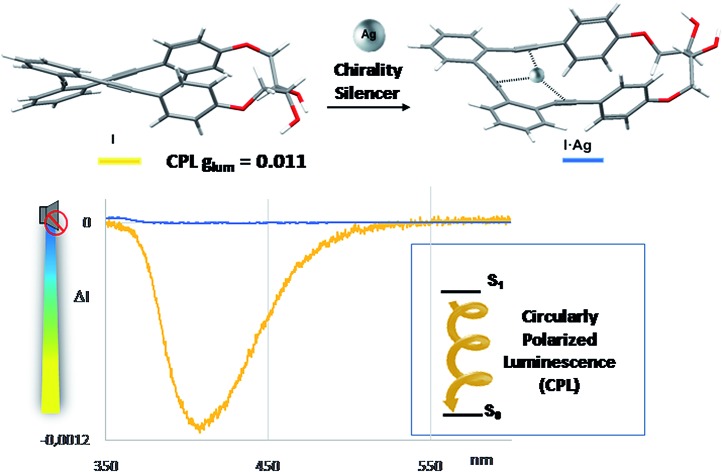
Chiral stapled *o*-OPEs show excellent circular polarized luminescence responses (*g*_lum_ of 1.1 × 10^–2^) which can be modulated by carbophilic interactions.

## Introduction

Homochiral organic structures capable of producing efficient CPL responses are of huge interest due to their promising applications as new luminescent materials such as optical devices and biosensors.^1^ Different enantioenriched molecules, such as helicenes,^2^,^3^ lanthanide complexes,^4^ and helical polymers,^5^ have been reported to exhibit high CPL in solution, as aggregates, or in the solid state, due to their self-organized structures. The few reported CPL active organic molecules up to now have showed small dissymmetry factors (*g*_lum_) in solution, with very few exceptions such as super-organized cholesteric crystals.^6^ Unfortunately, supramolecular organization has a negative influence on the emission efficiency. The synthetic accessibility and great versatility of simple chiral organic molecules make them attractive circular polarized luminescence probe candidates. New and efficient applications require the development of simple structures able to maintain chiral environments in the excited state while maintaining a reasonable fluorescence quantum yield (*Φ*). Within this context, some [*n*]-helicene-type compounds have shown high *g*_lum_ values (10^–2^ to 10^–3^) although their quantum yields are usually low.^7^ A possible solution to improve the intrinsic CPL characteristics of [*n*]-helicenes could be the construction of rigid helical structures with large magnetic transition dipole moments (rotational strengths) in which the self-quenching process is decreased. Control of CPL emission by external factors in simple molecular emitters is even less common, although it can be viewed as a potential probe for them. To the best of our knowledge only anions (halides, acetate and l-phenylalanine anions), ammonium salts, light, Zn(ii) cation and acids/bases, have been used as external modulators of CPL emission.^8^

**Fig. 1:**
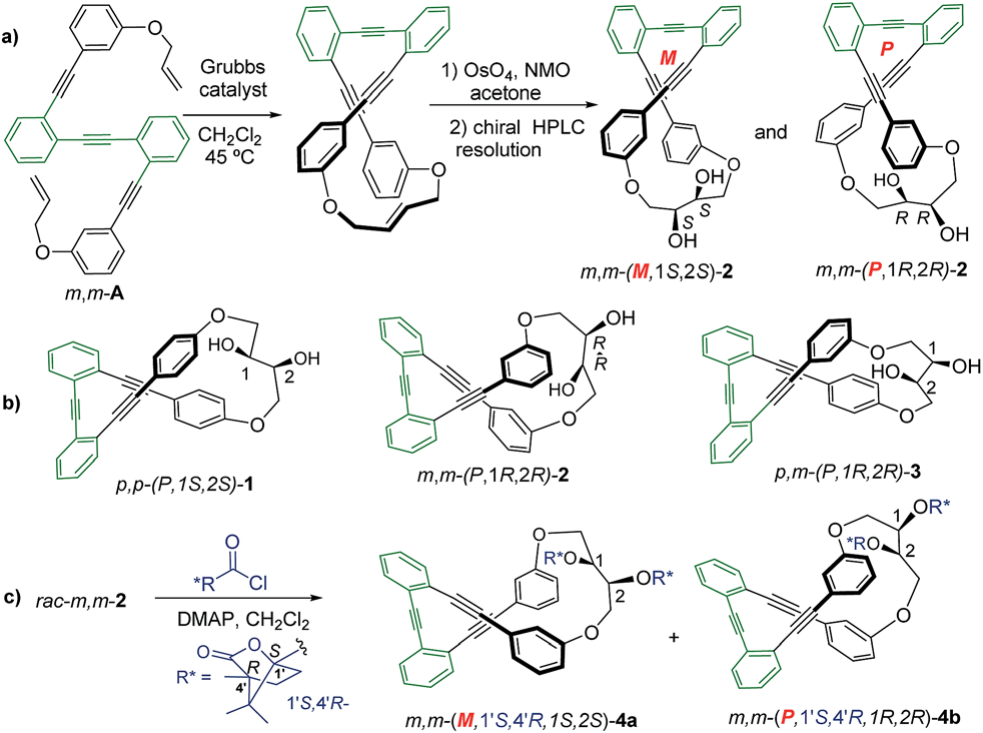
Representative synthetic route (a) to enantiopure diol *m*,*m*-(*M*,1*S*,2*S*)-**2** and *m*,*m*-(*P*,1*R*,2*R*) **2**. (b) Structure of *p*,*p*-**1**, *m*,*m*-**2** and *p*,*m*-**3** enantiomers and (c) synthesis of diastereomeric (1′*S*,4′*R*)-camphanoyl esters **4a** and **4b**.

We recently reported that fluorescent *ortho*-oligo(phenylene)ethynylenes (*o*-OPEs) can be stapled into helical conformations, even in a chiral way.^9^ This structural restriction provided new chiroptical properties to the system. Moreover, the inner cavity was able to selectively bind the Ag(i) cation, giving rise to a new class of Ag(i)-based metalofoldamers.^10^ As an extension of this previous work, we now report a new easy-to-tune class of molecular CPL active compounds **1–4** (Fig. 1) showing excellent *g*_lum_ (up to 1.1 × 10^–2^) values. The structures, based on *o*-OPEs, are easily accessible and show, as a significant feature, a CPL emission which is modulated in the presence of Ag(i) cations. Modulation of the chiroptical emission in solution using a carbophilic metal is unprecedented in the literature. The only related example, described by Crassous,^8^ is based on heliceneterpyridine:Zn(ii) interactions. Remarkably, silver could be later removed by the simple addition of MeCN, meaning that the CPL response of the *o*-OPEs recovered. This result provides a unique class of CPL-active silver sensitive molecular switches.

## Results and discussion

Racemic *para*,*para*-(*p*,*p*-), *meta*,*meta*-(*m*,*m*-) and *para*,*meta*-(*p*,*m*-) substituted *o*-OPEs **1–3**, containing a 2,3-butanediol fragment, were prepared starting from suitably positioned (*p*- or *m*-) allyl aryl ethers **A** (Fig. 1) using a Ru-catalyzed alkene metathesis and dihydroxylation of the resulting butene fragments (Fig. 1a, representative synthesis of *m*,*m*-**2**). Preparative chiral HPLC resolution (see ESI‡) allowed the isolation of both enantiomers of *p*,*p*-**1**, *m*,*m*-**2** and *p*,*m*-**3** (Fig. 1b, only one enantiomer is represented). In addition to the stereogenic diols present on each enantiomer, a new element of chirality (helicity) has been stereoselectively produced by the stapling process, thus generating interesting chiroptical properties. Chemical resolution of the diols *rac-m*,*m*-**2** was achieved through the formation and separation of the corresponding (1′*S*,4′*R*)-camphanoyl esters **4a** and **4b** (Fig. 1c), bearing different helicities in each case.

**Fig. 2:**
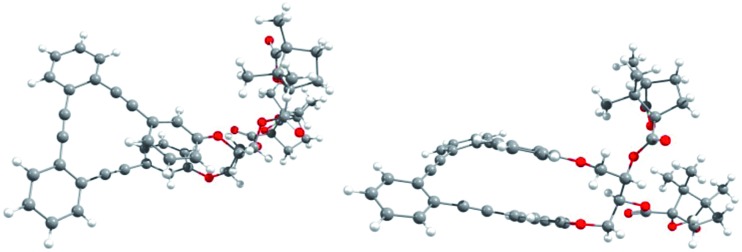
X-Ray structure of (1′*S*,4′*R*,*M*,1*S*,2*S*)-**4a**: top view (left) and side view (right).

The absolute (1′*S*,4′*R*,*M*,1*S*,2*S*) configuration of *m*,*m*-**4a** was established unequivocally using X-ray diffraction, taking into account the known (1′*S*,4′*R*) configuration of the camphanoyl moieties (Fig. 2).

**Fig. 3:**
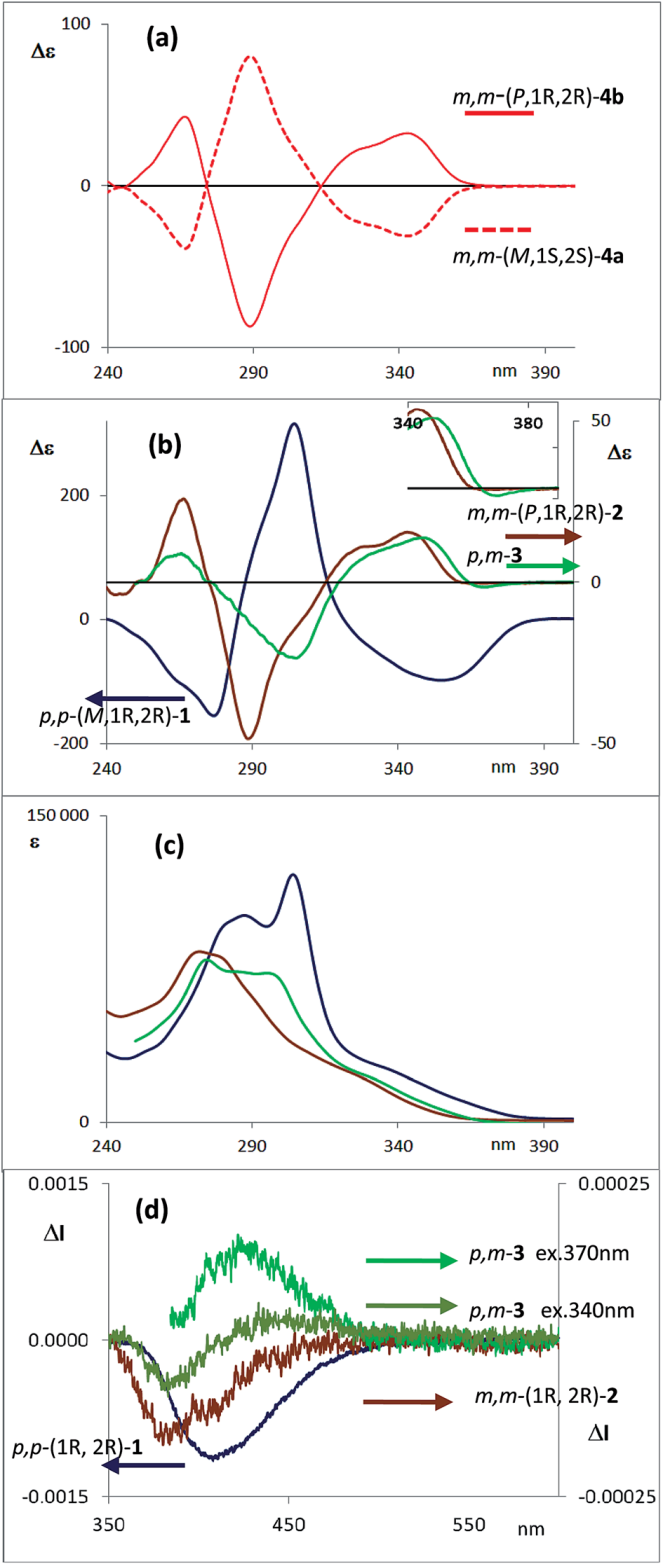
(a) CD spectra of diastereoisomers **4a** (1′*S*,4′*R*,*M*,1*S*,2*S*) and **4b** (1′*S*,4′*R*,*P*,1*R*,2*R*). (b) CD spectra of one enantiomer of each compound **1–3** (configuration 1*R*,2*R* for compounds **1** and **2**). (c) Absorption spectra of compounds **1–3**. (d) CPL spectra of compounds **1**, **2** and **3**, with the same enantiomers as the CD spectra presented above. Two excitation wavelengths are given for the CPL spectra of **3**.

Transesterification of **4a** afforded free diol *m*,*m*-(*M*,1*S*,2*S*)-**2**, identical to one of the enantiomers of *m*,*m*-**2** previously obtained from HPLC resolution. The (*P*,1*R*,2*R*) absolute configuration could thus be assigned to the corresponding enantiomer of *m*,*m*-**2** and (1′*S*,4′*R*,*P*,1*R*,2*R*) to **4b**. The CD spectra of both diastereoisomers **4a** and **4b** in CH_2_Cl_2_ (ref. 11) are depicted in Fig. 3a. Compounds *m*,*m*-**4a** and *m*,*m*-**4b** behaved as pseudoenantiomers, showing that the configuration of the camphanoyl moiety does not significantly affect their chiroptical response. The relationship between the absolute configuration of the simple [*n*]-helicenes and the sign of the first intense band^12^ at the longest wavelength of the CD spectra has been pointed out.^13^ With these precedents and on the basis of TD-DFT calculations on the geometry obtained from optimization of the X-ray structure for compound **4a** (see ESI‡), we may correlate the negative Cotton effect at *λ* = 345 nm (Fig. 3a) with its *M* helical configuration.

The corresponding CD spectra of compounds **1–3** are shown in Fig. 3b (one enantiomer). Since alcohols absorb at *λ* < 200 nm, the CD bands observed in compounds **1–3** at *λ* > 240 nm must be due to the presence of new elements of chirality with a defined absolute configuration. While the band at *ca.* 260 nm is attributed to the conjugated triple bond transitions, the Cotton effects at 280–300 nm and 340–370 nm evidence a transfer of chirality from the stereogenic centers to the aromatic fragments, thus suggesting a helical chiral structure in solution. The CD signals are also maintained in different solvents and at different temperatures, suggesting that the helical structure is preserved in any case (see ESI‡). The high Δ*ε* values, especially for compounds **1** and **4**, are also consistent with the presence of such helical chirality and suggest a significant contribution of the helical aromatic framework to these chiral absorptions.

The helix sense can be established as follows: the presence of a Cotton effect of the same sign for the 340–370 nm band in *m*,*m*-**4a** and *p*,*p*-**1** (*λ* = 360 nm, Fig. 3a and b) suggested the same *M* absolute configuration for both structures. The positive Cotton effect appearing at *λ* = 345 nm for *m*,*m*-**4b** confirms the *P* absolute configuration previously assigned for this camphanoyl diastereoisomer. In turn, the positive bands at *ca.* 350 nm observed for *m*,*m*-**2** and *p*,*m*-**3** assigned the *P* absolute configuration to their helices, whereas the negative one supported the *M* configuration for their enantiomers (see ESI‡). The induced helicity can be correlated with the relative regiochemistry of the substituents of the aryl rings involved in the staple, as we have previously observed.^9^ Comparison of the CD spectra of compounds **1–4** evidences significantly lower intensities of enantiopure *m*,*m*-**2** and *p*,*m*-**3**.

**Fig. 4:**
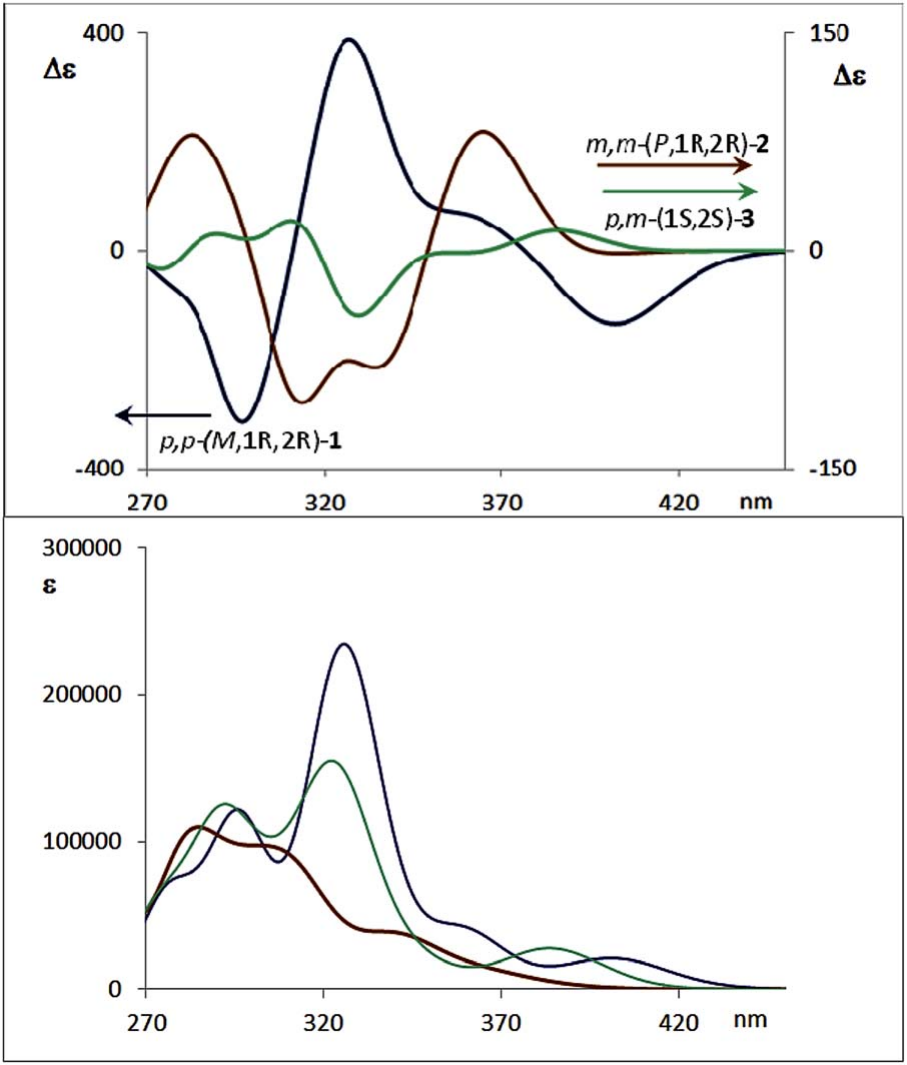
Average calculated CD (top) and absorption spectra (bottom) of the main conformers for *p*,*p*-**1** (blue), *m*,*m*-**2** (brown), and *m*,*p*-**3** (green) in CH_2_Cl_2_ (average on energy). The calculated spectra are at about 30 nm higher wavelengths than observed.

The dissymmetry value *g*_abs_ of **1** in CH_2_Cl_2_ (0.96 × 10^–2^) was one order of magnitude higher than those obtained for **2** (1.9 × 10^–3^) and **3** (1.5 × 10^–3^). The weaker chiroptical responses of **2** and **3** could be a consequence of the presence of two opposite helicities and/or the possible existence of a variety of quite distorted structures. To provide support for all the experimental evidences, and the stereoselectivity generated upon the metathesis/dihydroxylation stapling process, DFT calculations were conducted to determine the energy of the different diastereoisomers (see ESI for details‡). A molecular mechanics (MM) conformational search was performed for molecules **1**, **2** and **3**. Conformers within 5 kcal mol^–1^ were all optimized at B3LYP/6-31g* level. Populated conformers were also optimized within the framework of polarizable continuum model approximation (PCM), checking that all structures correspond to minima and evaluating the Gibbs free energy; the LANL2DZ pseudopotential was used for Ag(i). Thus, the diastereoisomer *p*,*p*-(*P*,1*S*,2*S*)-**1** is energetically favored by about 2 kcal mol^–1^ with respect to the *M* helical epimer (*M*,1*S*,2*S*). Thus, the calculated energies supported that *p*,*p*- substitution in the final aromatic ring of the stapled OPE **1** favors the *P* helical configuration in the case of *S*,*S* configured diols and *M* for *R*,*R* diols. The calculated CD spectrum (Fig. 4, top) is in agreement with the helicity previously assigned on the basis of the experimental CD. Taking into account these findings, we could assign the *p*,*p*-(*M*,1*R*,2*R*)-**1** absolute configuration to the enantiomer whose CD and CPL are included in Fig. 3, and the *p*,*p*-(*P*,1*S*,2*S*)-**1** absolute configuration to its enantiomer. For diols **2** and **3**, however, the situation is more complex because the calculated energies for the *P* and *M* diastereoisomers are very close (see ESI for details‡), suggesting that both helical epimers could coexist in solution. This situation could be at the origin of the lower intensity of the chiroptical responses observed in both the *m*,*m*-**2** and *p*,*m*-**3** derivatives. In spite of this, the calculated energies definitely supported that *m*,*m* substitution in **2**, considering all the possible conformer populations, slightly favors a *P* helix for *R*,*R* configured diols and *M* for *S*,*S* diols (see ESI for details‡), which is in agreement with the X-ray structure of **4a**.

Moreover, the chemical correlation of *m*,*m*-(*M*,1*S*,2*S*)-**2** resulting from transesterification of *m*,*m*-(1′*S*,4′*R*,*M*,1*S*,2*S*)-**4a** allows its unequivocal assignment. The CD spectrum shown in Fig. 3b corresponds to the *m*,*m*-(*P*,1*R*,2*R*)-**2** enantiomer. The presence of only one set of signals in the corresponding ^1^H-NMR spectrum of *m*,*m*-**2** at room temperature suggests a low *P*/*M* epimerization barrier. Moreover, low temperature NMR experiments for diols **1–3** down to –80 °C only show a broadening of the signals (see ESI‡), suggesting that a rapid equilibrium between the *P* and *M* helical structures might occur at room temperature. These data also support the epimerization barriers being low. Therefore the chiroptical responses emerge from the differences in energy of these dynamic structures.

The configurational assignment of *p*,*m*-**3** is less clear since the spectroscopic behavior strongly suggested a rapid interconversion between both helical conformers. Besides NMR experiments, some other experimental observations support such a dynamic situation. While **1** shows only one CD band at 360 nm (Fig. 3b), diol **3** presents a sequence of oppositely signed bands centered at 350 and 370 nm (a weaker negative band at lower energy and a stronger positive band at higher energy, Fig. 3b, inset). Such a pattern suggests the coexistence of both epimeric *P* and *M* helices in solution. In fact, a good correspondence with experiment can be obtained considering the calculated weight of all the energetically accessible conformations and the corresponding calculated CD spectra (Fig. 4b and ESI‡). Moreover, the ratio between the intensity of these two bands in the CD spectra of *p*,*m*-**3** is modified in different solvents, supporting the idea of a *P*/*M* epimerization process (Fig. S13, ESI‡). On the other hand, the unique helical configuration formed in *o*-OPEs **4**, and observed also in the crystal structure, must be due to the presence of bulky camphanoyl groups. Such groups make the structure more rigid, hindering any interconversion between *M* and *P* helimers and stabilizing one over the other.

Concerning the fluorescence properties, the three diols **1–3** are fluorescent with quantum yields and lifetimes highly dependent on the structure and solvent (Table 1). The experimental protocol for their measurement is described in the ESI.‡

**Table 1:** Quantum yields and lifetimes of compounds **1–3**

Solvent	*p*,*p*-1			*m*,*m*-2			*p*,*m*-3		
	*Φ*	*τ*_1_ ± SD (ns)	*τ*_2_ ± SD (ns)	*Φ*	*τ*_1_ ± SD (ns)	*τ*_2_ ± SD (ns)	*Φ*	*τ*_1_ ± SD (ns)	*τ*_2_ ± SD (ns)
Dichloromethane	0.069	4.86 ± 0.02	1.15 ± 0.02	0.181	4.16 ± 0.06	1.08 ± 0.01	0.520	4.54 ± 0.02	1.08 ± 0.02
Acetonitrile	0.065	5.64 ± 0.02	1.90 ± 0.04	0.144	4.73 ± 0.04	1.47 ± 0.03	0.356	5.27 ± 0.03	1.58 ± 0.05
Acetone	0.056	5.14 ± 0.02	1.52 ± 0.04	0.029	3.76 ± 0.05	1.14 ± 0.03	0.531	4.74 ± 0.04	1.17 ± 0.03
THF	0.089	7.13 ± 0.04	2.29 ± 0.02	0.016	6.49 ± 0.05	1.88 ± 0.02	0.097	6.33 ± 0.04	1.8 ± 0.02
Diethyl ether	0.037	6.84 ± 0.05	3.43 ± 0.03	0.023	5.88 ± 0.06	1.38 ± 0.02	0.100	4.17 ± 0.02	0.85 ± 0.02
Ethyl acetate	0.068	8.41 ± 0.10	4.14 ± 0.02	0.011	5.82 ± 0.08	1.49 ± 0.04	0.117	4.85 ± 0.03	1.24 ± 0.03
Methanol	0.057	6.13 ± 0.07	1.54 ± 0.03	0.032	5.82 ± 0.05	1.90 ± 0.03	0.091	5.60 ± 0.04	1.78 ± 0.03
Hexane	0.136	4.60 ± 0.08	1.03 ± 0.01	0.014	2.96 ± 0.02	1.23 ± 0.01	0.055	3.36 ± 0.04	1.11 ± 0.02
Toluene	0.236	4.17 ± 0.02	0.70 ± 0.02	0.024	3.32 ± 0.05	0.94 ± 0.01	0.175	3.91 ± 0.03	0.72 ± 0.02

The CPL spectra of enantiopure *p*,*p*-**1**, *m*,*m*-**2** and *p*,*m*-**3** are shown in Fig. 3d. As expected, all the CPL spectra show an intense band at 370–450 nm for samples excited at 340 nm. Remarkably, the *g*_lum_ value for compound **1** is 0.011 (*λ* = 390 nm), which is one of the highest values ever reported for an organic-based monomolecular emitter.^1^ The conformational equilibrium of these compounds in the excited state is less clear than in the ground state. In this case, Time Resolved Emission Spectroscopy (TRES) allowed the complete deconvolution of the total emission spectrum by recovering the species-associated emission spectra (SAEMS) for each one of the decay times (see ESI‡). In all cases, we could confirm the presence of two main emissive species, generated by some degree of structural relaxation in the excited state. For compound **1**, the species with the larger decay time (*τ*_1_ = 4.86 ns) dominates the equilibrium. The second one (*τ*_2_ = 1.15 ns) could derive from some minor structural changes before emission to maintain the original helicity, owing to the excellent *g*_lum_ value. For compound **2**, only one component is significantly present with a shorter lifetime (*τ*_2_ = 1.08 ns), which also suggests that in this case structural relaxation takes place. Possibly, the presence of several quite distorted geometries for compound **2** is responsible for the short life time relaxation, and also for the fact that, on average, the helicity is inverted as suggested by the observed inversion of the CPL sign. The camphanoyl esters **4a** and **4b** derived from diol **2** show a related profile, and one emitting species is mainly present in the excited state (**4a**, *τ*_2_ = 0.86 ns, **4b**, *τ*_2_ = 0.84 ns). In compound **3**, two slightly wavelength biased emissive species are present. This fact, combined with the observed bisignated signal in the CD (Fig. 3b, inset) and the dependence on the excitation wavelength in CPL, suggests that they may derive from *P* and *M* epimers already present before excitation.

**Fig. 5:**
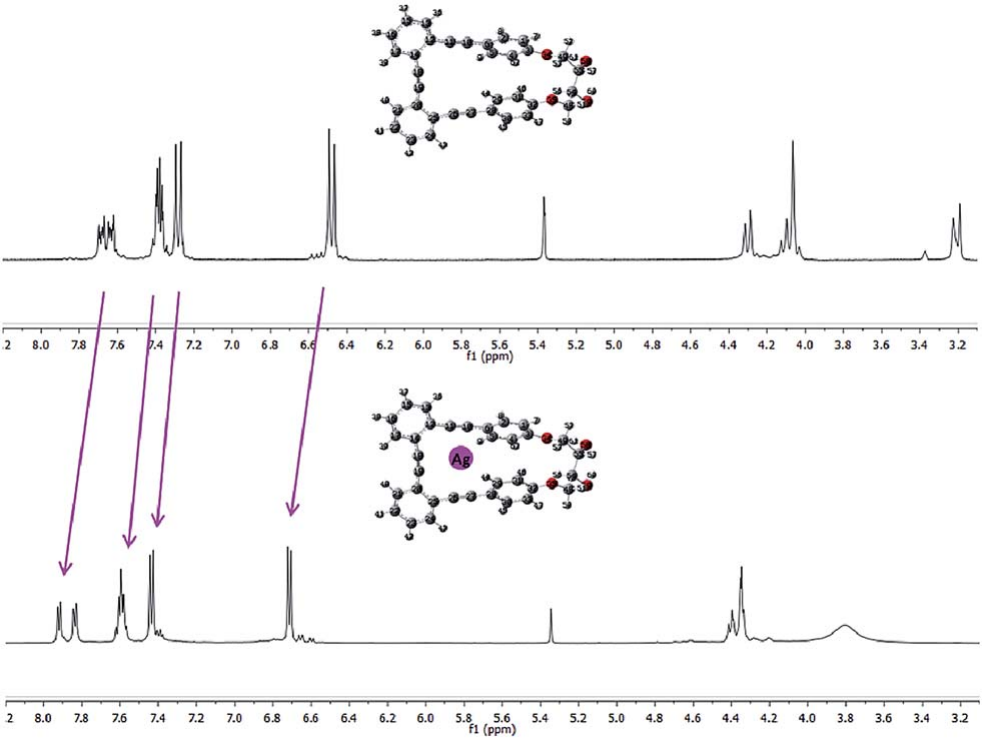
Representative example: spectra of pure *p*,*p*-**1** (top) and after saturation with Ag(i) (bottom) in a 9 : 1 CD_2_Cl_2_ : acetone-*d*_6_ mixture.

Essentially, a control of this dynamic situation observed for diols **1–3** would allow an efficient and reversible modulation of the intrinsic chiroptical properties of the system. Bearing this idea in mind, we next studied the binding ability of these helical *o*-OPEs. Remarkably, compounds **1–4** are able to accommodate Ag(i) in their inner cavities *via* carbophilic interactions, similar to what we previously described for related *o*-OPEs.^10^ The coordination behavior of compounds **1–4** with Ag(i) has been studied using NMR, CD and CPL spectroscopy. Significant changes in the ^1^H and ^13^C NMR signals are produced after addition of AgBF_4_, demonstrating that coordination occurs in all cases (Fig. 5 and ESI‡).

**Fig. 6:**
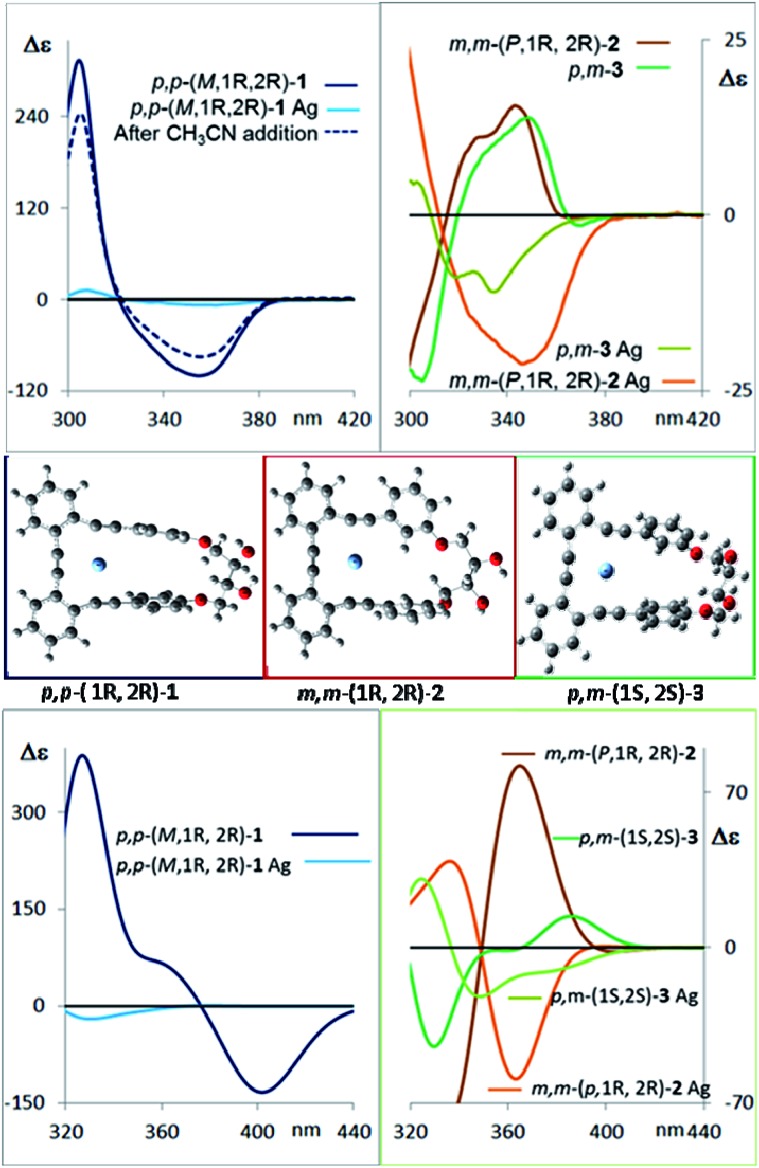
Top left: Measured CD spectra of the compounds *p*,*p*-**1** (blue), *p*,*p*-**1** saturated with Ag(i) (light blue) and recovery of the CD signal of *p*,*p*-**1** after CH_3_CN addition to the Ag(i) complex solution (dashed dark blue line). Top right: Measured CD spectra of the pure compounds *m*,*m*-**2** (dark brown) and *p*,*m*-**3** (dark green), and when saturation with Ag(i) is observed (light brown and green). Middle: Calculated structures of the most populated conformer of the silver complexes of diols **1–3**. Bottom: Comparison of the average calculated CD spectra of the main conformers for diols and Ag(i) complexes of *p*,*p*-**1**(blue), *m*,*m*-**2** (brown), and *m*,*p*-**3** (green) in CH_2_Cl_2_. The calculated spectra are at about 30 nm higher wavelengths than observed.

CD spectroscopy evidences very remarkable changes in the signal intensities upon addition of Ag(i) to compounds **1–3** (Fig. 6, top). This might be a consequence of a change from the helical structure to a more planar trigonal arrangement, forced by the alkyne interaction with Ag(i). A similar structural behavior had been previously observed by us in the X-ray structures of closely related complexes.^10^ DFT calculations (Fig. 6, middle and ESI‡) of the Ag(i)-complexed structures **1–3** confirm this lack of helicity. Titration curves give access to the binding constants of the corresponding complexes *o*-OPEs:Ag(i) with the following values: (**1**) *K***_1_**_:Ag_ = 12 211 ± 635 M^–1^, (**2**) *K***_2_**_:Ag_ = 4805 ± 161 M^–1^, (**3**) *K***_3_**_:Ag_ = 35 926 ± 1064 M^–1^ (**4a**), *K***_4a_**_:Ag_ = 1099 ± 42 M^–1^ (**4b**), *K***_4b_**_:Ag_ = 466 ± 11 M^–1^. Although all three diols interact with Ag(i) cations, the more symmetric derivative **1** shows the best geometry for Ag(i) accommodation. Moreover, the almost planar geometry of the **1**:Ag(i) complex ensures a very weak chiroptical response, as can be seen in the CD spectrum (Fig. 6 top). The **1**:Ag(i) complex signal (light blue line) is almost one order of magnitude less intense than that of pure **1** (blue line). Moreover, the original CD signals can be easily recovered by simple addition of a stoichiometric amount of CH_3_CN to the solution containing the Ag(i) complexes (Fig. 6 top, blue dashed line).

**Fig. 7:**
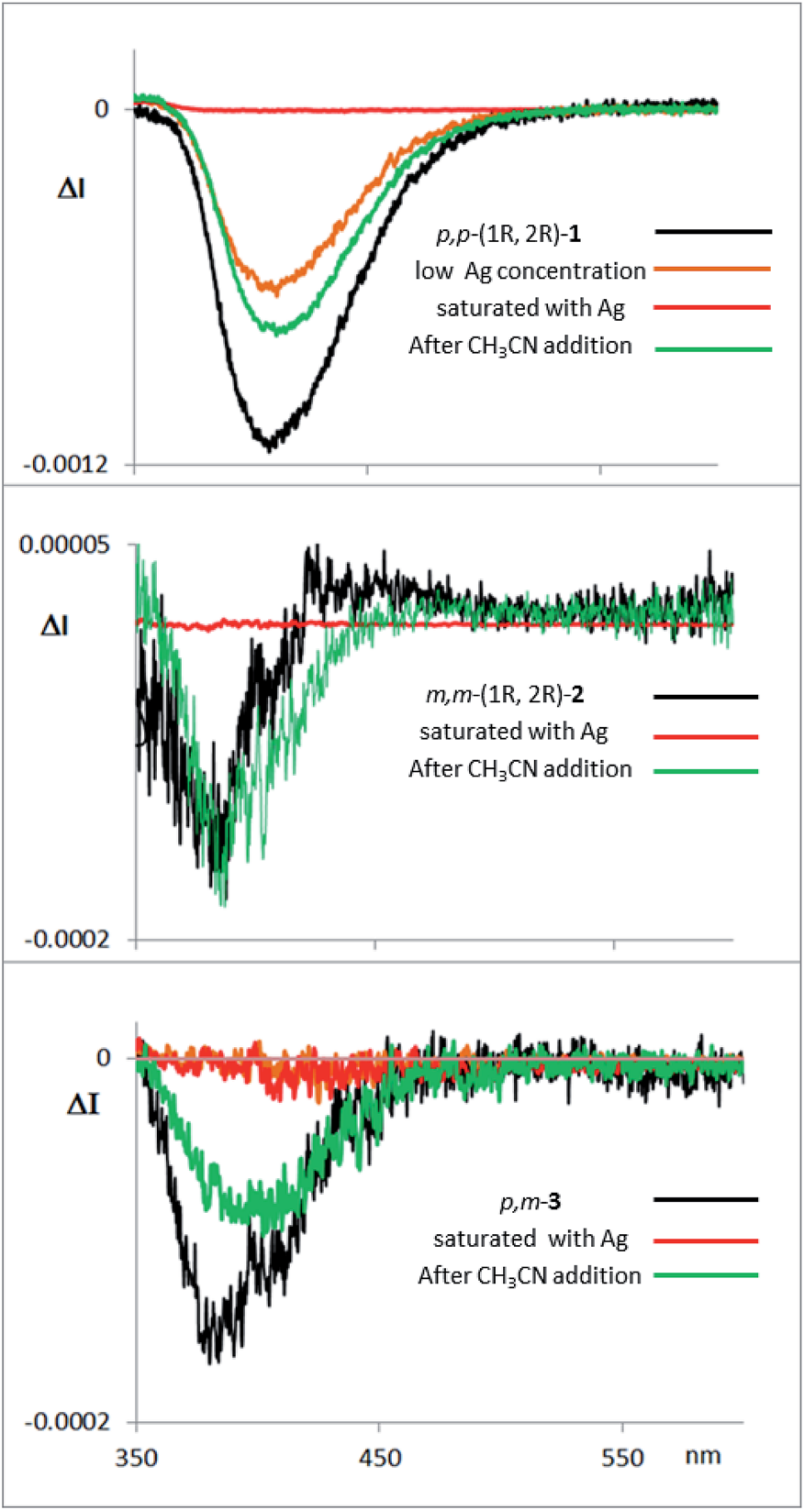
CPL spectra of compounds **1**, **2** and **3**, with them same enantiomer as the CD and CPL spectra presented above in Fig. 3d. The compounds were measured in CH_2_Cl_2_, with addition of AgBF_4_ until saturation, and with further addition of CH_3_CN thereafter.

Fluorescence and CPL studies of the Ag(i) complexes also confirm the above observations (see ESI‡). In the presence of Ag(i), the CPL signal almost disappears for compound **1**, supporting the formation of a planar structure in the excited state, which switched off the chiral helical component and therefore silences the CD or CPL response from the helicity (Fig. 7). For compounds **2** and **3** the signals are also significantly changed, suggesting a change in the geometry of the excited state. Interestingly, the CPL signals can be recovered by adding a stoichiometric amount of CH_3_CN to the solution containing the Ag(i) complexes (Fig. 7), thus making these systems the first CPL switches based on carbophilic interactions. The neat modification of the CD and CPL signals of these helical structures when interacting with Ag(i) opens the possibility of using them as Ag(i)-sensitive chiroluminescent products.

Finally, the photostability of compounds **1–4** was also studied (see ESI‡), and suggested that partial degradation of the samples under irradiation during CPL acquisition could be the responsible for the incomplete recovery of the CPL signal intensity (Fig. S3, S4 and S19‡).

## Conclusions

In summary, new enantiopure *o*-OPE derivatives **1–4** have been synthesized and their chiroptical properties have also been studied. All investigated structures show excellent CD and CPL responses as well as reasonable quantum yields. In particular, *o*-OPE *p*,*p*-**1** featured a *g*_lum_ = 0.011, which is one of the highest values described up to now for a small organic compound. These stapled compounds represent a promising new class of CPL emitters with great structural versatility and easy access, opening the possibility of developing new CPL emitters with improved and novel applications. It is also worth noting that these stapled OPEs combine a defined helical structure with a flexible inner core, which makes them useful in dynamic chiral photoresponses. This fact has been exemplified in the interaction with Ag(i), which acts as a reversible silencer of their chiral CD or CPL responses.

## Supplementary Material

Supplementary information

Crystal structure data

## References

[cit1] Sanchez-Carnerero E. M., Agarrabeitia A. R., Moreno F., Maroto B. L., Muller G., Ortiz M. J., de la Moya S., Castiglioni E., Abbate S., Longhi G., Castiglioni E., Abbate S., Lebon F., Longhi G., Berova N., Di Bari L., Pescitelli G., Kumar J., Nakashima T., Kawai T. (2015). Chem.–Eur. J..

[cit2] For selected examples of helicene-based CPL-active compounds: Phillips K. E. S. Katz T. J. Jockusch S. Lovinger A. J. Turro N. J., J. Am. Chem. Soc., 2001, 123 , 11899 –11907 Field J. E. Muller G. Riehl J. P. Venkataraman D., J. Am. Chem. Soc., 2003, 125 , 11808 –11809 Kaseyama T. Furumi S. Zhang X. Tanaka K. Takeuchi M., Angew. Chem., Int. Ed., 2011, 50 , 3684 –3687 Sawada Y. Furumi S. Takai A. Takeuchi M. Noguchi K. Tanaka K., J. Am. Chem. Soc., 2012, 134 , 4080 –4083 Abbate S. Longhi G. Lebon F. Castiglioni E. Superchi S. Pisani L. Fontana F. Torricelli F. Caronna T. Villani C. Sabia R. Tommasini M. Lucotti A. Mendola D. Mele A. Lightner D. A., J. Phys. Chem. C, 2014, 118 , 1682 –1695 Nakamura K. Furimi S. Takeuchi M. Shibuja T. Tanaka K., J. Am. Chem. Soc., 2014, 136 , 5555 –5558 Shen C. Anger E. Srebro M. Vanthuyne N. Deol K. K. Jefferson Jr T. D. Muller G. Williams J. A. G. Toupet L. Roussel C. Autschbach J. Réau R. Crassous J., Chem. Sci., 2014, 5 , 1915 –1927 Saleh N. Srebro M. Reynaldo T. Vanthuyne N. Toupet L. Chang V. Y. Muller G. Willians J. A. G. Roussel C. Autschbach J. Crassous J., Chem. Commun., 2015, 51 , 3754 –3757 Murayama K. Oike Y. Furimi S. Takeuchi M. Noguchi K. Tanaka K., Eur. J. Org. Chem., 2015 , 1409 –1414 Matsuno T. Koyama Y. Hiroto S. Kumar J. Kawai T. Shinokubo H., Chem. Commun., 2015, 51 , 4607 –4610 Longhi G. Castiglioni E. Villani C. Sabia R. Menichetti S. Viglianisi C. Devlin F. Abbate S., J. Photochem. Photobiol., A, 201610.1016/j.jphotochem.2015.12.011 Yamamoto Y. Sakai H. Yuasa J. Araki Y. Wada T. Sakanoue T. Takenobu T. Kawai T. Hasobe T., J. Phys. Chem. C, 2016, 120 , 7421 –7427 .11724596

[cit3] Other selected CPL-active organic compounds: Sannicolò F. Mussini P. R. Benincori T. Cirilli R. Abbate S. Arnaboldi S. Casolo S. Castiglioni E. Longhi G. Martinazzo R. Panigati M. Pappini M. Procopio E. Q. Rizzo S., Chem.–Eur. J., 2014, 20 , 15298 –15302 Amako T. Nakabayashi K. Mori T. Inoue Y. Fujiki M. Imai Y., Chem. Commun., 2014, 50 , 12836 –12839 Inouye M. Hayashi K. Yonenaga Y. Itou T. Fujimoto K. Uchida T. Iwamura M. Nozaki K., Angew. Chem., Int. Ed., 2014, 53 , 14392 –14396 Kitayama Y. Amako T. Suzuki N. Fujiki M. Imai Y., Org. Biomol. Chem., 2014, 12 , 4342 –4346 Longhi G. Abbate S. Mazzeo G. Catiglioni E. Mussini P. Benincori T. Martinazzoand R. Sannicolò F., J. Phys. Chem. C, 2014, 118 , 16019 –16027 Morisaki Y. Gon M. Sasamori T. Tokitoh N. Chujo Y., J. Am. Chem. Soc., 2014, 136 , 3350 –3353 Gon M. Morisaki Y. Chujo Y., J. Mater. Chem. C, 2015, 3 , 521 –529 Morisaki Y. Inoshita K. Chujo Y., Chem.–Eur. J., 2014, 20 , 8386 –8390 Gon M. Morisaki Y. Chujo Y., Eur. J. Org. Chem., 2015 , 7756 –7762 Gon M. Morisaki Y. Sawada R. Chujo Y., Chem.–Eur. J., 2016, 22 , 2291 –2298 Morisue M. Yumura T. Sawada R. Naito M. Kuroda Y. Chujo Y., Chem. Commun., 2016, 52 , 2481 –2484 Sanchez-Carnerero E. M. Moreno F. Maroto B. L. Agarrabeitia A. R. Ortiz M. J. Vo B. G. Muller G. de la Moya S., J. Am. Chem. Soc., 2014, 136 , 3346 –3349 Kögel J. F. Kusaka S. Sakamoto R. Iwashima T. Tsuchiya M. Toyoda R. Matsuoka R. Tsukamoto T. Yuasa J. Kitagawa Y. Kawai T. Nishihara H., Angew. Chem., Int. Ed., 2016, 55 , 1377 –1381 Nakabayashi K. Kitamura S. Suzuki N. Guo S. Fujiki M. Imai Y., Eur. J. Org. Chem., 2016 , 64 –69 Alnoman R. B. Rihn S. ÓConnor D. C. Black F. A. Costello B. Waddell P. G. Clegg W. Peacock R. D. Herrebout W. Knight J. G. Hall M. J., Chem.–Eur. J., 2016, 22 , 93 –96 Murai M. Takeuchi Y. Yamauchi K. Kuninobu Y. Takai K., Chem.–Eur. J., 2016, 22 , 6048 –6058 . 25263106 10.1002/chem.201404331 PMC4271670

[cit4] Zinna F., Resta C., Abbate S., Castiglioni E., Longhi G., Mineo P., Di Bari L., Heffern M. C., Matosziuk L. M., Meade T. J., Carr R., Evans N. H., Parker D., Muller G., Lunkley J. L., Shirotani D., Yamanari K., Kaizaki S., Muller G. (2015). Chem. Commun..

[cit5] For selected examples of CPL emitting organic polymers and aggregates: Peeters E. Christiaans M. P. T. Janssen H. P. Schoo H. F. Dekkers H. P. J. M. Meijer E. W., J. Am. Chem. Soc., 1997, 119 , 9909 –9910 Yang Y. Correa da Costa R. Smilgies D.-M. Campbell A. J. Fuchter M. J., Adv. Mater., 2013, 25 , 2624 –2628 Kumar J. Nakashima T. Tsumatori H. Kawai T., J. Phys. Chem. Lett., 2014, 5 , 316 –321 Liu J. Su H. Meng L. Zhao Y. Deng C. Ng J. C. Y. Lu P. Faisal M. Lam J. W. Y. Huang X. Wu H. Wong K. S. Tang B. Z., Chem. Sci., 2012, 3 , 2737 –2747 San Jose B. A. Yan J. Akagi K., Angew. Chem., Int. Ed., 2014, 53 , 10641 –10644 Shen Z. Wang T. Shi L. Tang Z. Liu M., Chem. Sci., 2015, 6 , 4267 –4272 Kumar J. Tsumatori H. Yuasa J. Kawai T. Nakashima T., Angew. Chem., Int. Ed., 2015, 54 , 5943 –5947 Watanabe K. Koyama Y. Suzuki N. Fujiki M. Nakano T., Polym. Chem., 2014, 5 , 712 –717 Fujiki M. Jalilah A. J. Suzuki N. Taguchi M. Zhang W. Abdellatif M. M. Nomura K., RSC Adv., 2012, 2 , 6663 –6671 Nakano Y. Fujiki M., Macromolecules, 2011, 44 , 7511 –7519 .

[cit6] Jeong S. M., Ohtsuka Y., Ha N. Y., Takanishi Y., Ishikawa K., Takezoe H., Nichimura S., Suzaki G. (2007). Appl. Phys. Lett..

[cit7] Sapir M., Donckt E. V., Grellmann K.-H., Hentzschel P., Wismontski-Knittel T., Fischer E. J. (1975). Chem. Phys. Lett..

[cit8] Maeda H., Bando Y., Shimomura K., Yamada I., Naito M., Nobusawa K., Tsumatori H., Kawai T., Haketa Y., Bando Y., Takaishi K., Uchiyama M., Muranaka A., Naito M., Shibaguchi H., Kawai T., Maeda H., Maeda H., Bando Y., Maeda H., Shirai T., Bando Y., Takaishi K., Uchiyama M., Muranaka A., Kawai T., Naito M., Saleh N., Moore B., Srebro M., Vanthuyne N., Toupet L., Williams J. A. G., Roussel C., Deol K. K., Muller G., Autschbach J., Crassous J., Hashimoto Y., Nakashima T., Shimizu D., Kawai T., Isla H., Srebro-Hooper M., Jean M., Vanthuyne N., Roisnel T., Lunkley J. L., Muller G., Willians J. A. G., Autschbach J., Crassous J. (2011). J. Am. Chem. Soc..

[cit9] Fuentes N., Martín-Lasanta A., Álvarez de Cienfuegos L., Robles R., Choquesillo-Lazarte D., García-Ruiz J. M., Mota A. J., Martínez-Fernández L., Corral I., Cárdenas D. J., Ribagorda M., Carreño M. C., Cuerva J. M. (2012). Angew. Chem., Int. Ed..

[cit10] Martín-Lasanta A., Álvarez de Cienfuegos L., Johnson A., Miguel D., Mota A. J., Orte A., Ruedas-Rama M. J., Ribagorda M., Cárdenas D. J., Carreño M. C., Echavarren A. M., Cuerva J. M. (2014). Chem. Sci..

[cit11] CH2Cl2 was chosen as solvent to avoid solubility problems and also to carry out the coordination experiments with Ag(i) cation.

[cit12] Nakai Y., Mori T., Inoue Y. (2013). J. Phys. Chem. A.

[cit13] Furche F., Ahlrichs R., Wachsmann C., Weber E., Sobanski A., Vögtle F., Grimme S. (2000). J. Am. Chem. Soc..

