# Supplementary material for: Stapled helical o-OPE foldamers as new circularly polarized luminescence emitters based on carbophilic interactions with Ag(i)-sensitivity
Source: Chem Sci. 2016 May 17;7(9):5663–70. doi: 10.1039/c6sc01808d (PMC6022022; doi:10.1039/c6sc01808d)
Supplement: Supplementary file 1 [file SC-007-C6SC01808D-s001.pdf]

Electronic Supplementary Material (ESI) for Chemical Science.
This journal is © The Royal Society of Chemistry 2016

Electronic Supplementary Information (ESI)

Stapled helical o-OPEs as new Circularly Polarized Luminescence emitters based on carbophilic interactions with Ag(I)-sensitivity

Sara P. Morcillo,[a] Delia Miguel[b,*] Luis Álvarez de Cienfuegos,[b] José Justicia,[b] Sergio Abbate,[c] Ettore Castiglioni,[c] Christophe Bour,[a] María Ribagorda,[d] Diego J. Cárdenas,[d] José Manuel Paredes,[e] Luis Crovetto,[e] Duane Choquesillo-Lazarte,[f] Antonio J. Mota,[g] M. Carmen Carreño,[d] Giovanna Longhi,[c,*] and Juan M. Cuerva[b,*]

[a.] *Institut de Chimie Moléculaire et des Matériaux d'Orsay, CNRS UMR 8182, Univ. Paris-Sud Université Paris-Saclaybâtiment 420, 91405 Orsay cedex (France)*

[b.] *Department of Organic Chemistry, University of Granada (UGR). C. U. Fuentenueva, 18071 Granada, Spain. email: jmcuerva@ugr.es, lac@ugr.es*

[c.] *Dipartimento di Medicina e Traslazionale, Università di Brescia. Viale Europa 11 25123 Brescia, Italy. email: giovanna.longhi@unibs.it.*

[d.] *Departamento de Química Orgánica, Universidad Autónoma de Madrid. C/Francisco Tomás y Valiente nº 7, Cantoblanco, 28049 Madrid, Spain.*

[e.] *Department of Physical Chemistry, Faculty of Pharmacy, UGR. Cartuja Campus, 18071Granada, Spain.*

[f.] *Laboratorio de Estudios Cristalográficos, Instituto Andaluz de Ciencias de la Tierra (CSIC-UGR), 18100 Armilla, Granada, Spain.*

[g.] *Department of Inorganic Chemistry, UGR. C. U. Fuentenueva s/n. 18071 Granada, Spain.*

Table of Contents

- General details, procedures and spectroscopic data of new compounds	S2-S10
- ^{1}H and ^{13}C NMR spectra of new compounds	S11-S27
- Low temperature ^{1}H-NMR of compounds **1-3**	S28-29
- Preparative chiral HPLC resolution	S30
- Single crystal X-ray analysis	S31
- CD and CPL measurements	S32-S39
- CD spectra of compounds **1-3** in different solvents and temperatures	S40-S41
- Lifetimes, quantum yields, TRES and photostability of compounds**1-4**	S42-46
-NMR titrations of compounds **1-4** with Ag(I): General procedure, spectroscopic data and copies of ^{1}H-NMR and ^{13}C-NMR spectra of Ag(I)-diol complexes	S47-53
- CD titrations of compounds **1-4** with Ag(I)	S54-59
-Theoretical calculations	S60-74
- References	S75

SYNTHETIC PART

General Details

The following palladium catalysts, *trans*-dichlorobis(triphenylphosphine)palladium(II) ($Pd(PPh_3)_2Cl_2$), and *trans*-dichlorobis(acetonitrile)palladium(II) ($Pd(CH_3CN)_2Cl_2$), were prepared from palladium(II) chloride ($PdCl_2$) according to previously described procedures.[1]Palladium(II) chloride ($PdCl_2$), copper(I) iodide (CuI), trimethylsilyl acetylene (TMSA), triethylamine (Et_3N), *N*,*N*-diisopropylamine (*i*Pr_2NH), tri-*tert*-butylphosphonium tetrafluoroborate (*t*$Bu_3P·HBF_4$),compounds **5** and **13** andall other reagents were directly used from standard chemical suppliers. TLC was performed on aluminium-backed plates coated with silica gel 60 (230-240 mesh) with F254 indicator. The spots were visualized with UV light (254 nm). All chromatography purifications were performed with silica gel 60 (40-60 μm). NMR spectra were measured at room temperature with the exception of diols 1-3. ^{1}H NMR spectra were recorded at 300, 400, 500 or 600 MHz. Chemical shifts are reported in ppm using residual solvent peak as reference ($CHCl_3$: δ = 7.26 ppm, CH_2Cl_2: δ = 5.32 ppm, $(CH_3)_2CO$: δ = 2.05 ppm). Data are reported as follows: chemical shift, multiplicity (s: singlet, d: doublet, t: triplet, q: quartet, quint: quintuplet, m: multiplet, dd: doublet of doublets, dt: doublet of triplets, dq: doublet of quartets, td: triplet of doublets, bs: broad singlet), coupling constant (*J* in Hz) and integration; ^{13}C NMR spectra were recorded at 75, 100, 125 or 150 MHz using broadband proton decoupling, and chemical shifts are reported in ppm using residual solvent peaks as reference ($CHCl_3$: δ = 77.16 ppm, CH_2Cl_2: δ = 54.0 ppm, $(CH_3)_2CO$: δ = 29.84 ppm). Carbon multiplicities were assigned by DEPT techniques. High resolution mass spectra (HRMS) were recorded on a mass spectrometer using EI at 70eV. The following known compounds were isolated as pure samples and showed NMR spectra matching those of previously reported compounds: **6-8**,[2] and **10**.[2]

General Procedures (GP)

Representative protocol for Sonogashira coupling of aryl iodides (GP1):

A solution of the terminal alkyne (1.1 mmol) dissolved in the minimum amount of THF and 2 mL of Et_3N, was added dropwise to a carefully deoxigenated solution of

$Pd(PPh_3)_2Cl_2$ (5 mol % eq), CuI (10 mol %) and the aryl iodide (2 mmol for each terminal alkyne) in 10 mL of Et_3N. The reaction was stirred for 4 h at 60 ºC under argon atmosphere. The mixture was then diluted with EtOAc (40 mL), washed with saturated aq NH_4Cl (3 x 15 mL), dried over anhydrous Na_2SO_4, and the solvent was removed. The residue was purified by flash chromatography (EtOAc/Hexane mixtures) to give the corresponding coupling product.

Representative protocol for Sonogashira coupling of aryl bromides (GP2):

A solution of the terminal alkyne (1.1 mmol) dissolved in the minimum amount of THF and 2 mL of *i*Pr_2NH, was added dropwise to a carefully deoxigenated solution of $Pd(CH_3CN)_2Cl_2$ (3 mol %), *t*$Bu_3P{\cdot}HBF_4$ (6 mol %), CuI (3 mol %) and the aryl bromide (2 mmol for each terminal alkyne) in 10 mL of *i*Pr_2NH. The reaction was stirred 4 h at room temperature under argon atmosphere. The mixture was then diluted with EtOAc (40 mL), washed with saturated aq NH_4Cl (3 x 15 mL), dried over anhydrous Na_2SO_4, and the solvent was removed. The residue was purified by flash chromatography (EtOAc/Hexane mixtures) to give the corresponding coupling product.

General procedure for removing protecting silyl groups (GP3):

To a solution of the starting silylether (1mmol) in THF (10ml), TBAF (2 mmol) was added, and the mixture was stirred at room temperature until complete consumption of the starting material (TLC, 1–4 h). The solution was diluted with EtOAc (50 mL) and washed with saturated aq NH_4Cl (3 x 15 mL). The organic layer was then dried over anhydrous Na_2SO_4, and the solvent removed. The residue was purified by flash chromatography on silica gel (EtOAc/Hexane mixtures) to afford the pure product.

General procedure for the phenol allylation reaction (GP4)

To a solution of the corresponding alcohol (1 mmol) in 10 ml of anhydrous DMF at room temperature, K_2CO_3 (6 mmol) was added portion wise and the solution was stirred for 10 min at room temperature. Then, allyl bromide (6 mmol) was added and the reaction mixture was stirred for 3 h. The reaction was then diluted with EtOAc (40 mL), washed with 2N HCl (3 x 20 mL), dried over anhydrous Na_2SO_4, and the

solvent removed. The residue was purified by flash chromatography on silica gel (EtOAc/Hexane mixtures), to afford the correspondingallylated product.

Representative protocolfor the *stapling* metathesis reaction (GP5)

A solution of the corresponding diallyl compound (1 mmol) in deoxigenated CH_2Cl_2, was added dropwise at room temperature to a solution of first generation Grubbs catalyst (0.1 mmol) in deoxigenated CH_2Cl_2 (10^{-3} M). The mixture was stirred at 45 ºC for 2 h under an argon atmosphere. The solvent was then removed, and the residue was purified by flash chromatography (EtOAc/Hexane mixtures) to give the corresponding stapled product.

Representative protocol for the dihydroxylation reaction using OsO_4 (GP6)

A solution of the starting alkene (1 mmol) in acetone (1 mL) was added to a mixture of *N*-methylmorpholine-*N*-oxide (1 mmol) and osmium tetroxide (0.2 mmol, 1 % solution in water) in acetone (10 mL). The reaction was monitored by TLC until consumption of the starting material (16-24 h). Then, a 20% solution of sodium metabisulphite (5 ml) was added to the mixture and stirred for 10 min. The mixture was extracted with EtOAc, dried over anhydrous Na_2SO_4, and the solvent removed.The residue was purified by flash chromatography (EtOAc/Hexane mixtures) to give the corresponding dihydroxylated product.

Scheme S-1. Synthesis of key intermediate **8**.

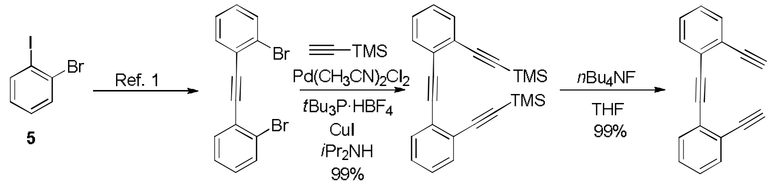

5 **6** **7** **8**

Scheme S-2. Synthesis of compounds *p*,*p*-**1** and *m*,*m*-**2** from **8**.

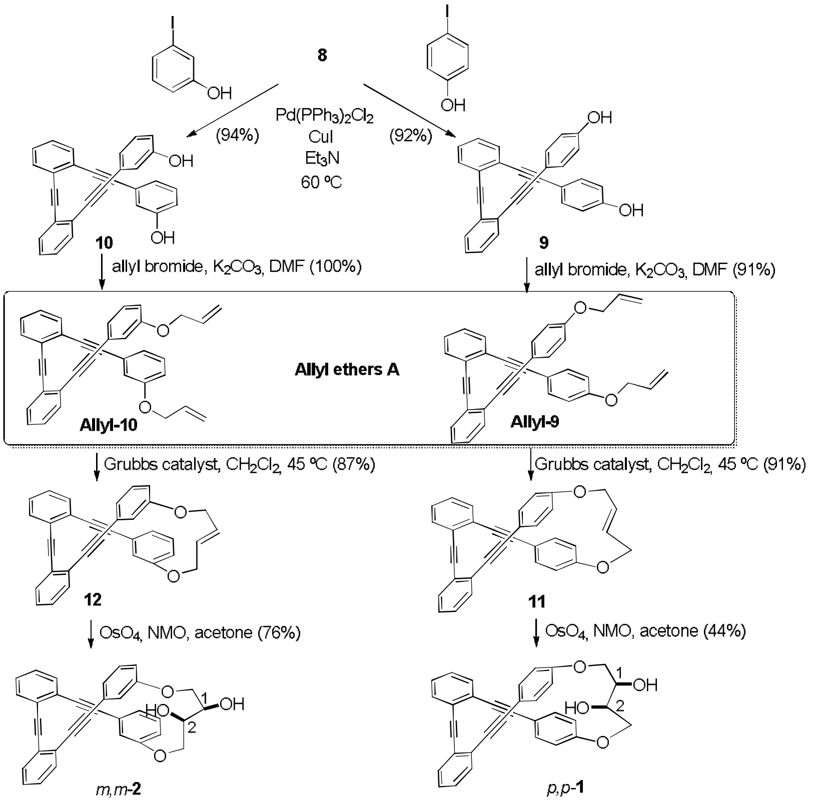

Scheme S-3. Preparation of compound *p*,*m*-**3.**

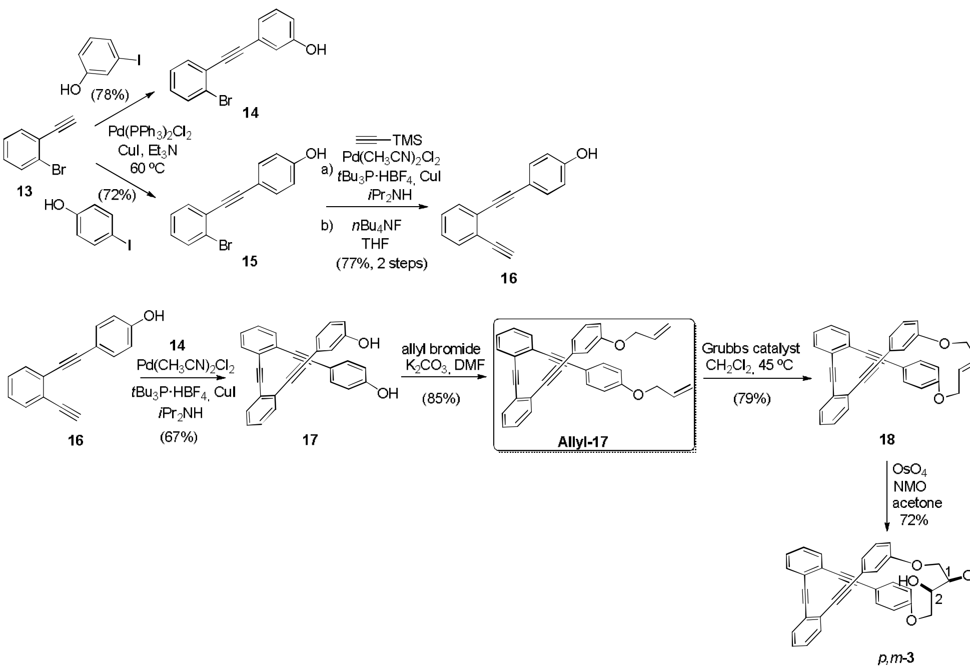

Compound 9: According to GP 1: White solid: ^{1}H NMR (400 MHz, $CDCl_3$) δ 7.63 (dd, *J* = 7.4, 1.0 Hz, 2H), 7.59 (dd, *J* = 7.4, 1.0 Hz, 2H), 7.47 (d, *J* = 8.6 Hz, 4H), 7.37–7.26 (m, 4H), 6.83 (d, *J* = 8.7 Hz, 4H); ^{13}C NMR (125 MHz, $CDCl_3$) δ 155.8 (C), 133.4 (CH), 132.0

(CH), 131.6 (CH), 128.0 (CH), 127.5 (CH), 126.1 (C), 125.8 (C), 115.6 (C), 115.4 (CH), 93.8 (C), 92.3 (C), 87.0 (C); HRMS-(ES): $(M)^+$m/z calcd for $C_{30}H_{18}O_2$, 410.1307; found 410.1312.

Allyl-9: According to GP4: Colourless oil: ^1H NMR(300 MHz, $CDCl_3$) δ 7.68–7.54 (m, 4H), 7.49 (d, *J* = 7.5 Hz, 4H), 7.41–7.26 (m, 4H), 6.85 (d, *J* = 7.5 Hz, 4H), 6.18–5.97 (m, 2H), 5.38 (dd, *J* = 17.3, 10.5 Hz, 4H), 4.56 (bs, 4H). ^{13}C NMR (75 MHz, $CDCl_3$) δ 155.8 (C), 133.3 (CH), 133.0 (CH), 132.2 (CH), 131.7 (CH), 128.2 (CH), 127.7 (CH), 126.3 (C), 125.8 (C), 118.0 (CH_2), 115.7 (C), 114.8 (CH), 94.0 (C), 92.4 (C), 87.3 (C), 68.9 (CH_2);HRMS-(ES): $(M+H)^+$m/z calcd for $C_{36}H_{27}O_2$, 491.2005; found 491.2012.

Allyl-10: According to GP4: White solid: ^1H NMR (400 MHz, $CDCl_3$) δ 7.64–7.57 (m, 4H), 7.36–7.28 (m, 4H), 7.21–7.11 (m, 4H), 7.08 (bs, 2H), 6.89–6.86 (m, 2H), 6.05-5.96 (m, 2H), 5.37 (dd, *J* = 17.3, 1.5 Hz, 2H), 5.31–5.21 (m, 2H), 4.41 (d, *J* = 5.2 Hz, 4H); ^{13}C NMR (100 MHz, $CDCl_3$) δ 158.4 (C), 133.1 (CH), 132.1 (CH), 131.8 (CH), 129.4 (CH), 128.3 (CH), 128.1 (CH), 126.0 (C), 125.9 (C), 124.5 (CH), 124.3 (C), 117.8 (CH_2), 116.9 (CH), 116.4 (CH), 94.0 (C), 92.3 (C), 88.2 (C), 68.8 (CH_2); HRMS-(ES): $(M+H)^+$m/z calcd for $C_{36}H_{27}O_2$, 491.2005; found 491.2008.

Compound 11: According to GP5: White solid. ^1H NMR (300 MHz, $CDCl_3$) δ 7.67-7.64 (m, 4H), 7.45–7.20 (m, 8H), 6.53 (d, *J* = 8.6 Hz, 4H), 5.60 (s, 2H), 4.65 (s, 4H); ^{13}C NMR (125 MHz, $CDCl_3$) δ 156.9 (C), 133.3 (CH), 133.3 (CH), 132.7 (CH), 129.7 (CH), 128.2 (CH), 127.6 (CH), 125.6 (C), 125.2(C), 116.4 (CH), 115.8 (CH), 94.0 (C), 92.2 (C), 87.4 (C), 67.1 (CH_2); HRMS-(ES): $(M+H)^+$m/z calcd for $C_{34}H_{23}O_2$, 463.1698; found 463.1688.*Trans*-stereoisomer was exclusively obtained. Configuration was corroborated by single crystal X-Ray structure.

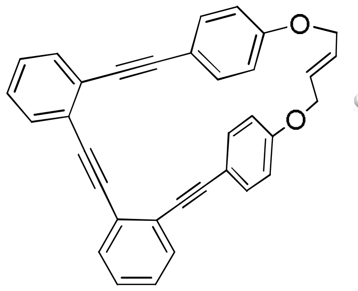

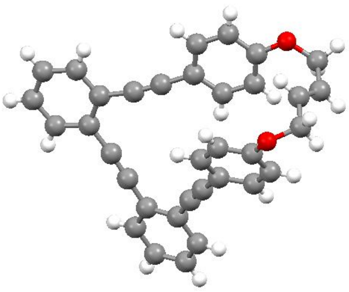

Compound 12: According to GP5: 6:1 mixture of *E:Z* isomers. ***E* stereoisomer:** Colourless oil. ^{1}H NMR (500 MHz, $CDCl_3$) δ 7.64-7.60 (m, 2H), 7.57-7.53 (m, 2H), 7.35–7.32 (m, 4H), 7.03-6.97 (m, 4H), 6.77-6.75 (m, 2H), 6.64–6.63 (m, 2H), 5.73-7.72 (m, 2H), 4.63-4.62 (m, 4H); ^{13}C NMR (125 MHz, $CDCl_3$) δ 156.5 (C), 132.1(CH), 132.0 (CH), 129.6 (CH), 128.7 (CH), 128.2 (CH), 127.9 (CH), 126.4 (CH), 126.1 (C), 125.9 (C), 124.2 (C), 118.9 (CH), 117.2 (CH), 94.3 (C), 92.3 (C), 88.1 (C), 67.4 (CH_2). HRMS-(ES): $(M+Na)^+$m/z calcd for $C_{34}H_{22}O_2Na$, 485.1512; found 485.1528.

***Z* stereoisomer:** Colourless oil. ^{1}H NMR (500 MHz, $CDCl_3$) δ 7.61-7.59 (m, 2H), 7.55–7.52 (m, 2H), 7.33-7.30 (m, 4H), 7.12–7.06 (m, 4H), 6.79-6.75 (m, 4H), 5.93-5.91 (m, 2H), 4.62 (d, *J* = 4.0 Hz, 4H); ^{13}C NMR(125 MHz, $CDCl_3$) δ 158.2 (C), 132.6 (CH), 132.4 (CH), 129.1 (CH), 129.0 (CH), 128.2 (CH), 128.0 (CH), 125.8 (C), 125.7 (C), 125.6 (CH), 124.4 (C), 119.0 (CH), 115.9 (CH), 93.5 (C), 92.0 (C), 88.0 (C), 65.6 (CH_2). HRMS-(ES): $(M+Na)^+$m/z calcd for $C_{34}H_{22}O_2Na$, 485.1512; found 485.1528.

Compound 14: According to GP1: Yellowish oil. ^{1}H NMR (300 MHz, $CDCl_3$) δ 7.64 (dd, *J* = 8.1, 1.3 Hz, 1H), 7.57 (dd, *J* = 7.7, 1.8 Hz, 1H), 7.35–7.27 (m, 1H), 7.24-7.15 (m, 2H), 7.12-7.05 (m, 2H), 6.88 (ddd, *J* = 8.0, 2.7, 1.3 Hz, 1H), 5.08 (s, 1H, OH); ^{13}C NMR (75 MHz, $CDCl_3$) δ 155.5 (C), 133.4 (CH), 132.6 (CH), 129.8 (CH), 129.6 (CH), 127.2 (CH), 125.8 (C), 125.3 (C), 124.5 (CH), 124.2 (C), 118.4 (CH), 116.3 (CH) 94.6 (C), 88.3 (C); HRMS-(ES): $(M)^+$m/z calcd for $C_{14}H_9OBr$, 271.9837; found 271.9836.

Compound 15: According to GP1: Yellowish oil. ^{1}H NMR (300 MHz, $CDCl_3$) δ 7.52 (d, *J* = 8.2, 1H), 7.46 (d, *J* = 8.2 Hz, 1H), 7.43 (d, *J* = 8.6 Hz, 2H), 7.20 (t, *J* = 7.6, 1H), 7.07 (t, *J* = 7.6, 1H), 6.75 (d, *J* = 8.6 Hz, 2H), 5.02 (bs, OH); ^{13}C NMR (75 MHz, $CDCl_3$) δ 156.0 (C), 133.6 (CH), 133.1 (CH), 132.5 (CH), 129.2 (CH), 127.1 (CH), 125.7 (C), 125.6 (C), 115. (CH), 115.4 (C), 94.0 (C), 87.0 (C); HRMS-(ES): $(M)^+$m/z calcd for $C_{14}H_9OBr$, 271.9837; found 271.9835.

TMS-16: According to GP2: Yellowish oil. ^{1}H NMR (300 MHz, $CDCl_3$) δ 7.46–7.35 (m, 4H), 7.22-7.18 (m, 2H), 6.75 (d, *J* = 8.6 Hz, 2H), 0.20 (s, 9H); ^{13}C NMR (75 MHz, $CDCl_3$)

δ156.1 (C), 133.5 (CH), 132.4 (CH), 131.6 (CH), 128.4 (CH), 127.7 (CH), 126.5 (C), 125.4 (C), 115.6 (CH), 103.8 (C), 98.6 (C), 93.6 (C), 87.0 (C), 0.2 (CH_3); HRMS-(ES): $(M)^+$m/z calcd for $C_{19}H_{18}OSi$, 290.1127; found 290.1134.

Compound 16: According to GP3: Yellowish oil. ^1H NMR (300 MHz, $CDCl_3$) δ 7.48–7.41 (m, 2H), 7.38 (d, *J* = 8.6 Hz, 2H), 7.27-7.13 (m, 2H), 6.75 (d, *J* = 8.6 Hz, 2H), 3.31 (s, 1H)4.91 (s, 1H, OH); ^{13}C NMR (75 MHz, $CDCl_3$) δ 156.0 (C), 133.6 (CH), 132.6 (CH), 131.7 (CH), 128.7 (CH), 127.7 (CH), 126.6 (C), 124.4 (C), 115.7 (CH), 115.4 (C), 93.7 (C), 86.8 (C), 82.5 (C), 81.2 (CH_3); HRMS-(ES): $(M)^+$m/z calcd for $C_{16}H_{10}O$, 218.0732; found 218.0738.

Compound 17: According to GP2: Colourlessoil. ^1H NMR (300 MHz, $(CD_3)_2CO$) δ 7.67–7.52 (m, 5H), 7.44–7.32 (m, 6H), 7.17 (t, *J* = 8.0 Hz, 1H), 7.08-7.01 (m, 2H), 6.82 (d, *J* = 8.6 Hz, 2H);^{13}C NMR (75 MHz, $CDCl_3$) δ 155.9 (C), 155.2 (C), 133.5 (CH), 132.2 (CH), 132.0 (CH), 131.7 (CH), 129.6 (CH), 128.3 (CH), 128.3 (CH), 128.2 (CH), 127.8 (CH), 126.1 (C), 125.9 (C), 125.6 (C), 125.5 (C), 124.6 (C), 124.4 (CH), 118.5 (CH), 116.0 (CH), 115.7 (CH), 115.6 (C), 115.5 (C), 94.1 (C), 93.5 (C), 92.6 (C), 92.2 (C), 88.4 (C), 87.1 (C); HRMS-(ES): $(M)^+$m/z calcd for $C_{30}H_{18}O_2$, 410.1307; found 410.1308.

Allyl-17: Accordingto GP4: Colourlessoil. ^1H NMR (300 MHz, $CDCl_3$) δ 7.63–7.47 (m, 5H), 7.44 (d, *J* = 8.7 Hz, 2H), 7.32–7.21 (m, 4H), 7.17-7.05 (m, 2H), 6.84 (d, *J* = 8.3 Hz, 1H), 6.78 (d, *J* = 8.6 Hz, 2H), 6.08-5.89 (m, 2H), 5.43-5.29 (m, 2H), 5.28–5.17 (m, 2H), 4.45 (d, *J* = 5.2 Hz, 2H), 4.38 (d, *J* = 5.2 Hz, 2H); ^{13}C NMR (75 MHz, $CDCl_3$) δ 158.7 (C), 158.3 (C), 133.2 (CH), 133.0 (CH), 132.9 (CH), 132.1 (CH), 132.0 (CH), 131.8 (CH), 131.6 (CH), 129.3 (CH), 128.2 (CH), 128.1 (CH), 127.6 (CH), 126.2 (C), 126.0 (C), 125.8 (C), 125.6 (C), 124.4 (C), 124.2 (CH), 117.8 (CH_2), 117.6 (CH_2), 117.0 (CH), 116.2 (CH), 115.5 (CH), 114.8 (C), 114.7 (C), 94.1 (C), 93.9 (C), 92.5 (C), 92.2 (C), 88.2 (C), 87.2 (C), 68.7 (CH_2), 68.6 (CH_2); HRMS-(ES): $(M)^+$m/z calcd for $C_{36}H_{26}O_2$, 490.1933;found 490.1941.

Compound 18: According to GP5: White solid. ^1H NMR (300 MHz, $CDCl_3$) δ 7.72–7.52 (m, 4H), 7.41-7.27 (m, 4H), 7.19 (d, *J* = 8.6 Hz, 2H), 7.09 (d, *J* = 7.6 Hz, 1H), 7.00–6.87 (m,

1H), 6.75-6.62 (m, 2H), 6.51 (d, J = 8.6 Hz, 2H), 5.81-5.61 (m, 2H), 4.76–4.54 (m, 4H); ^{13}C NMR (75 MHz, $CDCl_3$) δ 156.7 (C), 156.3 (C), 133.2 (CH), 132.9 (CH), 132.5 (CH), 132.4 (CH), 131.7 (CH), 130.2 (CH), 129.0 (CH), 128.3 (CH), 128.2 (CH), 128.0 (CH), 127.4 (CH), 126.2 (C), 125.7 (C), 125.5 (C), 125.4 (C), 124.0 (C), 118.1 (CH), 117.7 (CH), 115.7 (CH), 115.4 (C), 94.6 (C), 94.1 (C), 92.3 (C), 92.0 (C), 88.2 (C), 87.0 (C), 66.9 (CH_2), 66.8 (CH_2); HRMS-(ES): $(M)^+$m/z calcd for $C_{34}H_{22}O_2$,462.1620; found 462.1613.

***p,p*-1:** According to GP6: White solid. ^{1}H NMR(300 MHz, CD_2Cl_2) δ 7.58–7.55 (m, 2H), 7.53–7.50 (m, 2H), 7.28–7.24 (m, 4H), 7.16 (d, J = 8.8 Hz, 4H), 6.35 (d, J = 8.8 Hz, 4H), 5.30–5.17 (m, 2H), 4.22 (d, J = 8.5 Hz, 2H), 4.06–3.93 (m, 2H); ^{13}C NMR (125 MHz, CD_2Cl_2) δ 157.9 (C), 134.0 (CH), 133.8 (CH), 133.2 (CH), 128.8 (CH), 128.2 (CH), 125.7 (C), 125.2 (C), 115.9 (C), 114.9 (CH), 94.3 (C), 92.6 (C), 87.6 (C), 67.8 (CH), 67.21 (CH_2). HRMS-(ES): $(M+H)^+$m/z calcd for $C_{32}H_{25}O_4$, 497.1663;found 497.1693.

***m,m*-2:** According to GP6: white solid. ^{1}H NMR(500 MHz, $CDCl_3$) δ 7.61 (dd, J = 5.9, 2.9 Hz, 2H), 7.54–7.48 (m, 2H), 7.33-7.29 (m, 6H), 7.14 (d, J = 7.9 Hz, 2H), 7.04 (d, J = 7.9 Hz, 2H), 6.78 (dd, J = 8.2, 2.6 Hz, 2H), 6.28 (s, 2H), 4.21 (d, J = 8.8 Hz, 2H), 4.17–4.05 (m, 4H); ^{13}C NMR (125 MHz, $CDCl_3$) δ 157.5 (C), 132.4 (CH), 132.2 (CH), 129.1 (CH), 128.2 (CH), 128.0 (CH), 125.7 (C), 125.7 (C), 125.6 (C), 124.5 (C), 118.8 (CH), 113.1 (CH), 93.3 (C), 92.0 (C), 87.8 (C), 70.9 (CH), 67.9 (CH_2); HRMS-(ES): $(M+H)^+$m/z calcd for $C_{32}H_{25}O_4$, 497.1663; found 497.1703.

***p,m*-3:** According to GP6: white solid. ^{1}H NMR (300 MHz, $CDCl_3$) δ 7.79–7.69 (m, 2H), 7.68–7.58 (m, 2H), 7.50-7.35 (m, 4H), 7.24 (d, J = 8.6 Hz, 2H), 7.02 (t, J = 7.9 Hz, 1H), 6.97 (d, J = 7.5 Hz, 1H), 6.81-6.72 (m, 1H), 6.63 (d, J = 8.4 Hz, 2H), 6.58 (bs, 1H), 4.39 (dd, J = 11.2, 3.5 Hz, 1H), 4.28–4.01 (m, 5H), 2.88 (bs, 2H); ^{13}C NMR (150 MHz, CD_2Cl_2) δ 158.1 (C), 157.6 (C), 133.8 (CH), 133.5 (CH), 133.3 (CH), 133.1 (CH), 132.4 (CH), 129.7 (CH), 128.9 (CH), 128.8 (CH), 128.6 (CH), 128.2 (CH), 126.3 (C), 125.7 (C), 125.6 (C), 125.5 (C), 124.7 (C), 118.4 (CH), 116.3 (CH), 115.6 (CH), 115.4 (C), 94.2 (C), 93.8 (C), 92.6 (C), 92.3 (C), 88.4 (C), 87.2 (C), 69.7 (CH), 68.8 (CH), 68.7 (CH_2), 68.4 (CH_2); HRMS-(ES): $(M)^+$m/z calcd for $C_{34}H_{24}O_4$, 496.1675; found 496.1666.

Scheme S-4. Synthesis of camphanoyl derivatives 4.

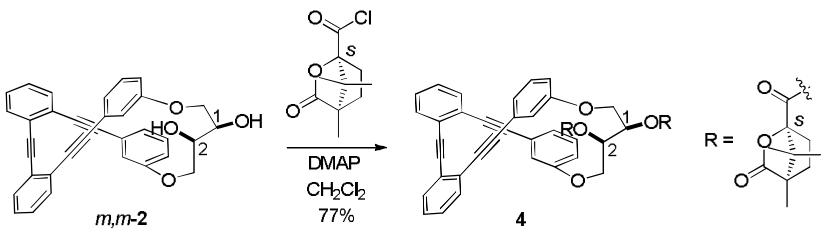

To a solution of diol *m*,*m*-**2** (112 mg, 0.23 mmol) in CH_2Cl_2 (10 mL),camphanic chloride (1.38 mmol) and DMAP (1.38 mmol) were added, and the mixture was stirred for 20 h at room temperature. Then, the solvent was removed. The residue was purified by flash chromatography in silica gel (EtOAc/hexane3:7) to yield the corresponding compounds**4a** (94mg, 54%) and **4b** (34mg, 23%).

Data of compound **4a**: White solid;^{1}H NMR(400 MHz, $CDCl_3$) δ 7.62 (dd, *J* = 6.1, 2.8 Hz, 2H), 7.51 (dd, *J* = 5.9, 3.1 Hz, 2H), 7.35–7.31 (m, 4H), 7.17 (t, *J* = 8.0 Hz, 2H), 7.03 (d, *J* = 7.6 Hz, 2H), 6.90 (dd, *J* = 8.2, 2.2 Hz, 2H), 6.25 (s, 2H), 5.68–5.64 (m, 2H), 4.30 (d, *J* = 10.7 Hz, 2H), 4.20 (dd, *J* = 11.4, 5.4 Hz, 2H), 2.55–2.46 (m, 2H), 2.06 (dt, *J* = 9.2, 5.2 Hz, 2H), 1.98–1.90 (m, 2H), 1.70 (ddd, *J* = 13.3, 9.3, 4.1 Hz, 2H), 1.12 (s, 6H), 1.07 (s, 6H), 1.00 (s, 6H). ^{13}C NMR (100 MHz, $CDCl_3$)δ 178.1 (C), 167.1 (C), 156.9 (C), 132.3 (CH), 132.1 (CH), 129.1 (CH), 128.3 (CH), 128.0 (CH), 126.3 (CH), 125.9 (C), 125.8 (C), 124.6 (C), 118.8 (CH), 112.4 (CH), 93.3 (C), 92.1 (C), 91.0 (C), 88.0 (C), 71.2 (CH), 64.8 (CH_2), 55.0 (C), 54.6 (C), 30.8 (CH_2), 29.9 (CH_2), 29.0 (C), 17.0 (CH_3), 16.9 (CH_3), 9.9 (CH_3).

Data ofcompound**4b**: White solid;^{1}H NMR(400 MHz, $CDCl_3$) δ 7.65–7.60 (m, 2H), 7.53–7.49 (m, 2H), 7.36–7.31 (m, 4H), 7.18 (t, *J* = 8.0 Hz, 2H), 7.05 (d, *J* = 7.6 Hz, 2H), 6.89 (dd, *J* = 8.2, 2.0 Hz, 2H), 6.26 (s, 2H), 5.70 (s, 2H), 4.30 (d, *J* = 11.2 Hz, 2H), 4.17 (dd, *J* = 10.7, 4.4 Hz, 2H), 2.49–2.39 (m, 2H), 2.11–1.93 (m, 4H), 1.73 (ddd, *J* = 13.2, 9.2, 3.9 Hz, 2H), 1.13 (s, 6H), 1.11 (s, 6H), 0.98 (s, 6H). ^{13}C NMR (125 MHz, $CDCl_3$)δ 177.7 (C), 166.8 (C), 156.8 (C), 132.4 (CH), 132.1 (CH), 129.2 (CH), 128.3 (CH), 128.1 (CH), 126.4 (CH), 125.9 (C), 125.8 (C), 124.8 (C), 118.7 (CH), 112.6 (CH), 93.2 (C), 92.1 (C), 90.9 (C), 88.1 (C), 71.1 (CH), 64.9 (CH_2), 54.9 (C), 54.5 (C), 31.0 (CH_2), 29.2 (CH_2), 17.1 (CH_3), 16.9 (CH_3), 9.8 (CH_3).HRMS-(ES): $(M)^+$m/z calcd for $C_{54}H_{48}O_{10}$, 856.3247; found 856.3243.

^{1}H and ^{13}C NMR ofnew compounds

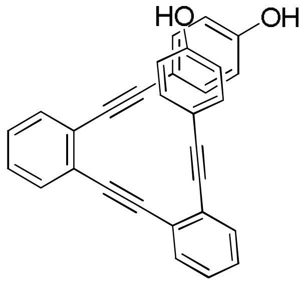

9

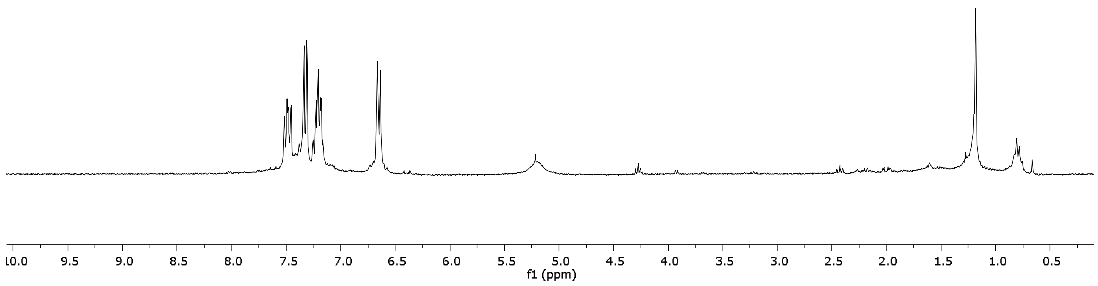

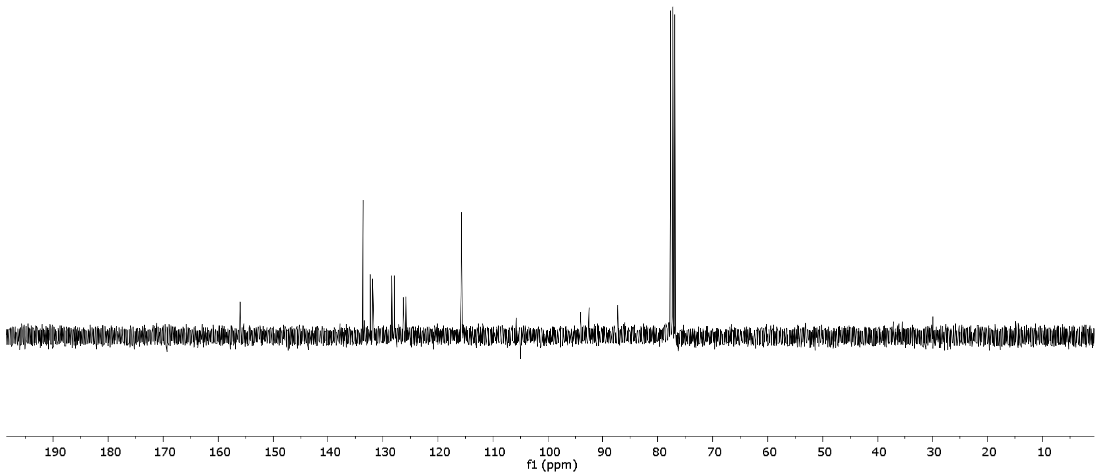

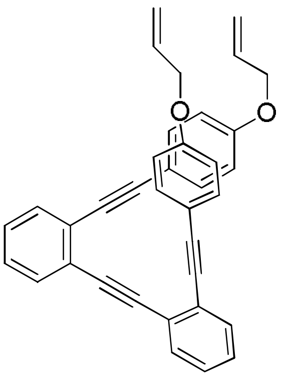

Allyl-9

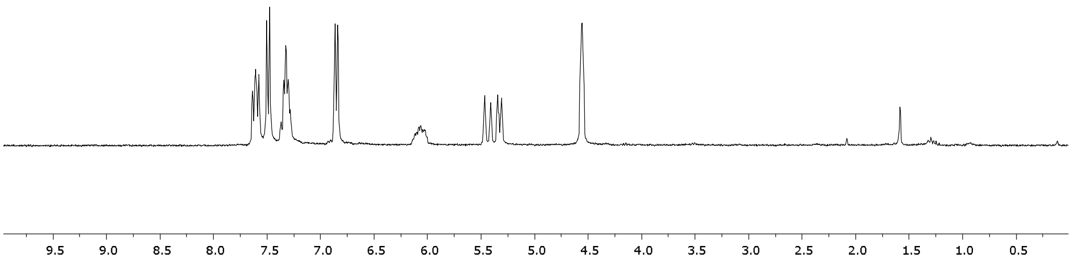
9.5
9.0
8.5
8.0
7.5
7.0
6.5
6.0
5.5
5.0
4.5
4.0
3.5
3.0
2.5
2.0
1.5
1.0
0.5

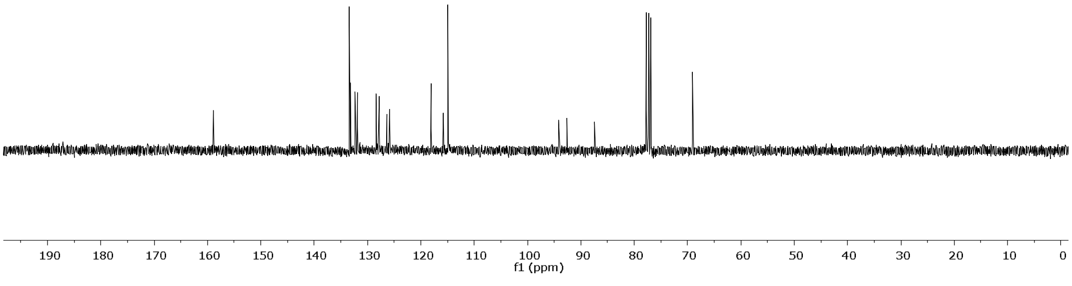
190
180
170
160
150
140
130
120
110
100
90
80
70
60
50
40
30
20
10
0
f1 (ppm)

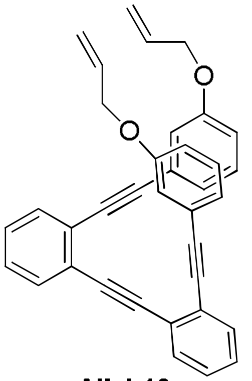

Allyl-10

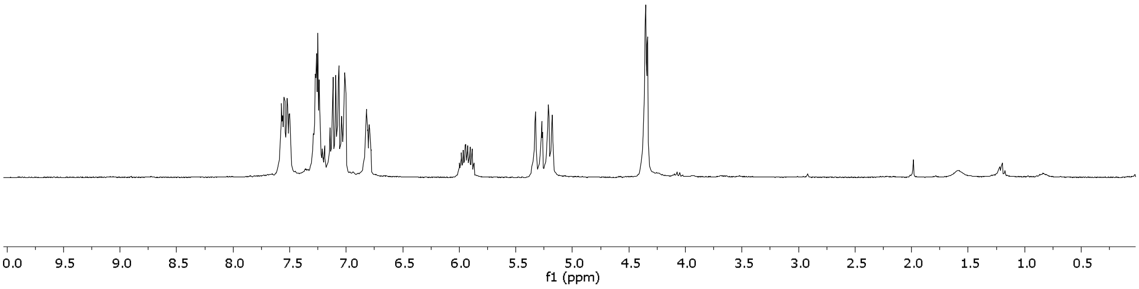
0.0 9.5 9.0 8.5 8.0 7.5 7.0 6.5 6.0 5.5 5.0 4.5 4.0 3.5 3.0 2.5 2.0 1.5 1.0 0.5
f1 (ppm)

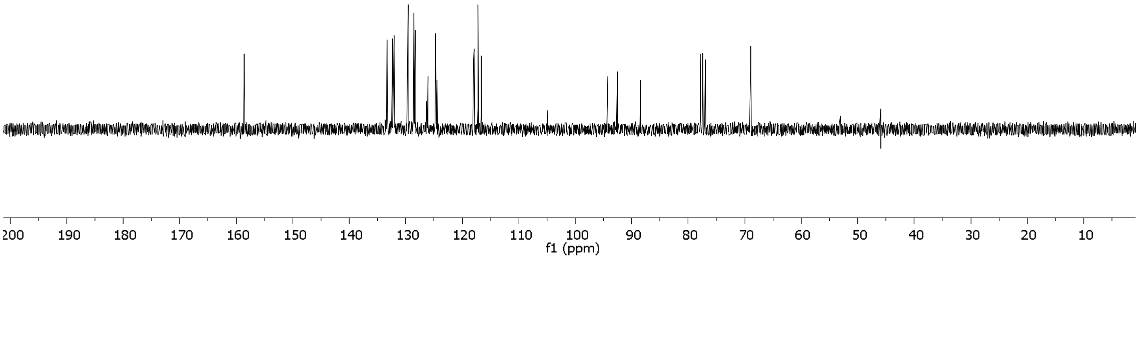
200 190 180 170 160 150 140 130 120 110 100 90 80 70 60 50 40 30 20 10
f1 (ppm)

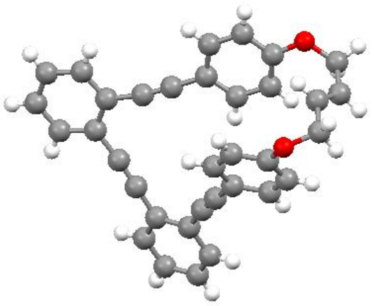

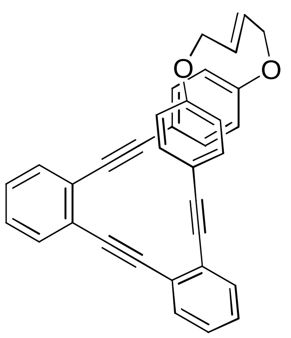
O
O

11

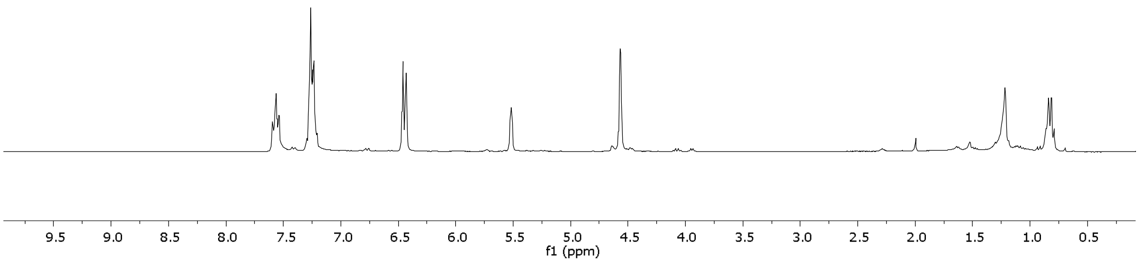
9.5 9.0 8.5 8.0 7.5 7.0 6.5 6.0 5.5 5.0 4.5 4.0 3.5 3.0 2.5 2.0 1.5 1.0 0.5
f1 (ppm)

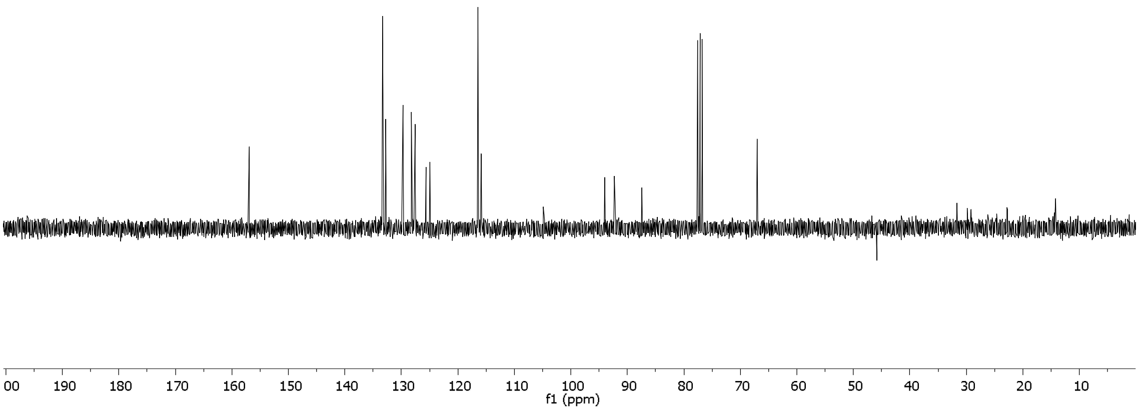
00 190 180 170 160 150 140 130 120 110 100 90 80 70 60 50 40 30 20 10
f1 (ppm)

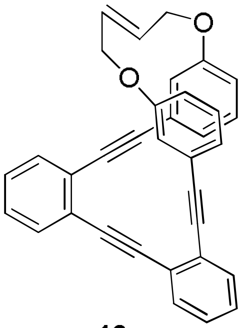
O
O

12

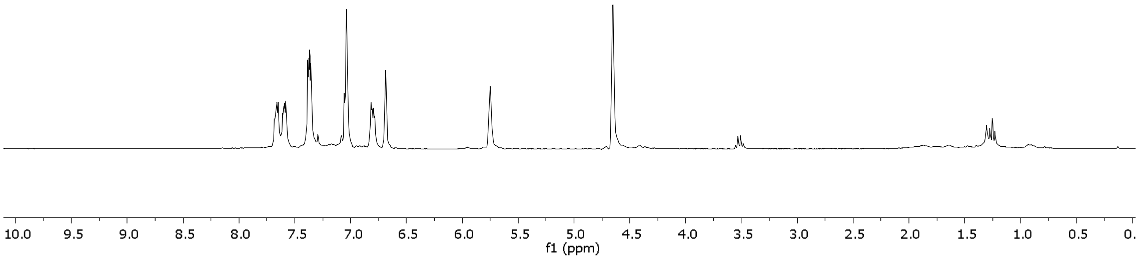
10.0 9.5 9.0 8.5 8.0 7.5 7.0 6.5 6.0 5.5 5.0 4.5 4.0 3.5 3.0 2.5 2.0 1.5 1.0 0.5
f1 (ppm)

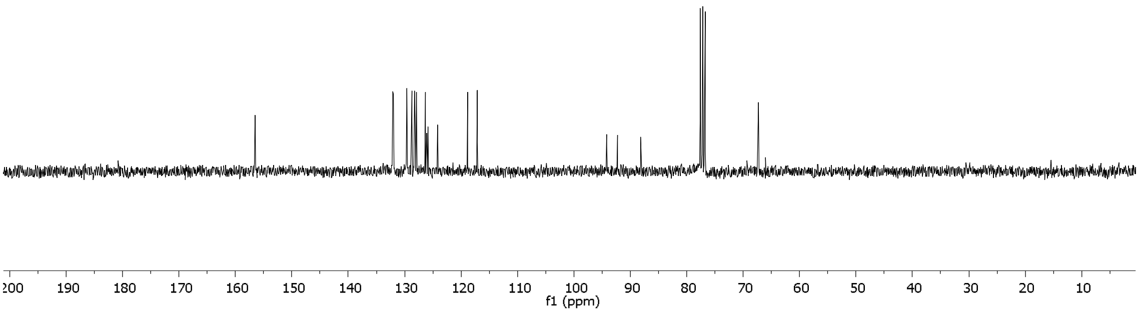
190 180 170 160 150 140 130 120 110 100 90 80 70 60 50 40 30 20 10
f1 (ppm)

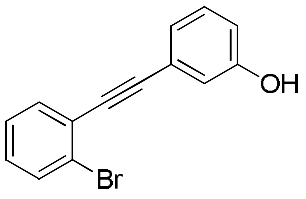

14

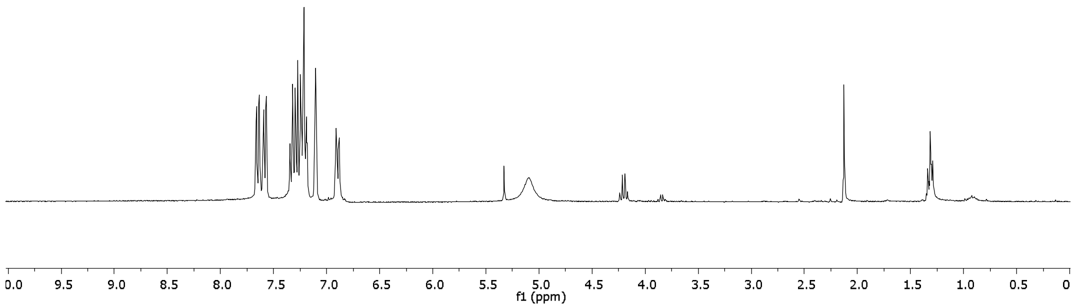

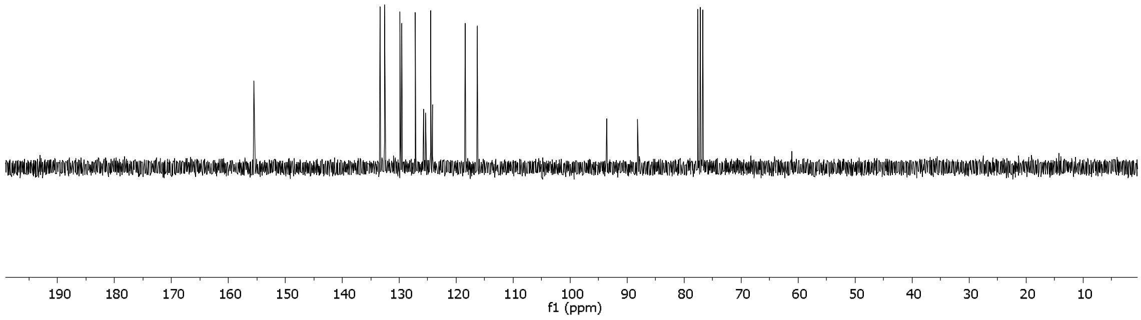

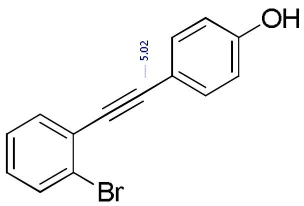
OH
Br

15

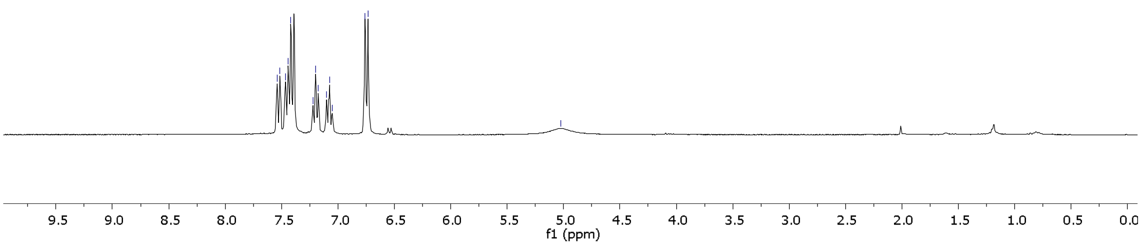
7.54
7.51
7.47
7.44
7.42
7.22
7.20
7.17
7.10
7.07
7.05
6.76
6.73
5.02
9.5 9.0 8.5 8.0 7.5 7.0 6.5 6.0 5.5 5.0 4.5 4.0 3.5 3.0 2.5 2.0 1.5 1.0 0.5 0.0
f1 (ppm)

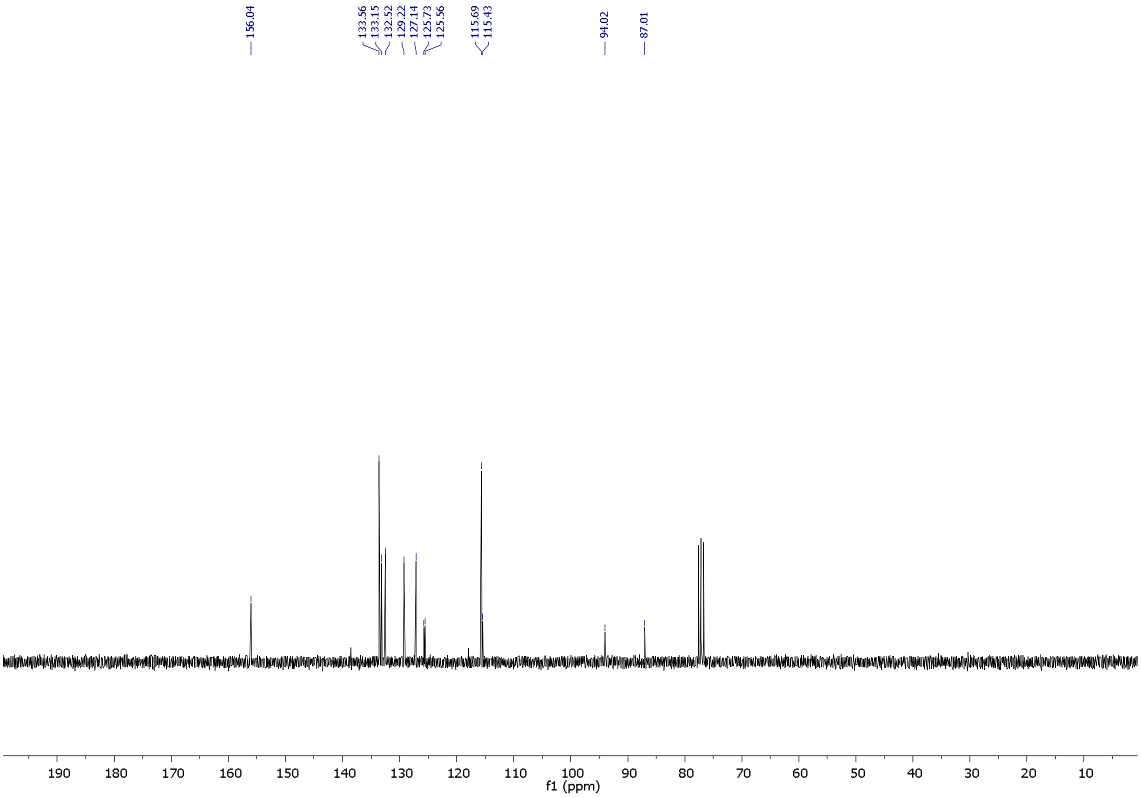
156.04
133.56
133.15
132.52
129.22
127.14
125.73
125.56
115.69
115.43
94.02
87.01
190 180 170 160 150 140 130 120 110 100 90 80 70 60 50 40 30 20 10
f1 (ppm)

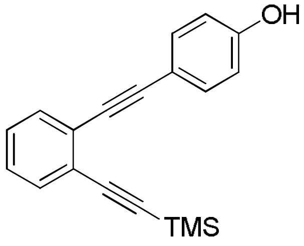
OH
TMS

16-TMS

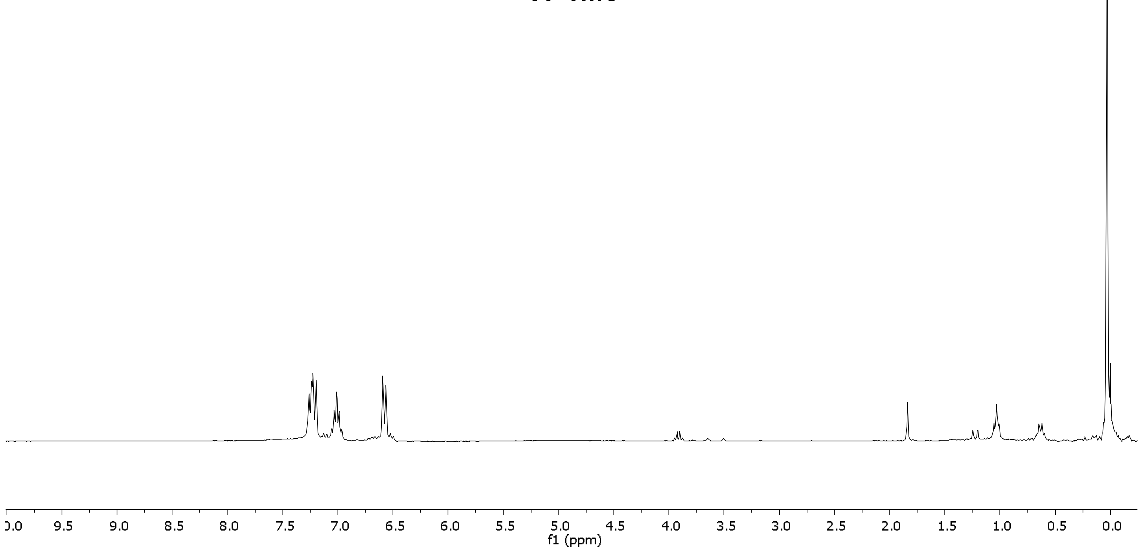
9.5 9.0 8.5 8.0 7.5 7.0 6.5 6.0 5.5 5.0 4.5 4.0 3.5 3.0 2.5 2.0 1.5 1.0 0.5 0.0
f1 (ppm)

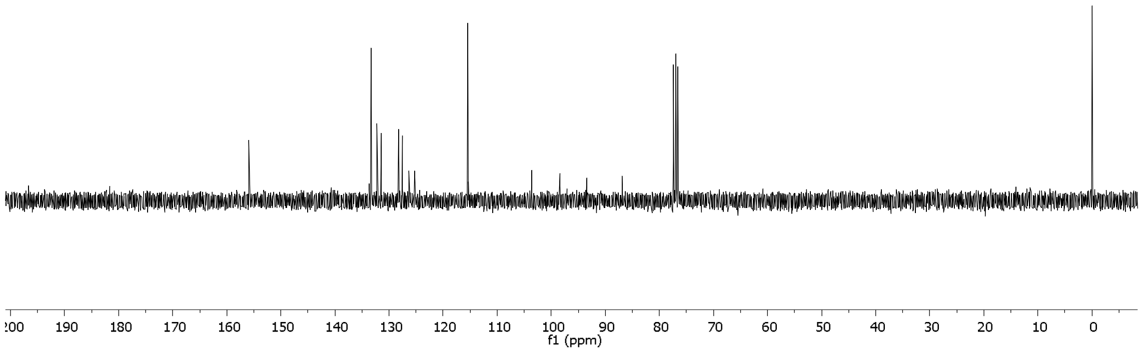
190 180 170 160 150 140 130 120 110 100 90 80 70 60 50 40 30 20 10 0
f1 (ppm)

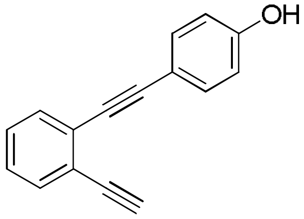
OH

16

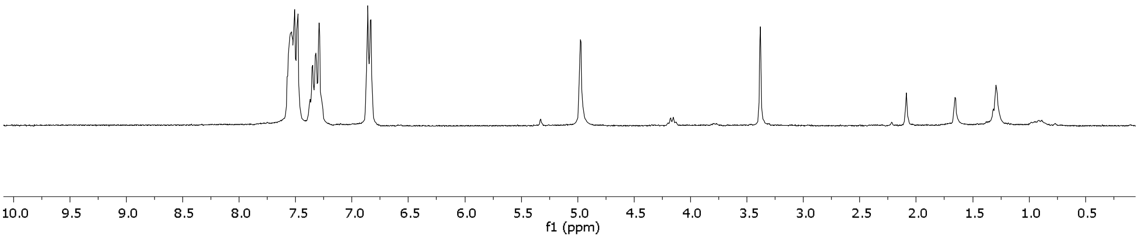
10.0
9.5
9.0
8.5
8.0
7.5
7.0
6.5
6.0
5.5
5.0
4.5
4.0
3.5
3.0
2.5
2.0
1.5
1.0
0.5
f1 (ppm)

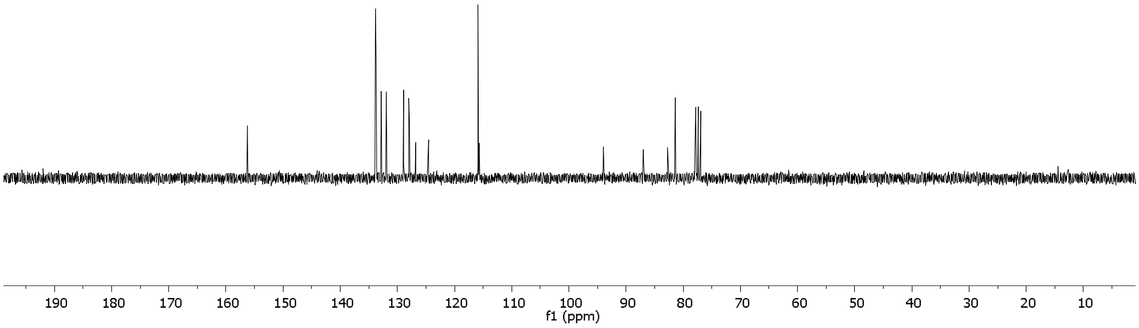
190
180
170
160
150
140
130
120
110
100
90
80
70
60
50
40
30
20
10
f1 (ppm)

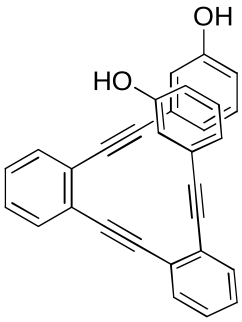

17

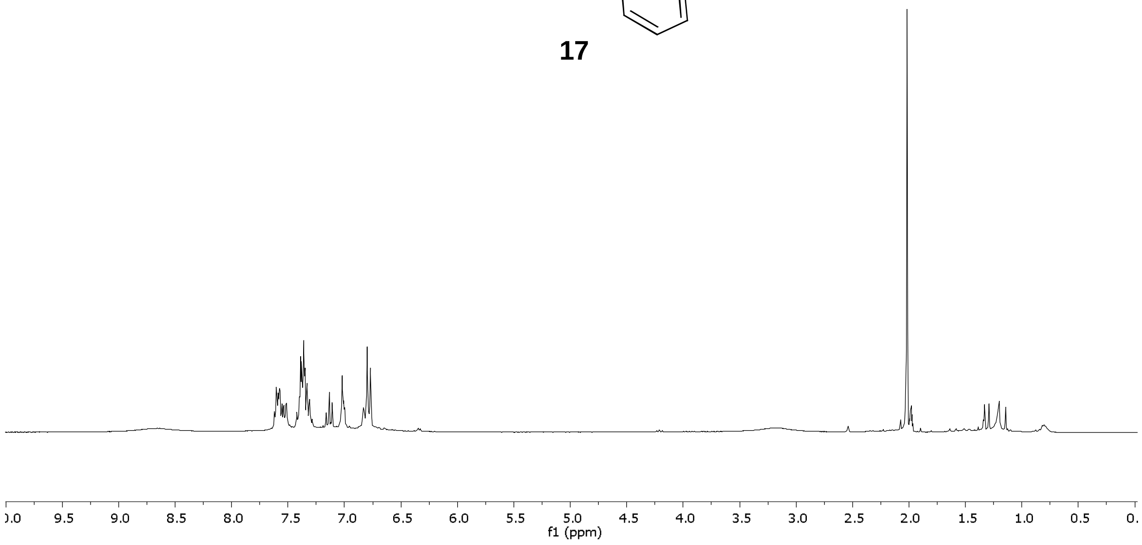

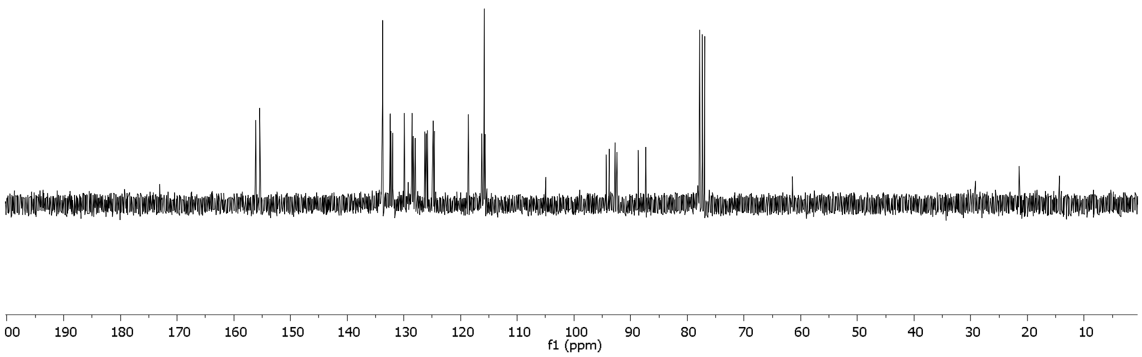

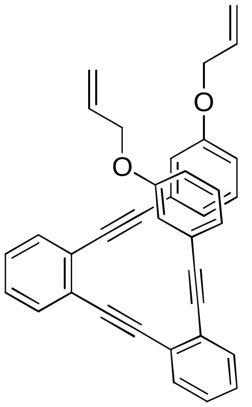
O
O

Allyl-17

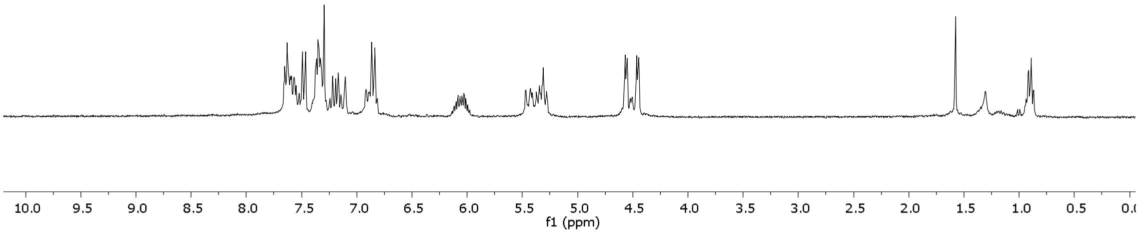
10.0 9.5 9.0 8.5 8.0 7.5 7.0 6.5 6.0 5.5 5.0 4.5 4.0 3.5 3.0 2.5 2.0 1.5 1.0 0.5
f1 (ppm)

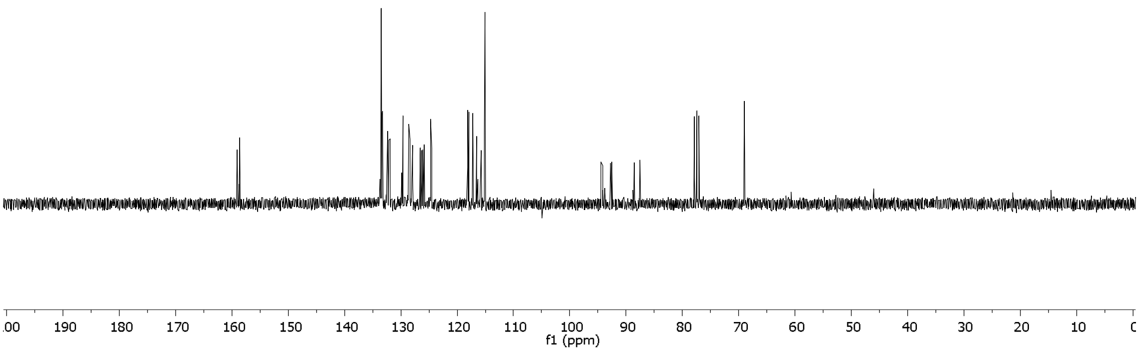
190 180 170 160 150 140 130 120 110 100 90 80 70 60 50 40 30 20 10
f1 (ppm)

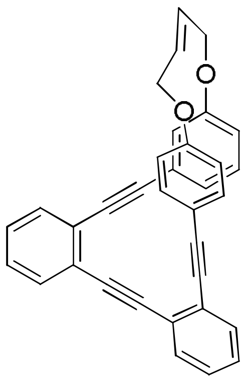

18

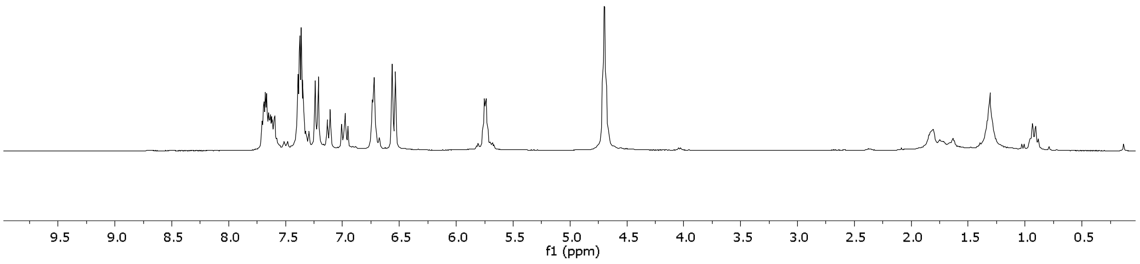
9.5 9.0 8.5 8.0 7.5 7.0 6.5 6.0 5.5 5.0 4.5 4.0 3.5 3.0 2.5 2.0 1.5 1.0 0.5
f1 (ppm)

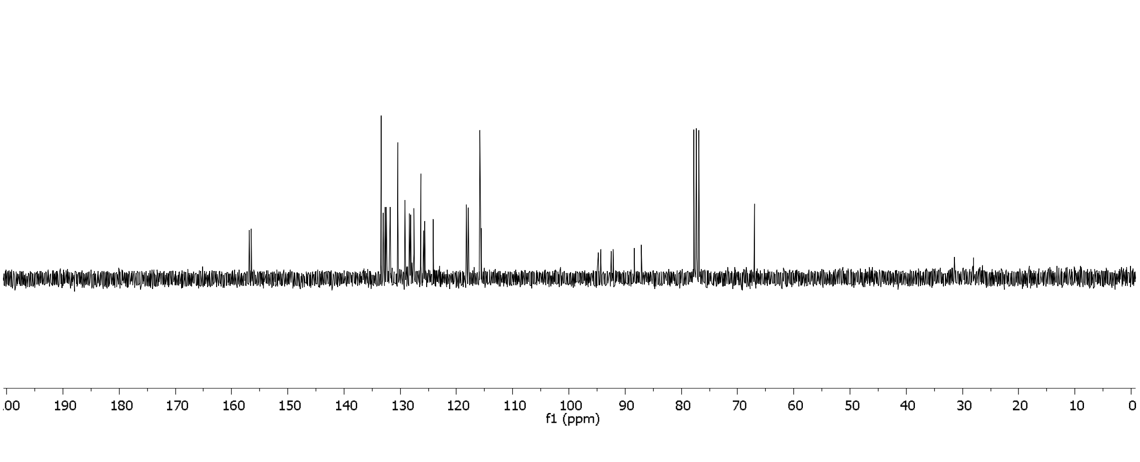
00 190 180 170 160 150 140 130 120 110 100 90 80 70 60 50 40 30 20 10 0
f1 (ppm)

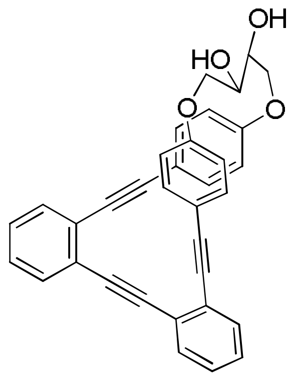

OH
HO
O
O

***p,p*-1**

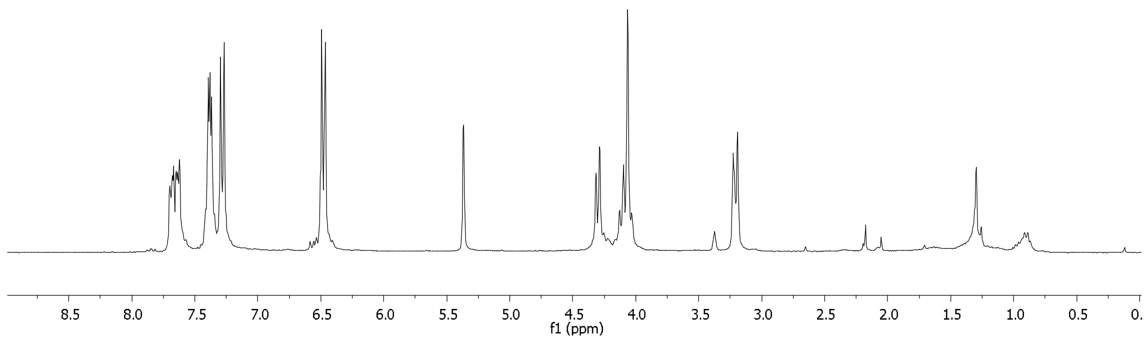

8.5
8.0
7.5
7.0
6.5
6.0
5.5
5.0
4.5
4.0
3.5
3.0
2.5
2.0
1.5
1.0
0.5
f1 (ppm)

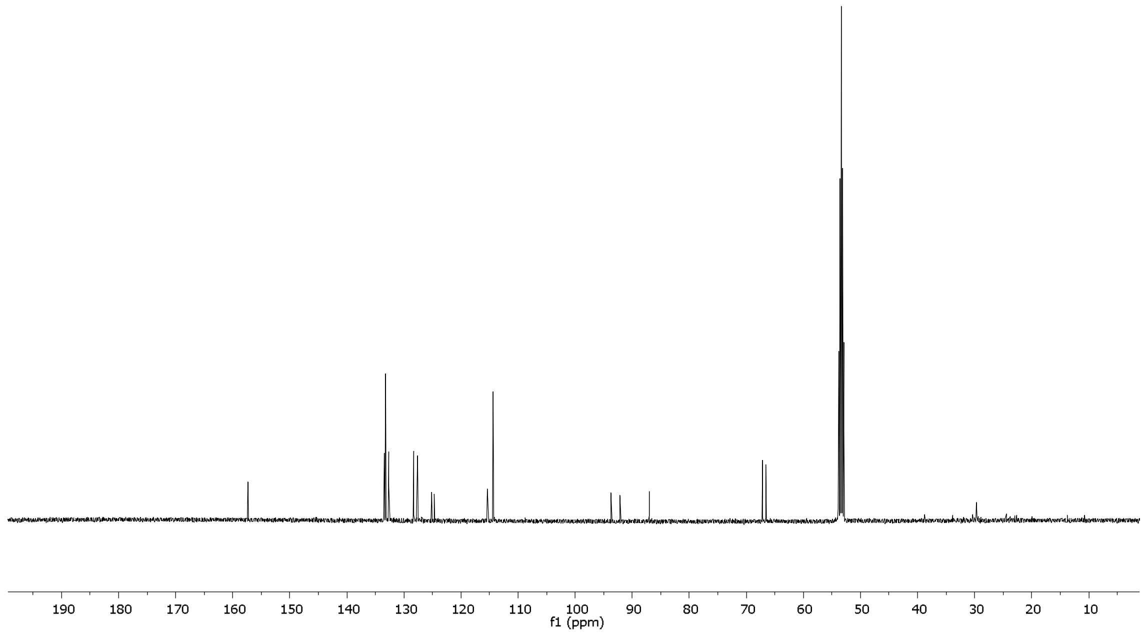

190
180
170
160
150
140
130
120
110
100
90
80
70
60
50
40
30
20
10
f1 (ppm)

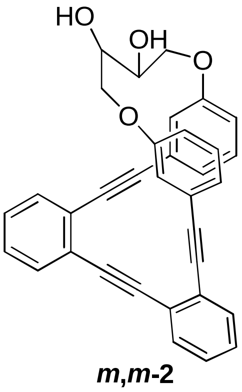
HO
OH
O
O

***m*,*m*-2**

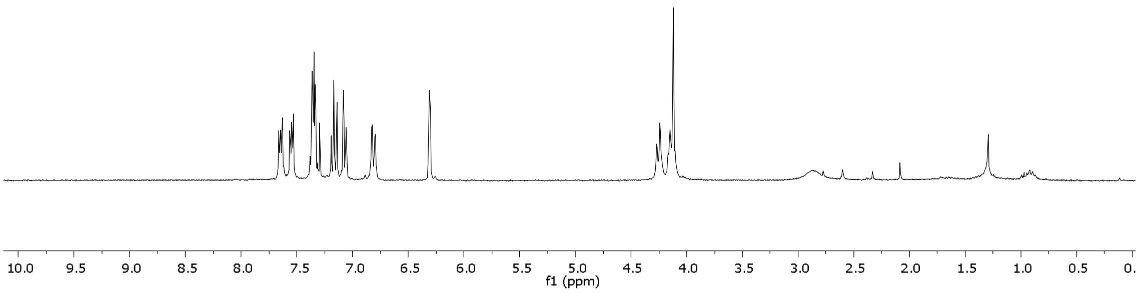
10.0 9.5 9.0 8.5 8.0 7.5 7.0 6.5 6.0 5.5 5.0 4.5 4.0 3.5 3.0 2.5 2.0 1.5 1.0 0.5
f1 (ppm)

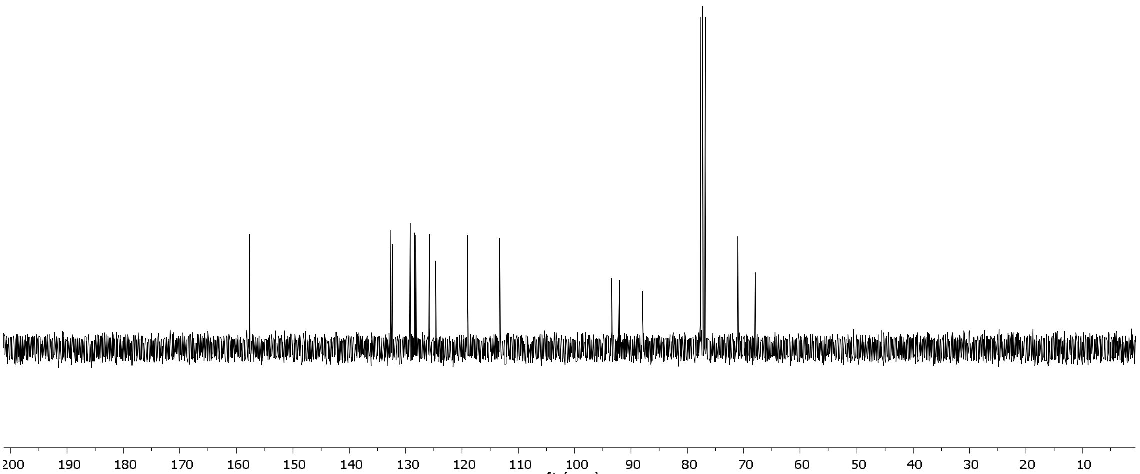
200 190 180 170 160 150 140 130 120 110 100 90 80 70 60 50 40 30 20 10
f1 (ppm)

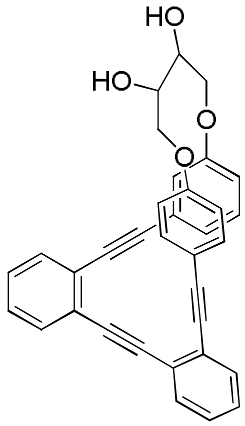

***p,m*-3**

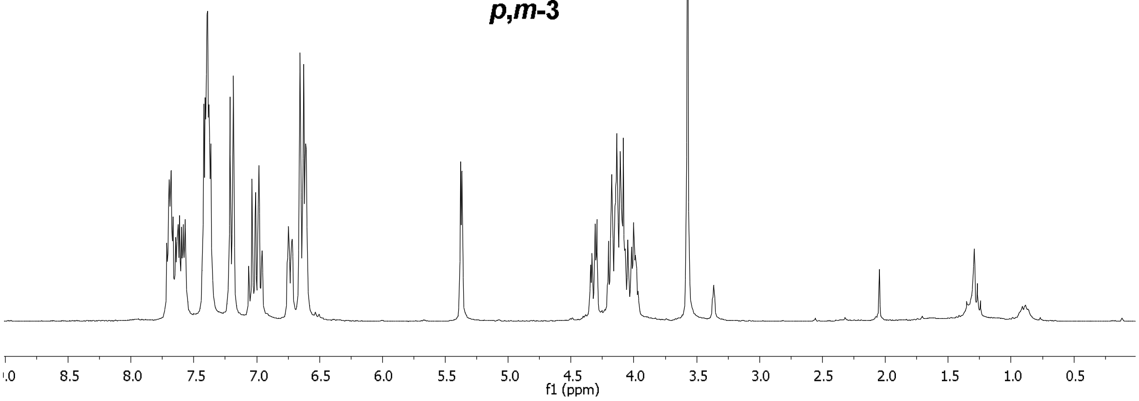

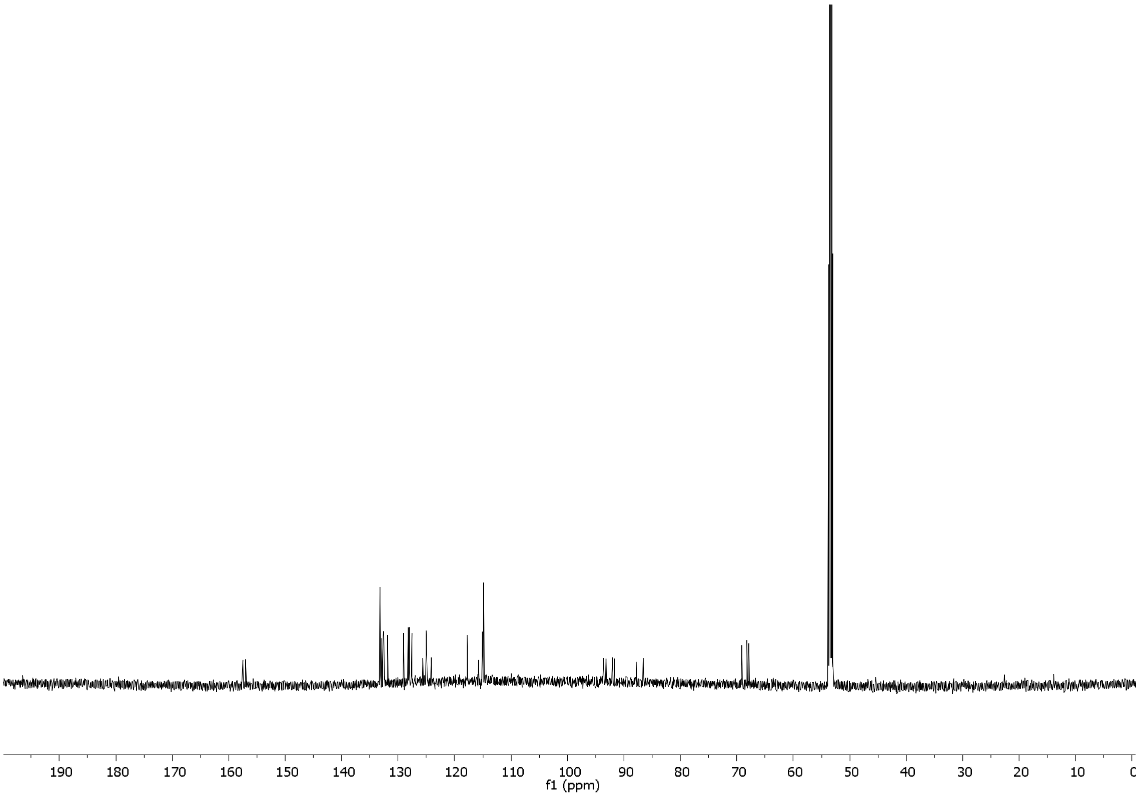

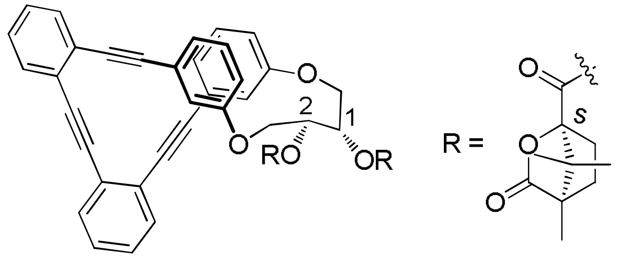

m,m-(*1´S,4´R,M,1S,2S*)-**4a**

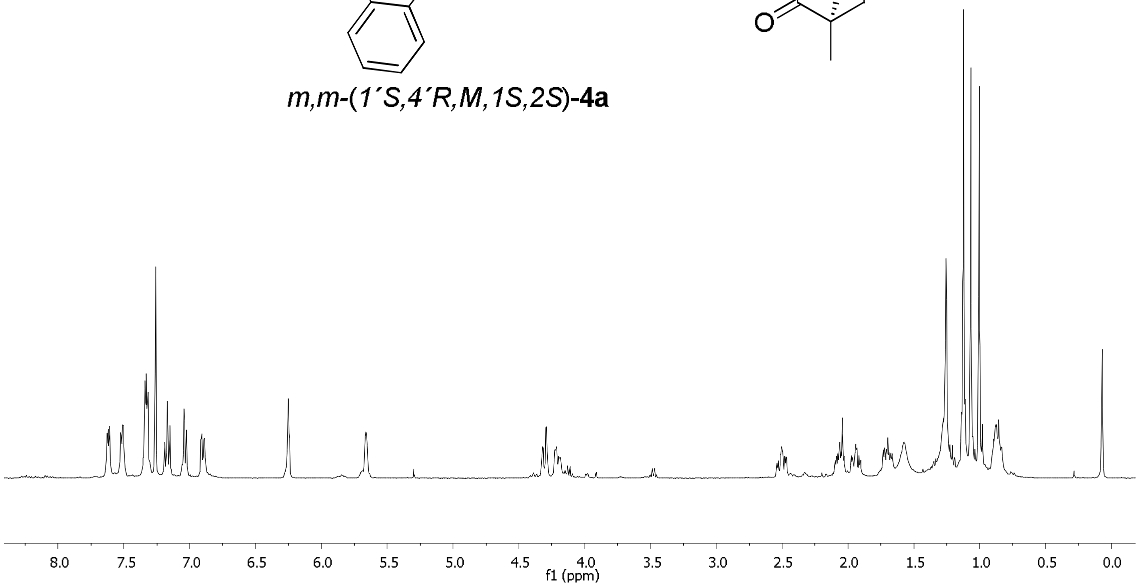

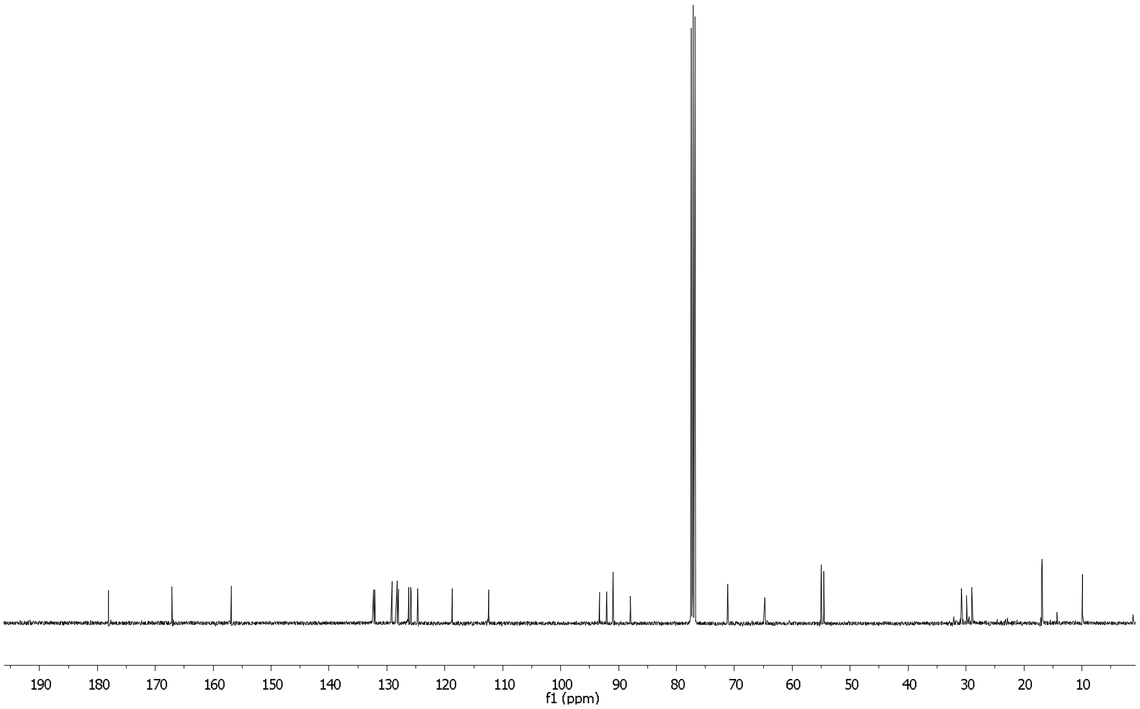

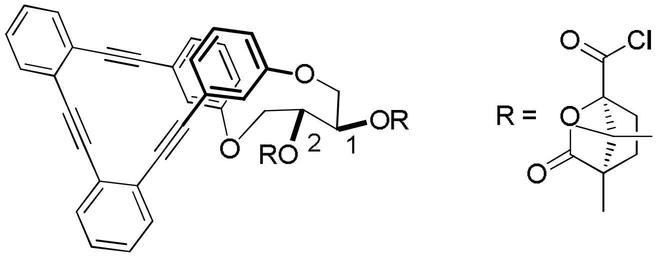

m,m-(*1´S,4´R,P,1R,2R*)-**4b**

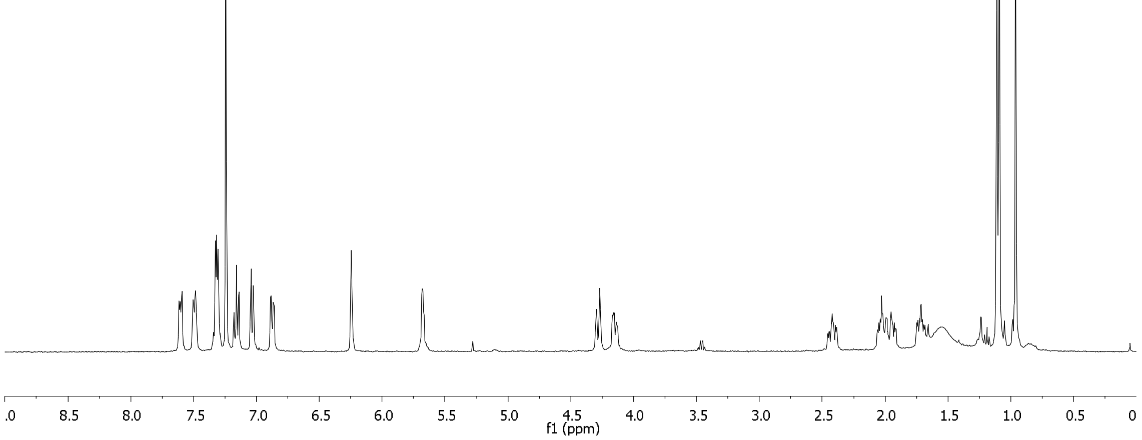

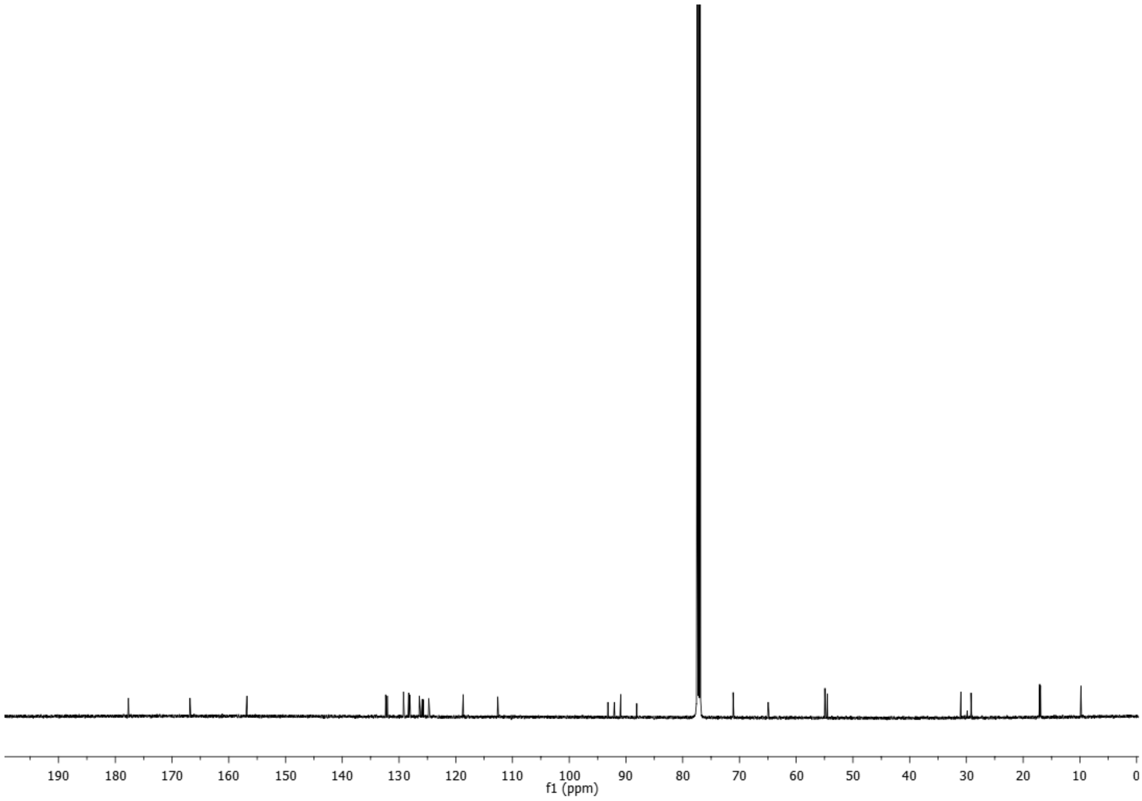

Low temperature 1H-NMR of compounds **1-3**

Diol p,p-1

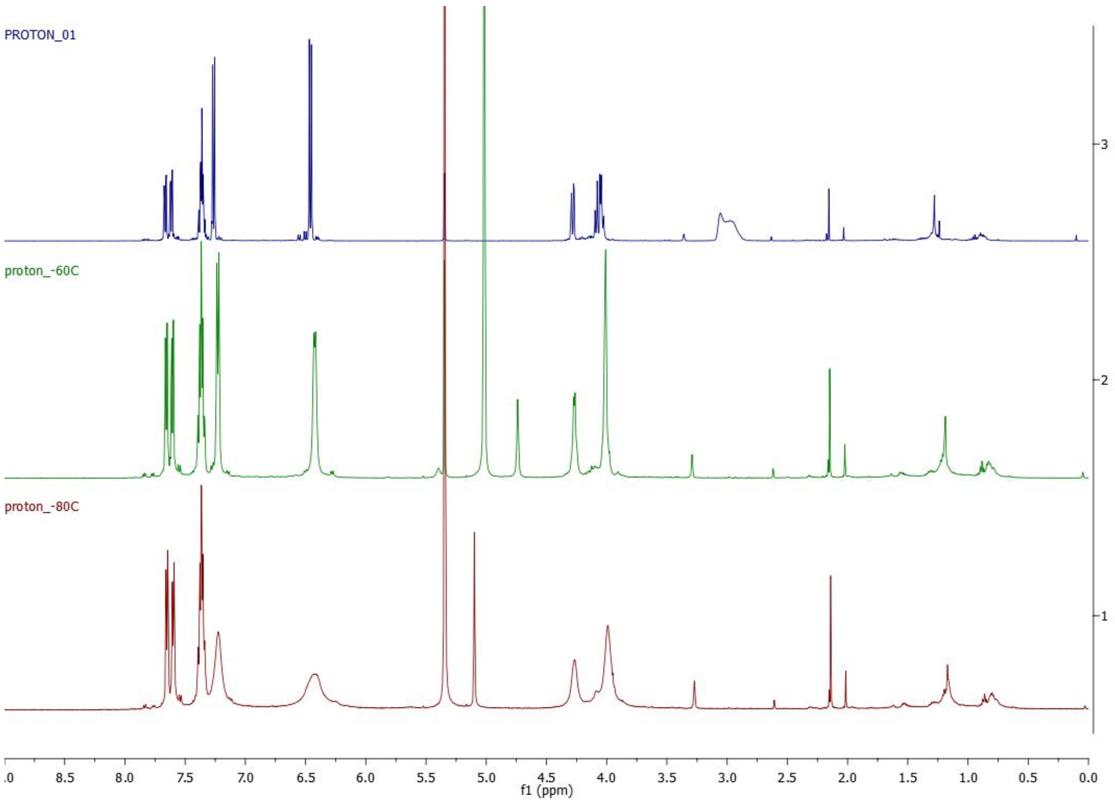

Diol m,m-2

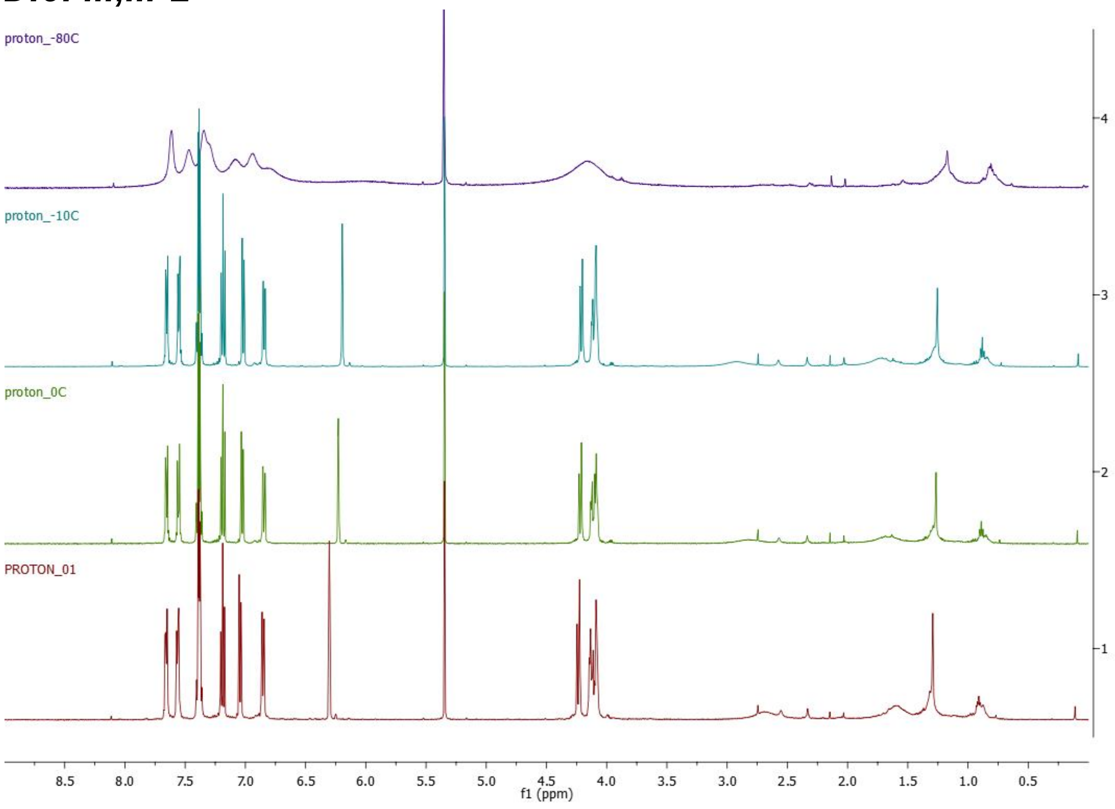

Diol m,p-3

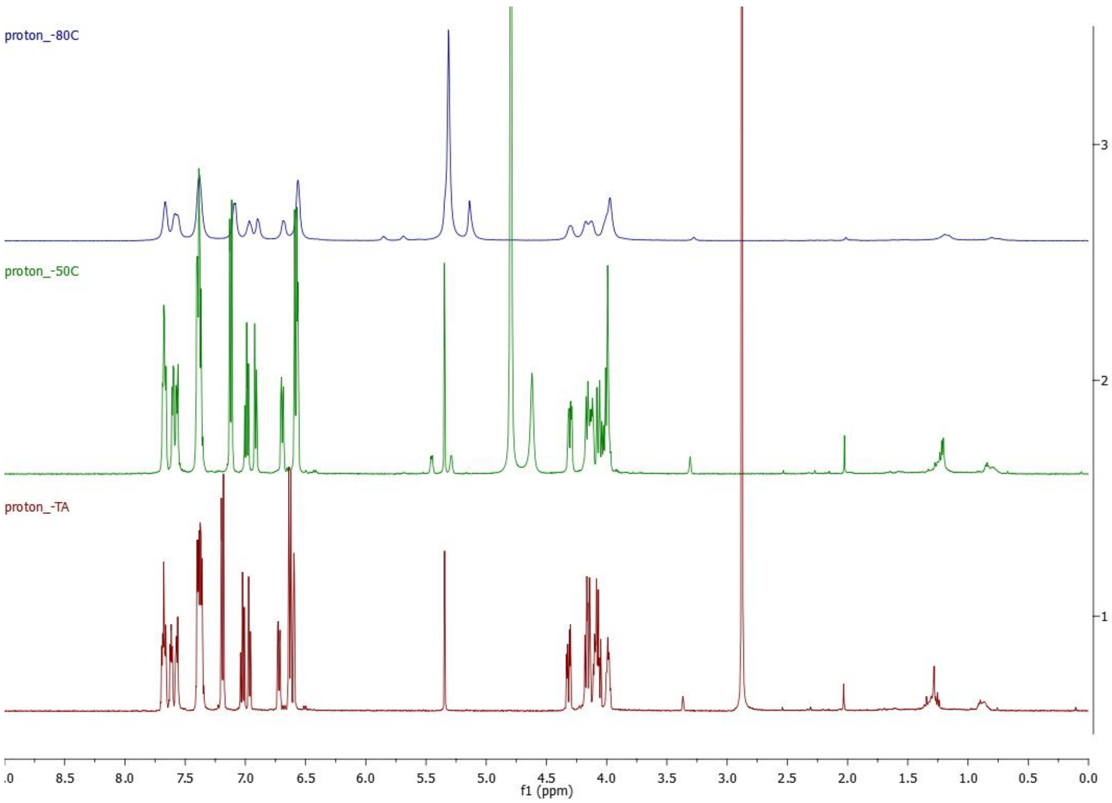

proton_-80C
proton_-50C
proton_-TA
3
2
1
.0
8.5
8.0
7.5
7.0
6.5
6.0
5.5
5.0
4.5
4.0
3.5
3.0
2.5
2.0
1.5
1.0
0.5
0.0
f1 (ppm)

PREPARATIVE CHIRAL HPLC RESOLUTION

Experimental Conditions

Thar SFC Investigator system (Thar Instruments) equipped with Variable Wavelength Detector (Gilson UV/VIS-151, Gilson, Middleton, WI, USA) was used for the separation. The mobile phase consisted of industrial carbon dioxide (99.995 % pure, Air Liquide) and HPLC grade methanol. One achiral HPLC column and one chiral column were utilized in this study.

The following conditions were used for the separation of compounds **1**—**3**:

Columns:-Chiral column: CHIRALCEL-OJ (5umx250mmx20mm)

Mobile Phase: A: Methanol; B: Carbon Dioxide (CO_2)

Gradient: A/B (40:60) in 40 min

Flow Rate: 15.0 mL/min

Temperature: 35 °C

Pressure: 150 bar

Detection: UV @ 270 nm

Injection Volume: 50ml

Single crystal X-ray analysis

Colourless block crystals of **4a** were grown from a tetrahydropyrane saturated solution under slow evaporation at room temperature. Measured crystals were prepared under inert conditions immersed in perfluoropolyether as protecting oil for manipulation. A suitable crystal was mounted on MiTeGen Micromounts TM, and this sample was used for data collection. Data were collected with Bruker D8 Venture diffractometer and processed with APEX2 program.[3] The structure was solved by direct methods,[4] which revealed the position of all non-hydrogen atoms. These atoms were refined on F2 by a full-matrix least-squares procedure using anisotropic displacement parameters.[4] All hydrogen atoms were located in difference Fourier maps and included as fixed contributions riding on attached atoms with isotropic thermal displacement parameters 1.2 (aromatic and methylene H atoms) or 1.5 (methyl H atoms) times those of the respective atom. Crystallographic data (excluding structure factors) for compound **4a** reported in this paper have been deposited with the Cambridge Crystallographic Data Center as supplementary publication no. CCDC 1443131.

Copies of the data can be obtained free of charge at http://www.ccdc.cam.ac.uk/products/csd/request/.

CD and CPL MEASUREMENTS

Experimental

Absorption and ECD measurements were conducted using a Jasco 815SE apparatus in a 2.0 mm path-length quartz cell.

Fluorescence and circularly polarized luminescence spectra of the same solutions used for CD spectra were recorded simultaneously using a homemade equipment.[5] The excitation radiation was brought to the cell from a Jasco FP8200 fluorimeter through an optical fiber containing water, a 90° scattering geometry was chosen, the incident radiation has been polarized parallel to the collection direction. Spectral response has been corrected using a reference lamp.

CD and CPL spectra

Table S1. g_{abs} and g_{lum} values of compounds **1-3** in CH_2Cl_2 and after neutralizing the Ag(I) action with acetonitrile (MeCN* (see text)).

Compound	Solvent	g_{abs}	g_{lum}
**1**	CH_2Cl_2	0.0096 (367nm)	0.011 (390nm)
	MeCN*	0.0095 (367nm)	0.0086 (390nm)
**2**	CH_2Cl_2	0.0019 (352nm)	0.0013 (383nm)
	MeCN*	0.0008 (352nm)	0.0013 (383nm)
**3**	CH_2Cl_2	0.0015 (354nm)	0.0009 (422nm: Ex.370)
	MeCN*	0.0013 (354nm)	0.0009 (422nm: Ex.370)
**4a**	CH_2Cl_2	0.0049 (350 nm)	0.0014 (427nm)
**4b**	CH_2Cl_2	0.0044 (350 nm)	0.0013 (427nm)

Figure S1. CD (top) and CPL (bottom) spectra of both enantiomers (**a** and **b**) ofcompound *p,p*-**1**, in CH_2Cl_2, with addition of $AgBF_4$, and with further addition of acetonitrile (MeCN). For CPL, excitation wavelength was set to 350 nm.

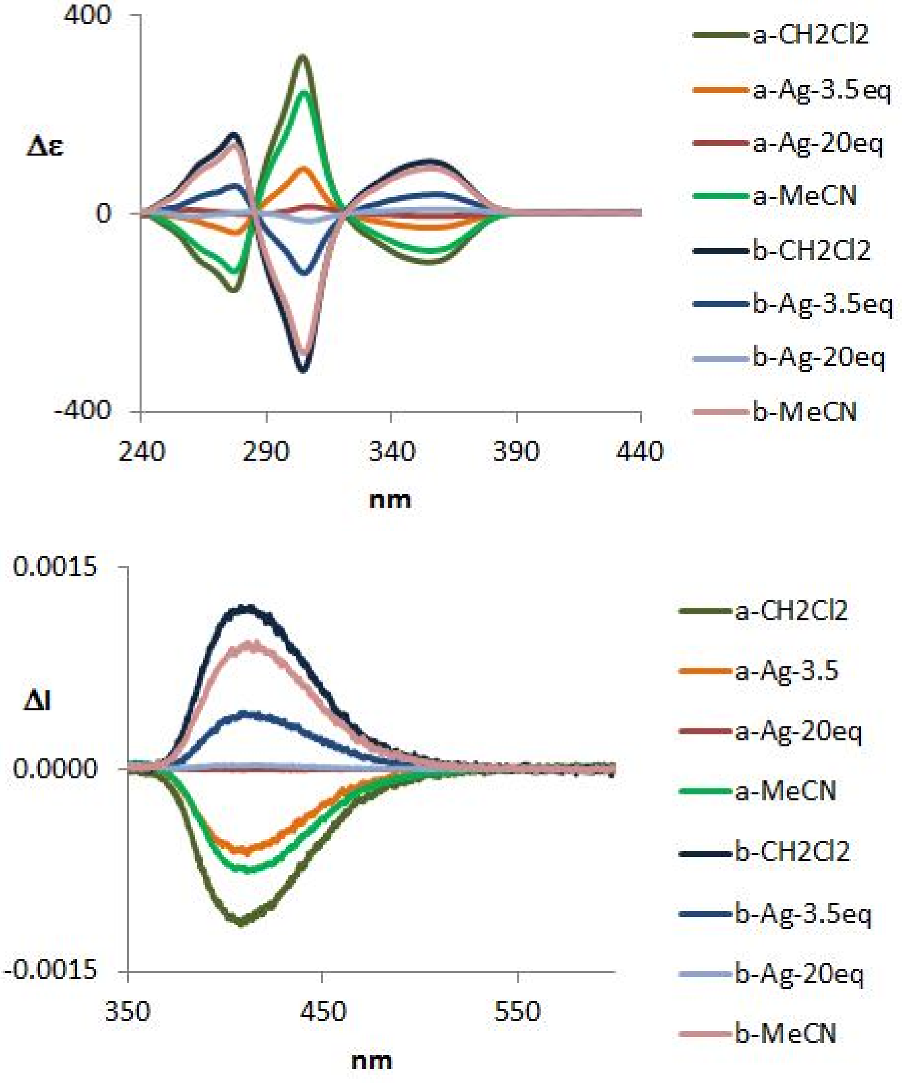

Figure S2. Absorption (left) and fluorescence (right) spectra ofboth enantiomers (**a** and **b**) of *p,p*-**1** in CH_2Cl_2, with addition of $AgBF_4$, and with further addition of acetonitrile (MeCN). Excitation wavelength 350nm. Same experimental condition as Figure S1.

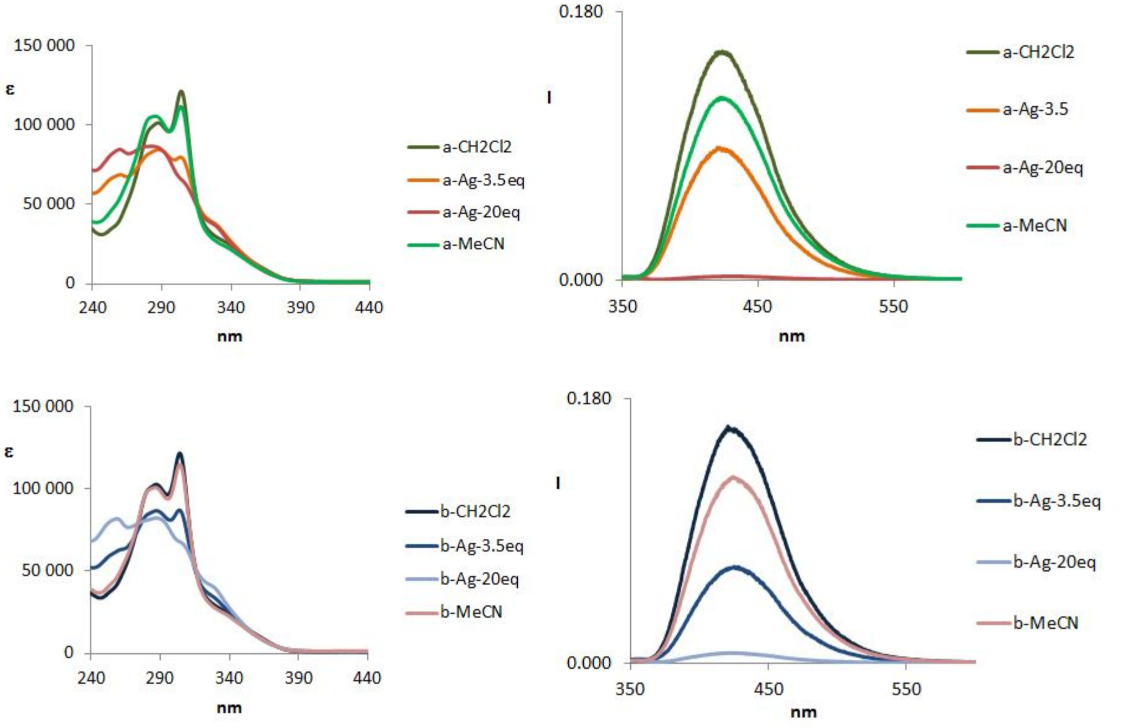

Figure S3. CD (top) and CPL (bottom) spectra of compound *m,m*-**2** in CH_2Cl_2, with addition of $AgBF_4$ (8 eq), and with further addition of acetonitrile (MeCN). For CPL, excitation wavelength 340 nm. During CPL scans changes in CD and CPL absorption and fluorescence are observed: measurement after 1 scan, 2 scans, 4, 6, and 8 scans are reported.

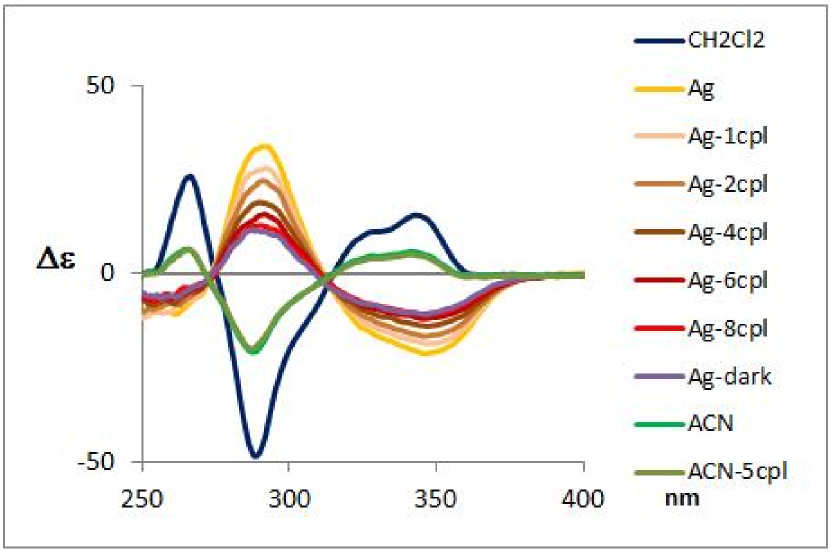

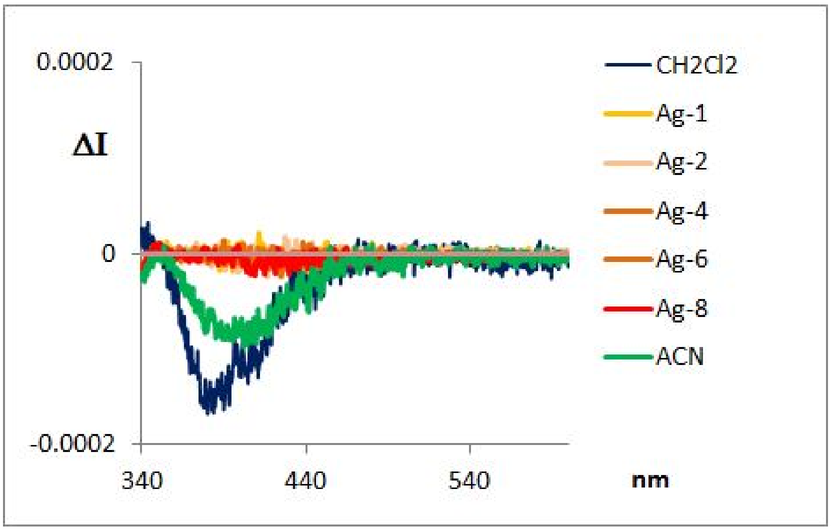

Figure S4. Absorption (top) and fluorescence (bottom) spectra of compound *m,m*-**2** in CH_2Cl_2, with addition of $AgBF_4$ (8 eq), and with further addition of acetonitrile (MeCN). Excitation wavelength 340 nm. During CPL scans changes in CD and CPL absorption and fluorescence are observed: measurement after 1 scan, 2 scans, 4, 6, and 8 scans are reported.

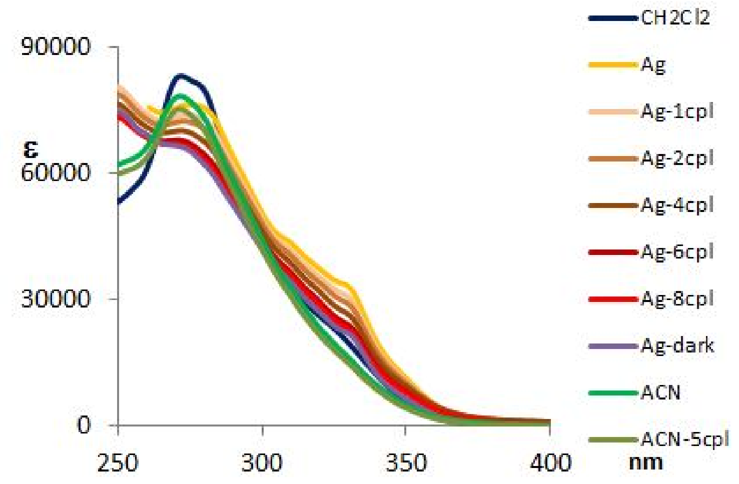

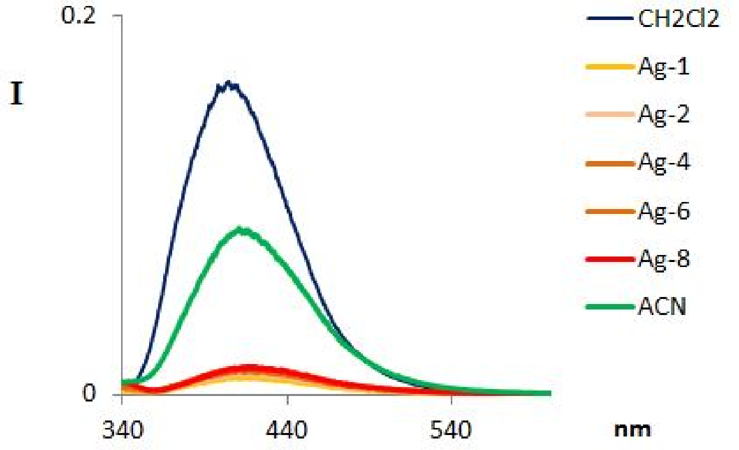

Figure S5. CD and absorption spectra ofboth enantiomers (**a** and **b**) of compound *p,m*-**3** in CH_2Cl_2, measured between 250 and 400nm.

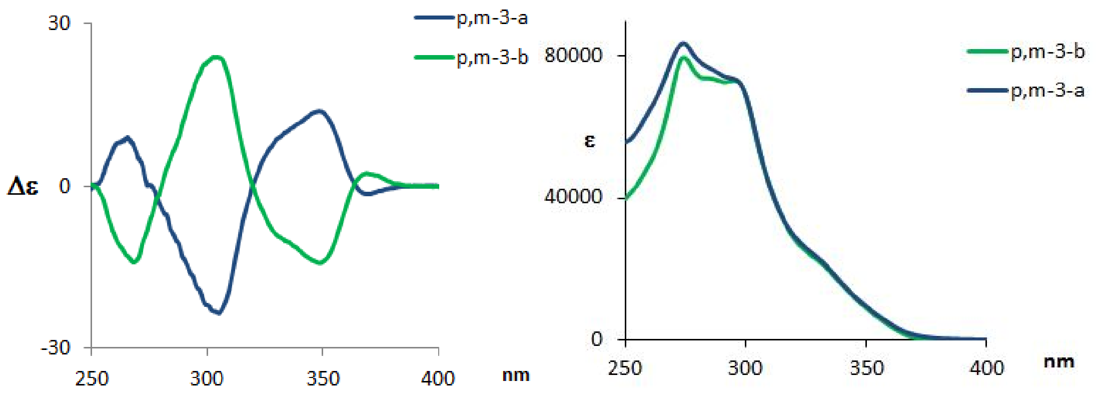

Figure S6. CD (top) and CPL (botton) spectra of both enantiomers (**a** and **b**) of compound *p,m*-**3**, in CH_2Cl_2, with addition of $AgBF_4$, and with further addition of scetonitrile. For CPL, excitation wavelength was set to 340 nm.

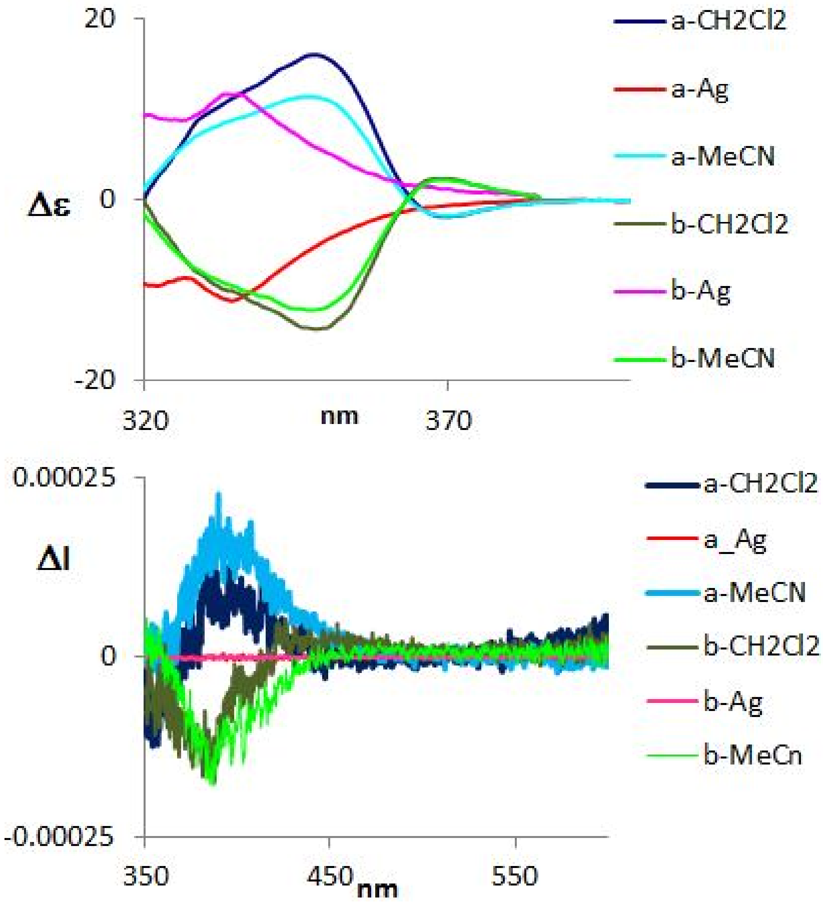

Figure S7. Fluorescence spectra of both enantiomers (**a** and **b**) of compound *p,m*-**3** in CH_2Cl_2, with addition of $AgBF_4$, and with further addition of acetonitrile. Excitation wavelength was set to 340 nm. Same experimental condition as Figure S6 (see text).

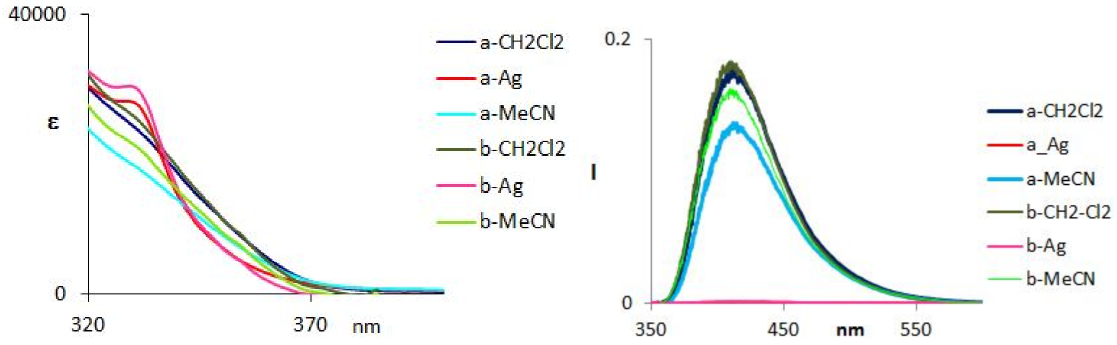

Figure S8. CPL ((a) left)and fluorescence ((b) right)spectraof compound *p,m*-**3** enantiomer, in CH_2Cl_2, with different excitation wavelengths. Gain has been adjusted in order to normalize fluorescence signal.

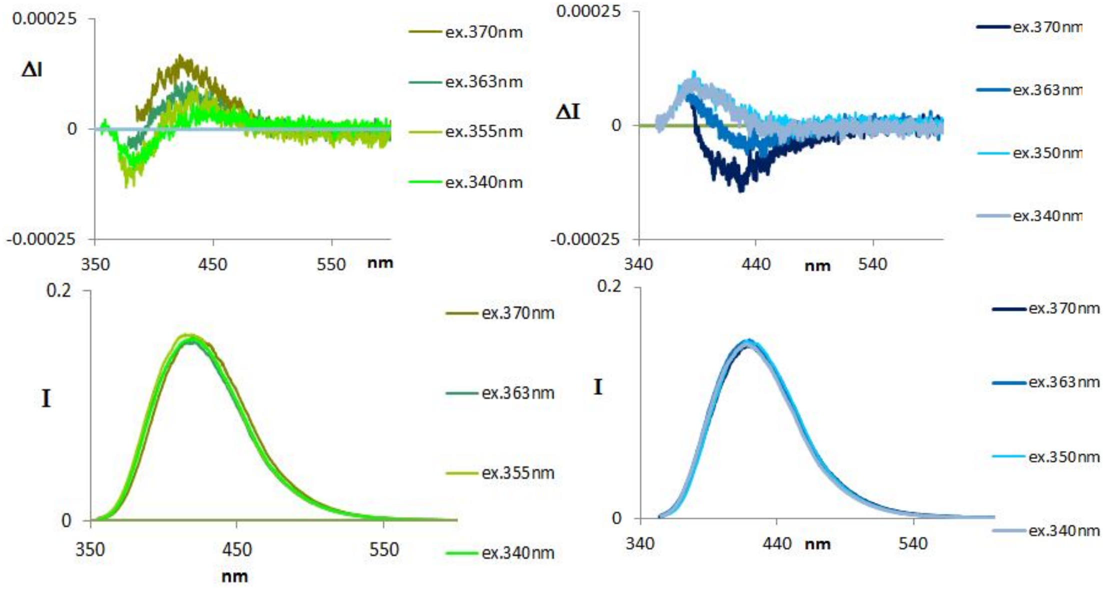

Figure S9. CD spectra ofbothdiastereoisomers **4a** and **4b** in CH_2Cl_2, measured between 250 and 400nm.

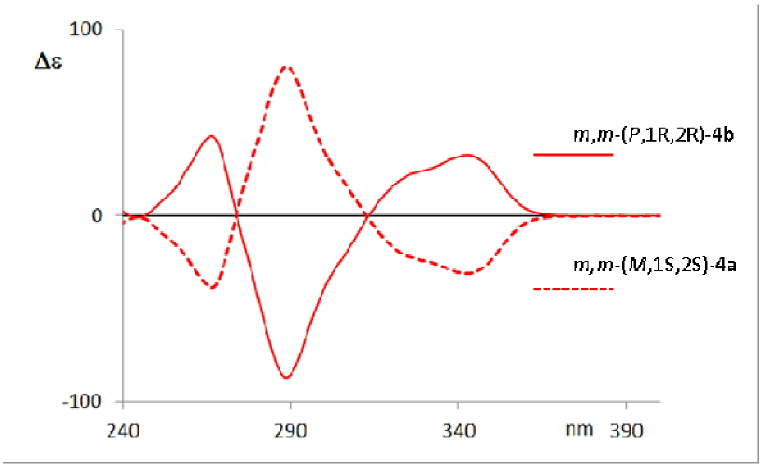

Figure S10. CPL (top) and fluorescence (bottom) spectra of diastereoisomers**4a** (1´S,4´R,M,1S,2S) and **4b** (1´S,4´R,P,1R,2R), in CH_2Cl_2 solution. Excitation wavelength was set at 335 nm.

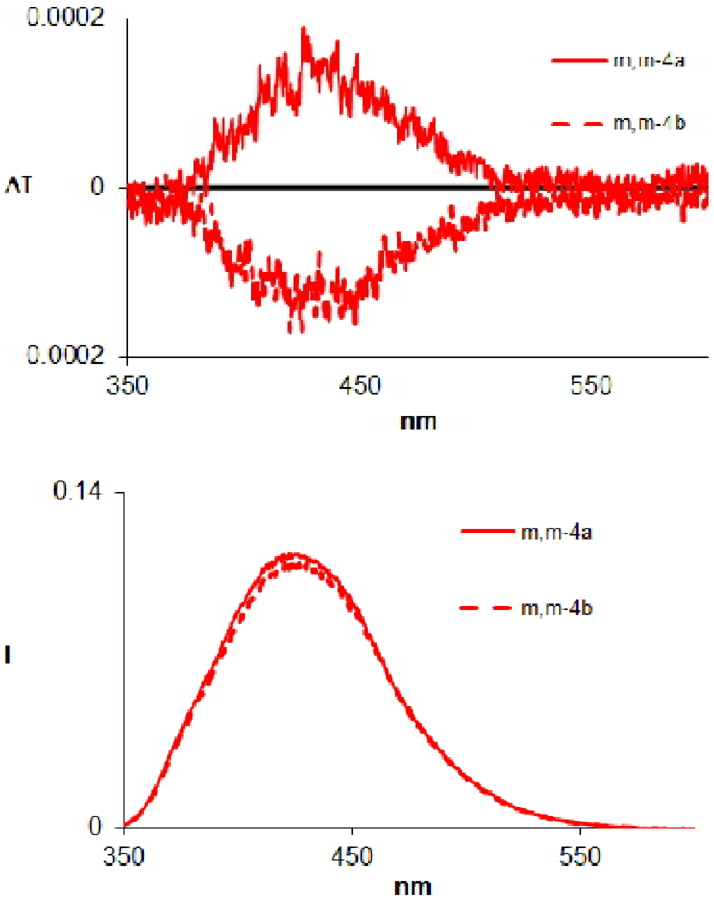

CD SPECTRA OF COMPOUNDS 1-3 IN DIFFERENT SOLVENTS

Fig. S11. CD spectra of diol (*M*, 1*R*, 2*R*)-*p*,*p*-**1** in different solvents

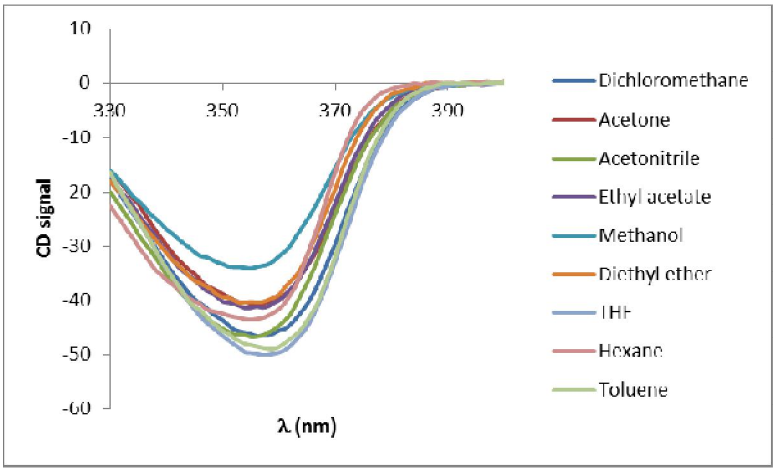

Fig. S12. CD spectra of diol (*P*, 1*R*, 2*R*)-*m*,*m*-**2** in different solvents

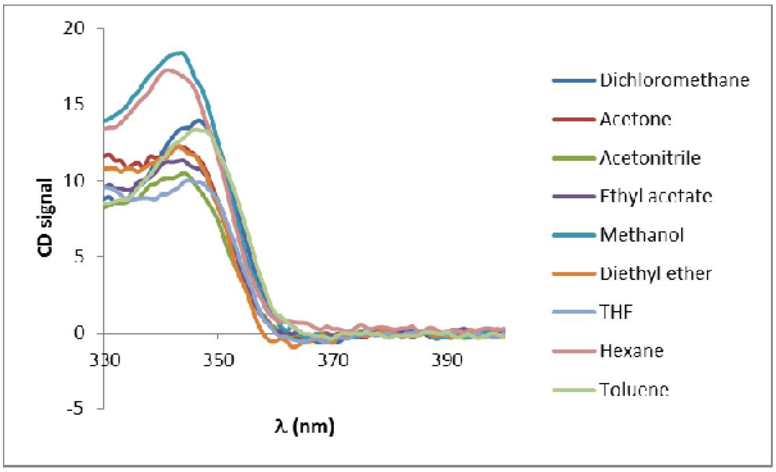

Fig. S13. CD spectra of diol *p*,*m*-**3** in different solvents

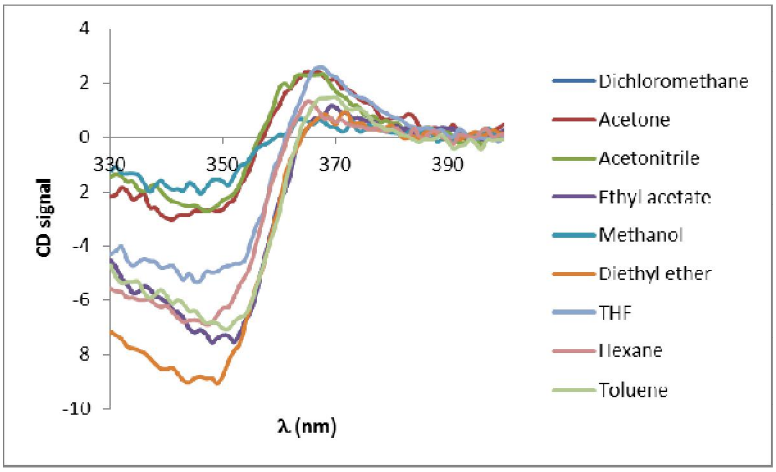

Fig. S14. CD spectra of a solution of diol (*P*, 1*S*, 2*S*)-*p*,*p*-**1** in 1,2-dichloroethane between 10 and 70ºC.

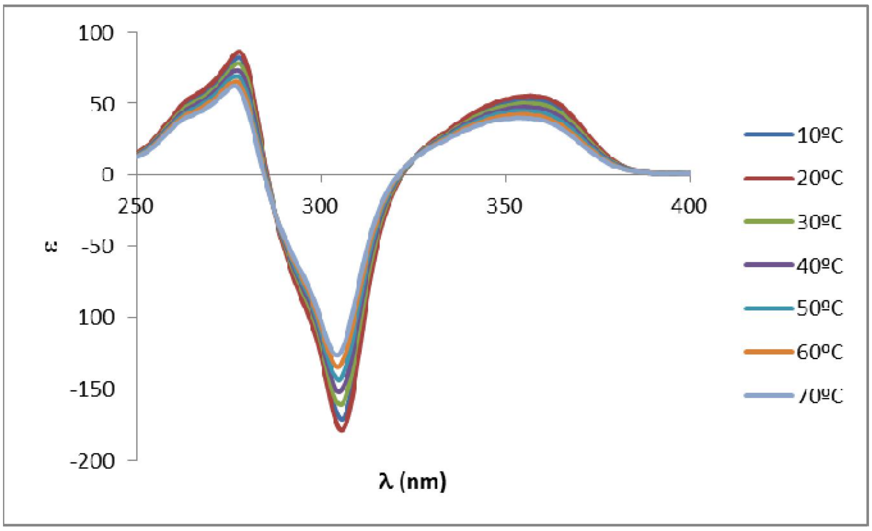

Fig. S15. CD spectra of a solution of diol (*P*, 1*R*, 2*R*)-*m*,*m*-**2**in 1,2-dichloroethane between 10 and 70ºC.

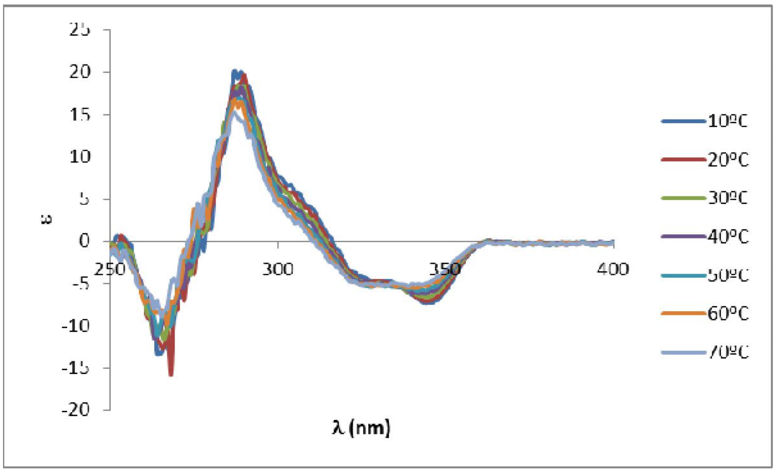

Fig. S16. CD spectra of a solution of diol *p*,*m*-**3** in 1,2-dichloroethane between 10 and 70 ºC.

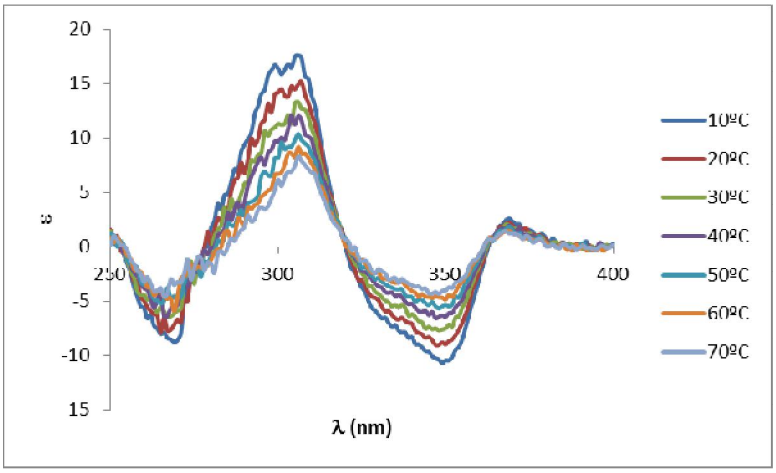

LIFETIMES, QUANTUM YIELDS, TRES DECOMPOSITION AND PHOTODEGRADATION OF COMPOUNDS 1-4

Time-resolved fluorescence decay traces were collected in single photon timing (SPT) mode on a FluoTime 200 fluorometer (PicoQuant, GmbH). The excitation source was a 375-nm pulsed diode laser (LDH-P-C-375BPicoQuant, GmbH) using a 20 MHz excitation frequency. The full width at half maximum (fwhm) of the laser pulses was around 40 ps. The fluorescence emission was collected at a 90° geometry, focused at the detector after crossing through a polarizer (set at the magic angle), 2-mm slits, and a 2-nm bandwidth monochromator. SPT was achieved by a TimeHarp200 board, set at 36 ps/channel. Fluorescence decay traces were collected for the necessary time to reach 20,000 counts at the peak channel. Time-resolved emission spectroscopy (TRES) of compounds **1**-**4** dissolved in CH_2Cl_2 was performed by collecting 55 fluorescence decay traces in the 390-500 nm emission range ($\Delta\lambda_{em}$ = 2 nm) during a fixed amount of time, to maintain the overall intensity information.

The fluorescence decay traces were fitted to a two-exponential function, by using a Levenberg-Marquard algorithm-based nonlinear least-squares error minimization deconvolution method iterative reconvolution methods (FluoFit 4.4 package, Picoquant GmbH). For each sample, the decay traces collected at different emission wavelengths were fitted globally with the decay times linked as shared parameters, whereas the pre-exponential factors were local adjustable parameters. The quality of fittings was assessed by the value of the reduced chi-squared, χ^2, parameter and random distributions of the weighted residuals and the autocorrelation functions.

For the TRES (Time Resolved Emission Spectroscopy) analysis and the estimation of the species-associated emission spectra (SAEMS), the fitting procedure described above was performed, by fitting globally the 55 decay traces. The SAEMS of each species *i* at any given emission wavelength ($SAEMS_i(\lambda_{em})$) is given by the fluorescence intensity emitted by the species *i* ($A_{i,\lambda em} \times \tau_i$), normalized by the total intensity and corrected for the different detection sensitivity using the total intensity of the steady-state spectrum ($I_{ss,\lambda em}$):

$$SAEMS_i(\lambda_{em}) = \frac{A_{i,\lambda em} \times \tau_i}{\sum_i A_{i,\lambda em} \times \tau_i} \cdot I_{ss,\lambda em} \qquad \text{(eq. S1)}$$

The approximate contribution of each species can be assessed as the area under the SAEMS. This estimation assumes equal excitation rate for all the species, as the initial amount of each form in the excited state (after the pulse excitation) is unknown. Figures S14-S18 show theSAEMS of compounds **1**-**4** dissolved in dichloromethane.

To perform photostability measurements, viscous diluted solutions (≈10^{-10}M) of the compounds **1-3** in glycerol as solvent were irradiated continuously by means of a Xe lamp to cause maximum damage and thus test their phostostability. Figure S19 shows the resulting steady-state fluorescence signal *vs* time.

For the relative determination of the fluorescence quantum yield Φ in a series of solvents, the following formula was used:[6,7]

$$\Phi_x = \Phi_r \times \frac{F_x}{F_r} \times \frac{1-10^{-A_r(\lambda_{ex})}}{1-10^{-A_x(\lambda_{ex})}} \times \frac{n_x^2}{n_r^2}$$

The subscripts *x* and *r* refer respectively to sample *x* and reference (standard) fluorophore *r* with known quantum yield Φ_r in a specific solvent; *F* stands for the *spectrally corrected*, integrated fluorescence spectra; $A(\lambda_{ex})$ denotes the absorbance at the used excitation wavelength λ_{ex}; *n* represents the refractive index of the solvent (in principle at the average emission wavelength). To minimize inner filter effects, the absorbance at the excitation wavelength λ_{ex} was kept under 0.1. The measurements were performed using 10×10 mm cuvettes. Quinine in 0.1 M H_2SO_4 was used as fluorescence quantum yield reference (Φ_r = 0.59).[8] All measurements were done on non-degassed samples at 20 °C

Fig. S14. TRES deconvolution ofdiol *p,p*-***1*** in CH_2Cl_2

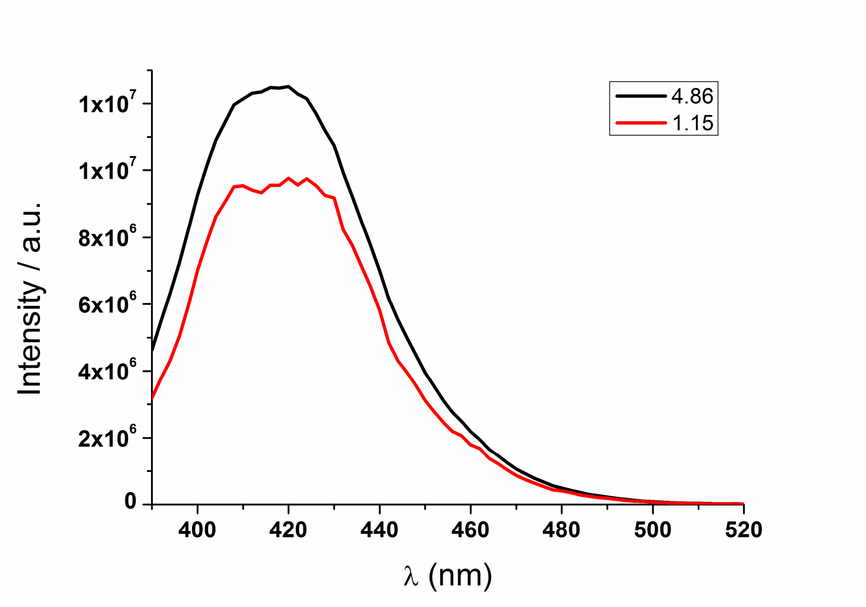

Fig. S15. TRES deconvolution of diol *m,m* **2** in CH_2Cl_2

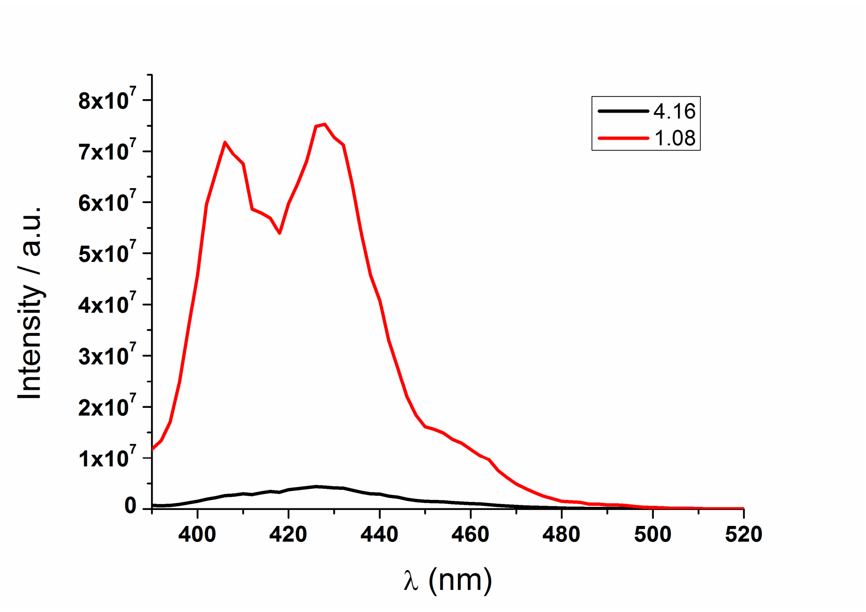

Fig. S16. TRES deconvolution of diol *m,p* **3** in CH_2Cl_2

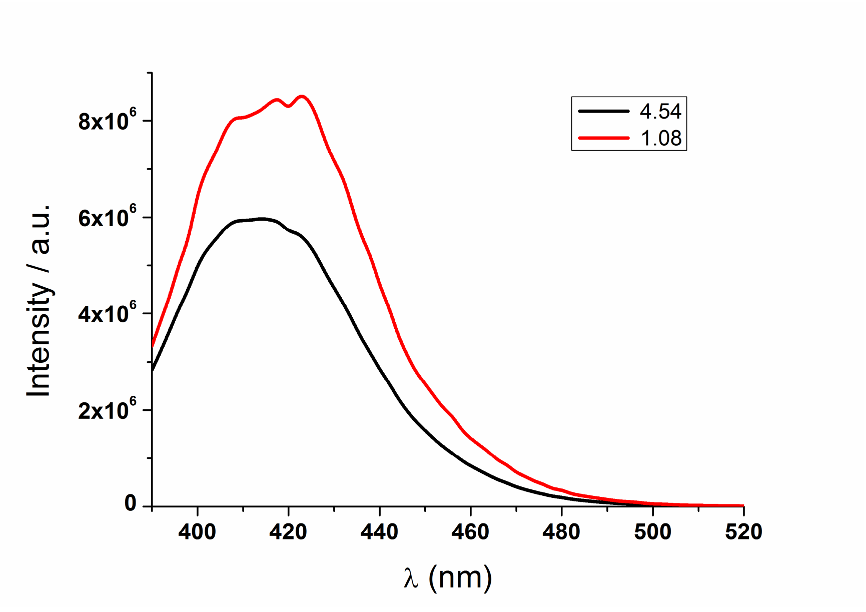

Fig. S17. TRES deconvolutionof compound**4a**in CH_2Cl_2

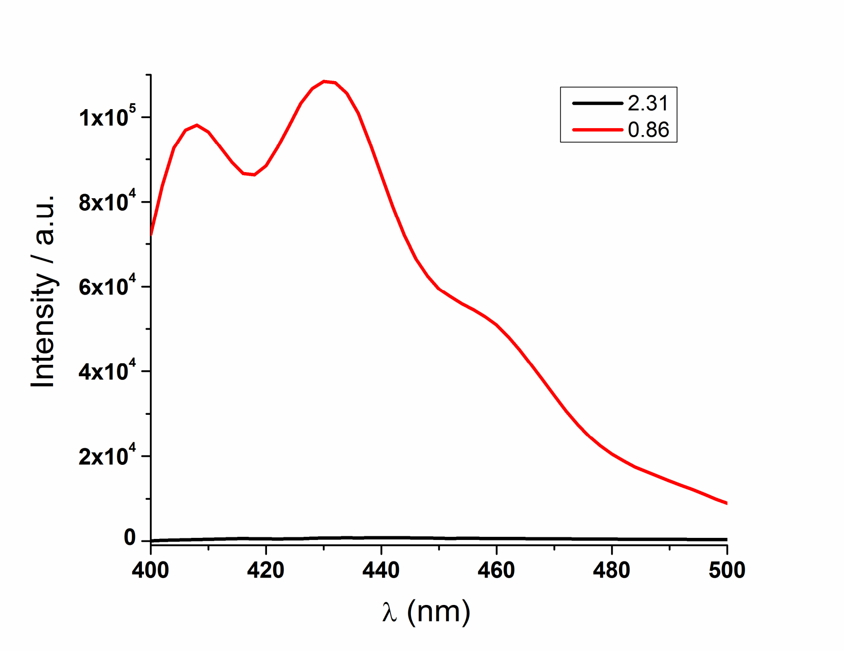

Fig. S18.TRES deconvolutionof compound**4b**in CH_2Cl_2

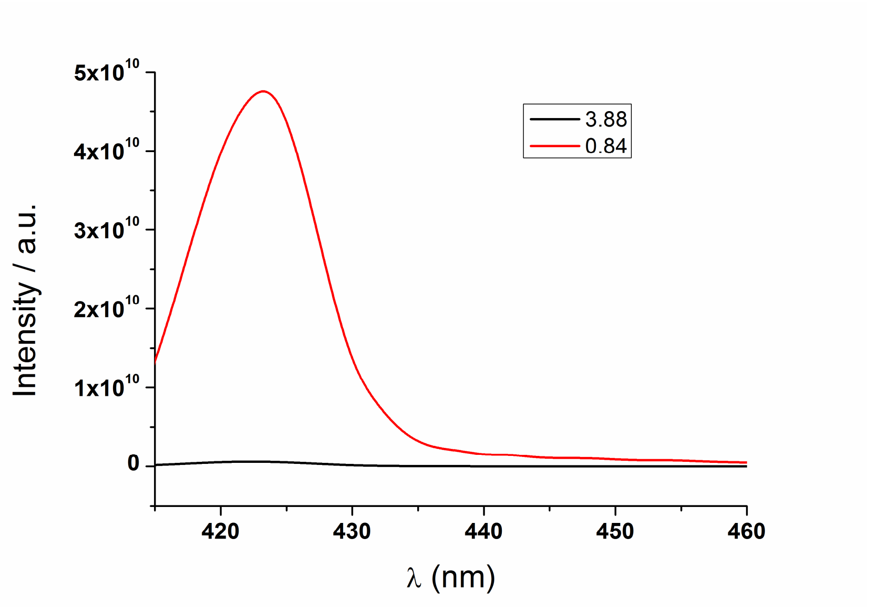

Fig. S19. Photostability of compounds **1**-**4**

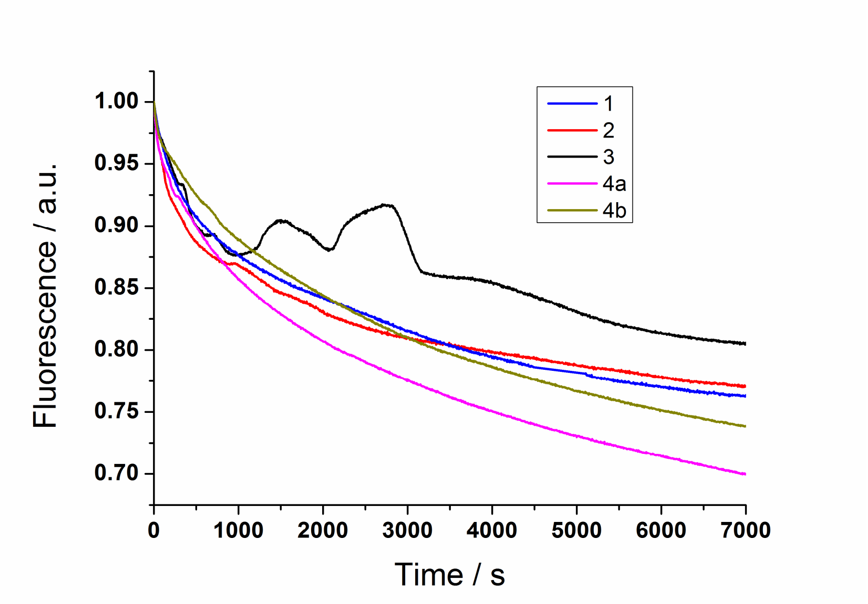

NMR TITRATIONS OF COMPOUNDS 1-4WITH Ag(I): GENERAL PROCEDURE, SPECTROSCOPIC DATAANDCOPIES OF ^{1}H-NMR AND ^{13}C-SPECTRA ofDIOL-Ag(I) COMPLEXES

*General procedure for the NMR-titration of compounds **1-4***

To perform the titrations 12mg (0.024 mmol, 1eq) of the corresponding diol**1-3**, and 24mg (0.024 mmol, 1eq) of compounds **4a-b**were dissolved in 0.5 mL of a 9:1 mixture of CD_2Cl_2:Acetone-*d*$_6$. On the other hand, a solution of 14mg (0.072 mmol, 3 eq) of $AgBF_4$ in 0.3 mL of 9:1 CD_2Cl_2: Acetone-*d*$_6$was prepared. Upon stepwise addition of 1 eq. (0.1 mL) of Ag(I) a ^{1}H-NMR spectra was performed, and changes in the aromatic signals confirmed the binding phenomena.

Spectroscopic data of diol-Ag(I) complexes

Diol *p,p*-1-Ag(I) complex:

^{1}H NMR (500 MHz, CD_2Cl_2) δ 7.92 (d, *J* = 8.7 Hz, 2H), 7.84 (d, *J* = 8.6 Hz, 2H), 7.64–7.55 (m, 4H), 7.44 (d, *J* = 8.7 Hz, 4H), 6.71 (d, *J* = 8.7 Hz, 4H), 4.44–4.39 (m, 2H), 4.37–4.32 (m, 4H); **^{13}C NMR** (125 MHz, CD_2Cl_2) δ 160.9 (C), 135.0 (CH), 134.8 (CH), 134.0 (CH), 131.1 (CH), 130.9 (CH), 125.6 (C), 123.1 (C), 115.9 (CH), 110.2 (C), 96.2 (C), 93.0 (C), 82.9 (C), 75.4 (CH), 68.0 (CH_2).

Diol*m,m*-2-Ag(I) complex:

^{1}H NMR (400 MHz, CD_2Cl_2) δ 7.93–7.83 (m, 2H), 7.83–7.77 (m, 2H), 7.60–7.52 (m, 4H), 7.09 (s, 4H), 6.84 (s, 4H), 4.33–4.13 (m, 6H);**^{13}C NMR** (100 MHz, CD_2Cl_2) δ 158.51 (C), 158.46 (C), 134.8 (CH), 134.4 (CH), 134.0 (CH), 133.7 (CH), 131.2 (CH), 131.1 (CH), 131.0 (CH), 130.8 (CH), 130.7 (CH), 130.5 (CH), 125.6 (CH), 125.5 (CH), 125.0 (C), 124.9 (C), 123.4 (C), 123.2 (C), 119.9 (C), 119.7 (C), 118.9 (CH), 118.7 (CH), 117.8 (CH), 117.6 (CH), 95.2 (C), 95.1 (C), 92.8 (C), 92.6 (C), 84.8 (C), 84.6 (C), 75.4 (CH), 70.9 (CH), 68.7 (CH_2), 67.8 (CH_2).

Diol *p,m*-3-Ag(I) complex:

^{1}H NMR (500 MHz, CD_2Cl_2) δ7.92 (d, *J* = 7.5 Hz, 2H), 7.83 (t, *J* = 8.1 Hz, 2H), 7.65–7.53 (m, 4H), 7.40–7.35 (m, 3H), 7.26 (d, *J* = 7.6 Hz, 1H), 7.01 (d, *J* = 6.6 Hz, 1H), 6.73 (d, *J* = 8.4 Hz, 2H), 6.45 (s, 1H), 4.53–4.45 (m, 2H), 4.33 (d, *J* = 10.0 Hz, 1H), 4.24–4.18 (m, 2H), 4.12 (s, 1H), 2.5 (bs, 2H);**^{13}C NMR** (125 MHz, CD_2Cl_2) δ159.9 (C), 158.0 (C), 134.8 (CH), 134.5 (CH), 133.6 (CH), 133.4 (CH), 133.3 (CH), 130.63 (CH), 130.60 (CH), 130.4 (CH), 130.0 (CH), 125.4 (CH), 124.8 (C), 124.7 (C), 122.8 (C), 122.5 (C), 119.5 (CH), 118.5 (C), 115.3 (CH), 114.2 (CH), 108.4 (C), 95.0 (C), 92.9 (C), 92.4 (C), 81.7 (C), 72.3 (CH), 72.1 (CH), 67.6 (CH_2), 67.0 (CH_2).

Compound 4a-Ag(I) complex

^{1}H NMR (500 MHz, CD_2Cl_2) δ7.83 (d, *J* = 7.1 Hz, 2H), 7.74 (d, *J* = 7.1 Hz, 2H), 7.58–7.49 (m, 4H), 7.17–7.09 (m, 4H), 6.89 (d, *J* = 7.4 Hz, 2H), 6.84 (s, 2H), 5.66 (br s, 2H), 4.40–4.31 (m, 4H), 2.47–2.42 (m, 2H), 2.02–1.93 (m, 4H), 1.68–1.61 (m, 2H), 1.10 (s, 6H), 1.07 (s, 6H), 0.94 (s, 6H);^{13}C NMR (125 MHz, CD_2Cl_2)δ178.4 (C), 167.1 (C), 158.1 (C), 134.1 (CH), 133. (CH), 130.7 (CH), 130.5 (CH), 130.5 (CH), 126.2 (CH), 125.1 (C), 123.9 (C), 121.0 (C), 118.9 (CH), 117.0 (CH), 94.7 (C), 92.6 (C), 91.5 (C), 85.6 (C), 72.1 (CH), 66.3 (CH_2), 55.4 (C), 55.0 (C), 40.3 (CH_2), 31.4 (CH_2), 29.5 (C), 17.2 (CH_3), 17.1 (CH_3), 9.9 (CH_3).

Compound 4b-Ag(I) complex

^{1}H NMR (500 MHz, CD_2Cl_2) δ 7.86 (d, *J* = 6.8 Hz, 2H), 7.77 (d, *J* = 8.0 Hz, 2H), 7.60–7.53 (m, 4H), 7.20–7.11 (m, 4H), 6.91 (d, *J* = 7.0 Hz, 2H), 6.86 (s, 2H), 5.69 (s, 2H), 4.44–4.35 (m, 4H), 2.54–2.46 (m, 2H), 2.02–1.94 (m, 4H), 1.71–1.63 (m, 2H), 1.08 (s, 6H), 1.07 (s, 6H), 0.97 (s, 6H);**^{13}C NMR** (125 MHz, CD_2Cl_2)δ 178.1 (C), 166.7 (C), 157.5 (C), 133.4 (CH), 133.1 (CH), 130.1 (CH), 129.9 (CH), 129.9 (CH), 125.5 (CH), 124.4 (C), 123.3 (C), 120.3 (C), 118.2 (CH), 116.2 (CH), 94.2 (C), 91.9 (C), 90.9 (C), 85.1 (C), 71.3 (CH), 65.5(CH_2), 54.8 (C), 54.5 (C), 39.6(CH_2), 30.8 (CH_2), 16.5 (CH_3), 16.4(CH_3), 9.30(CH_3).

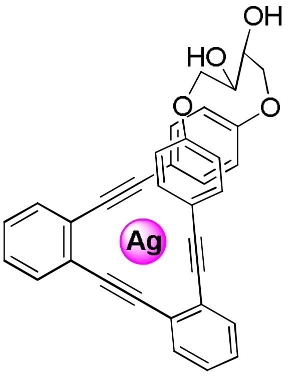
OH
HO
O
O
Ag

***p*,*p*-1-Ag**

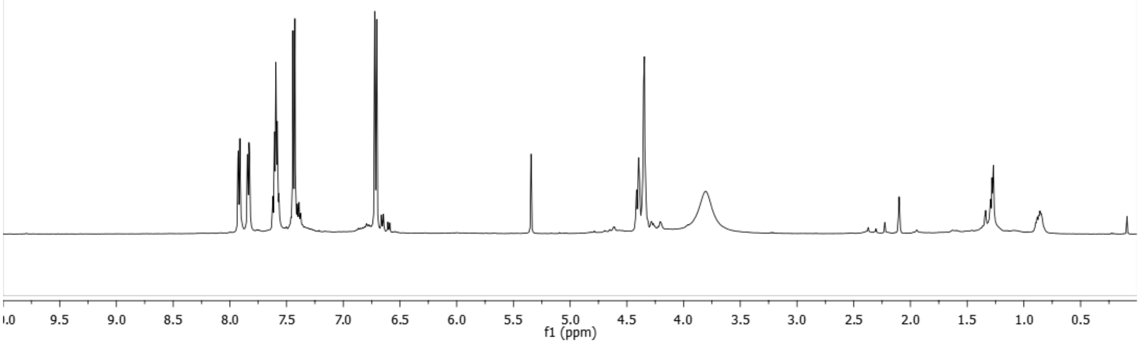
9.5 9.0 8.5 8.0 7.5 7.0 6.5 6.0 5.5 5.0 4.5 4.0 3.5 3.0 2.5 2.0 1.5 1.0 0.5
f1 (ppm)

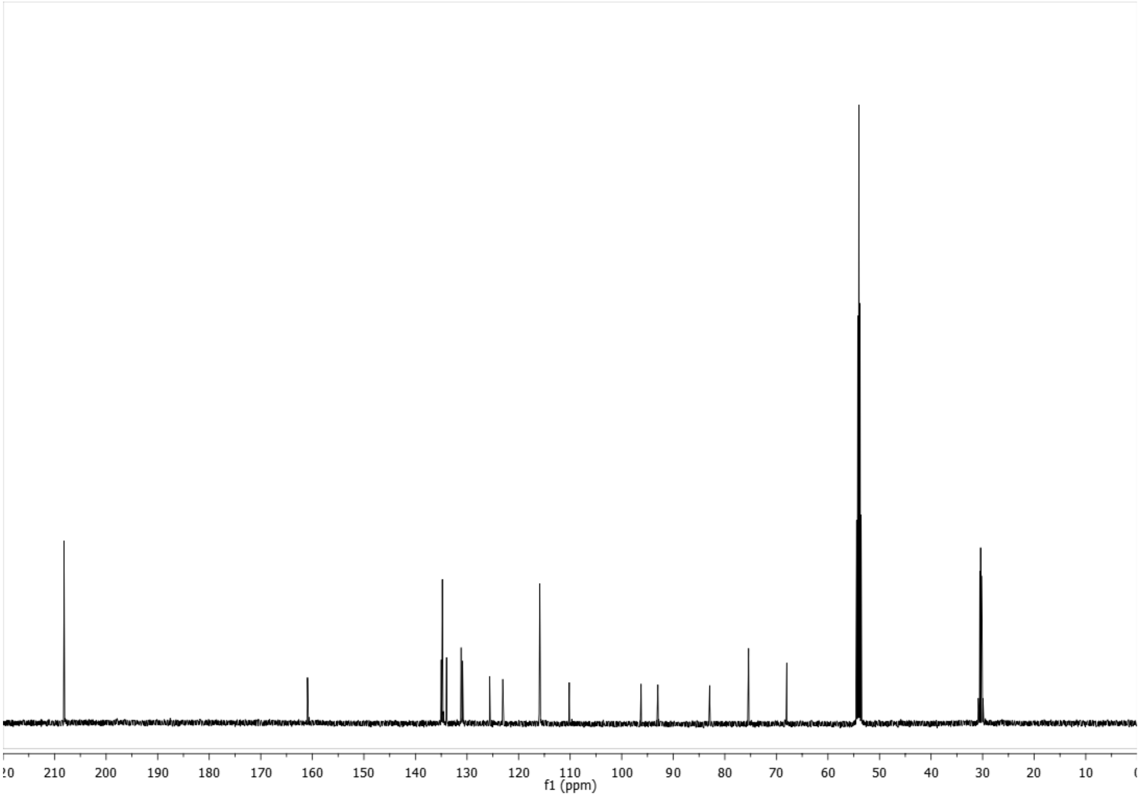
210 200 190 180 170 160 150 140 130 120 110 100 90 80 70 60 50 40 30 20 10
f1 (ppm)

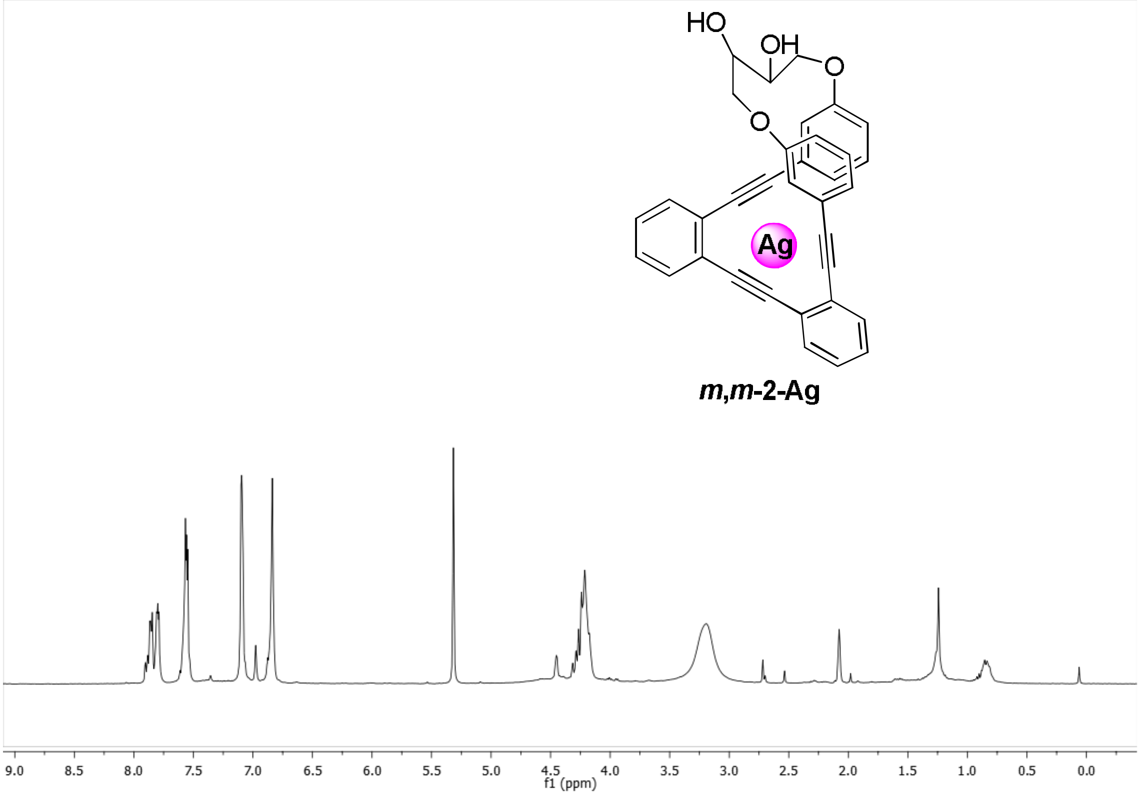
HO
OH
O
O
Ag
m,m-2-Ag
9.0
8.5
8.0
7.5
7.0
6.5
6.0
5.5
5.0
4.5
4.0
3.5
3.0
2.5
2.0
1.5
1.0
0.5
0.0
f1 (ppm)

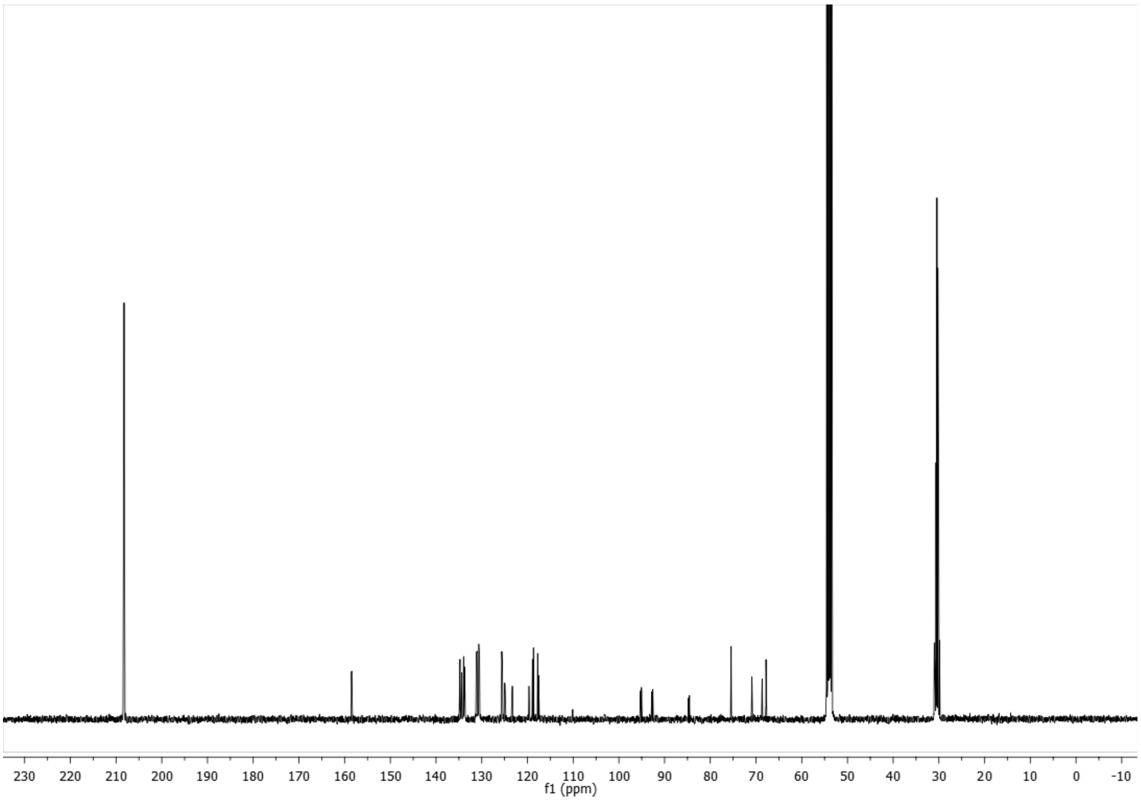
230
220
210
200
190
180
170
160
150
140
130
120
110
100
90
80
70
60
50
40
30
20
10
0
-10
f1 (ppm)

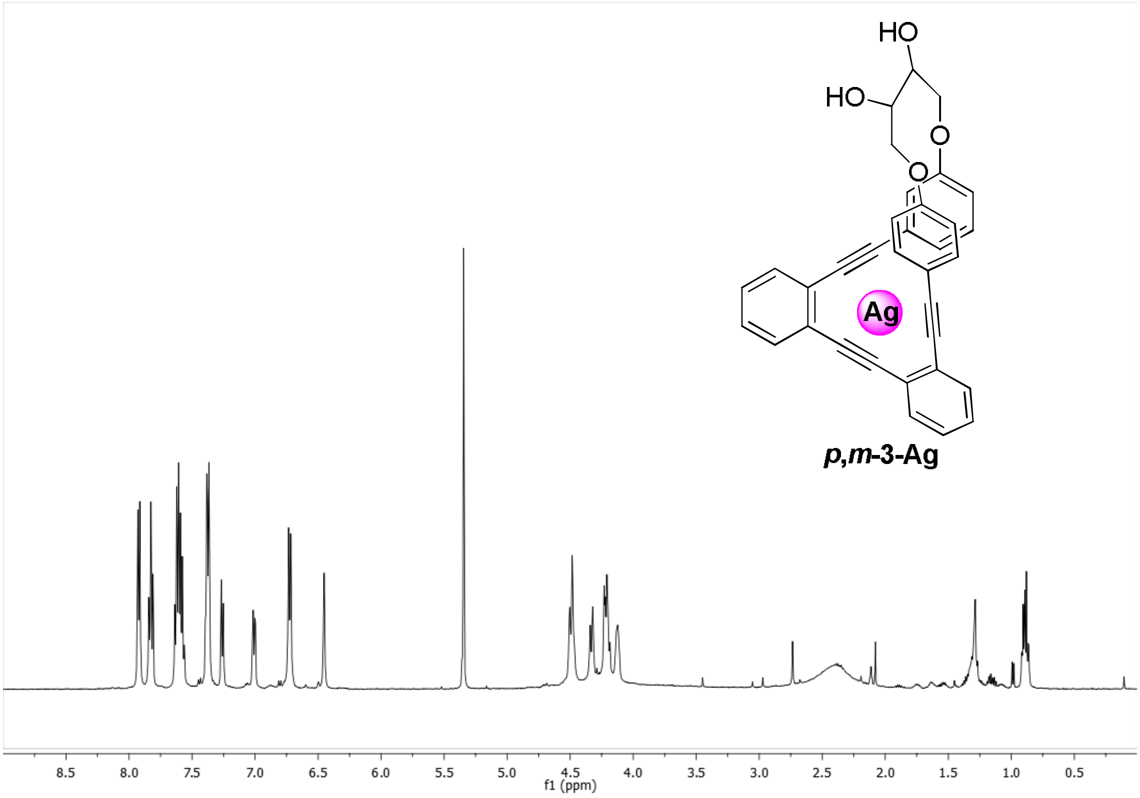
HO
HO
O
O
Ag
p,m-3-Ag
8.5
8.0
7.5
7.0
6.5
6.0
5.5
5.0
4.5
4.0
3.5
3.0
2.5
2.0
1.5
1.0
0.5
f1 (ppm)

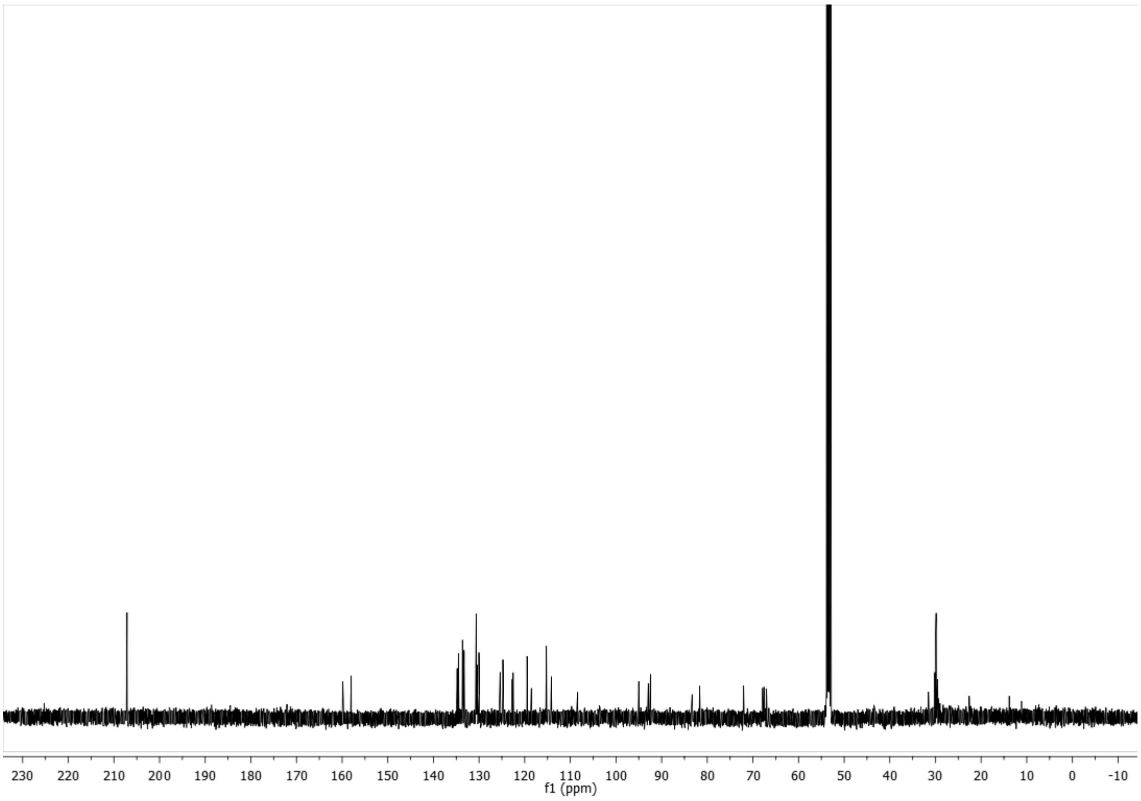
230
220
210
200
190
180
170
160
150
140
130
120
110
100
90
80
70
60
50
40
30
20
10
0
-10
f1 (ppm)

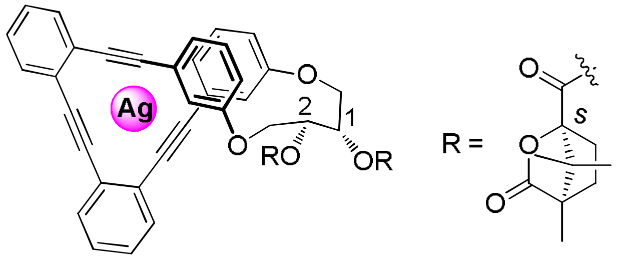

m,m-(*1´S,4´R,M,1S,2S*)-**4a_Ag**

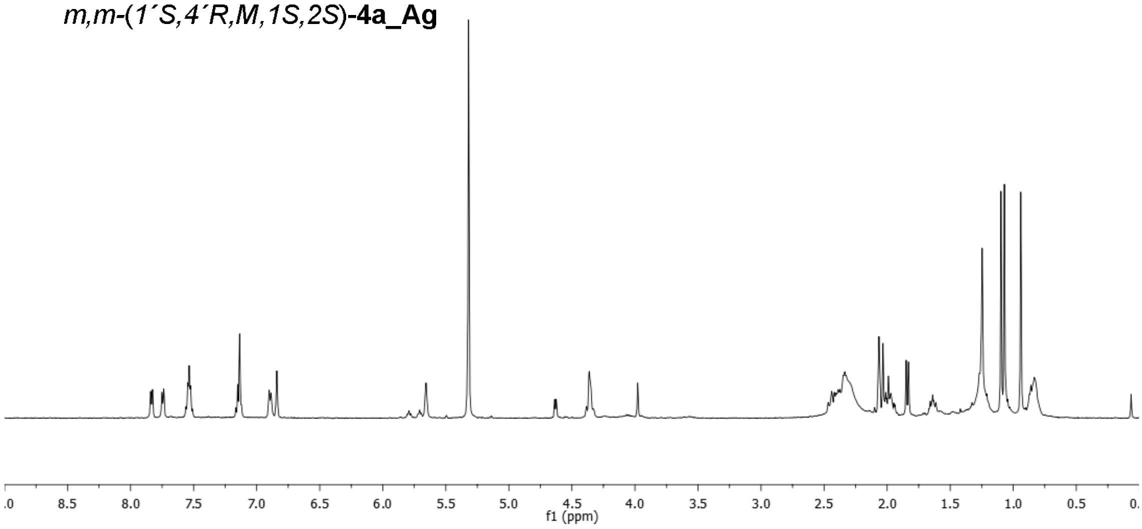

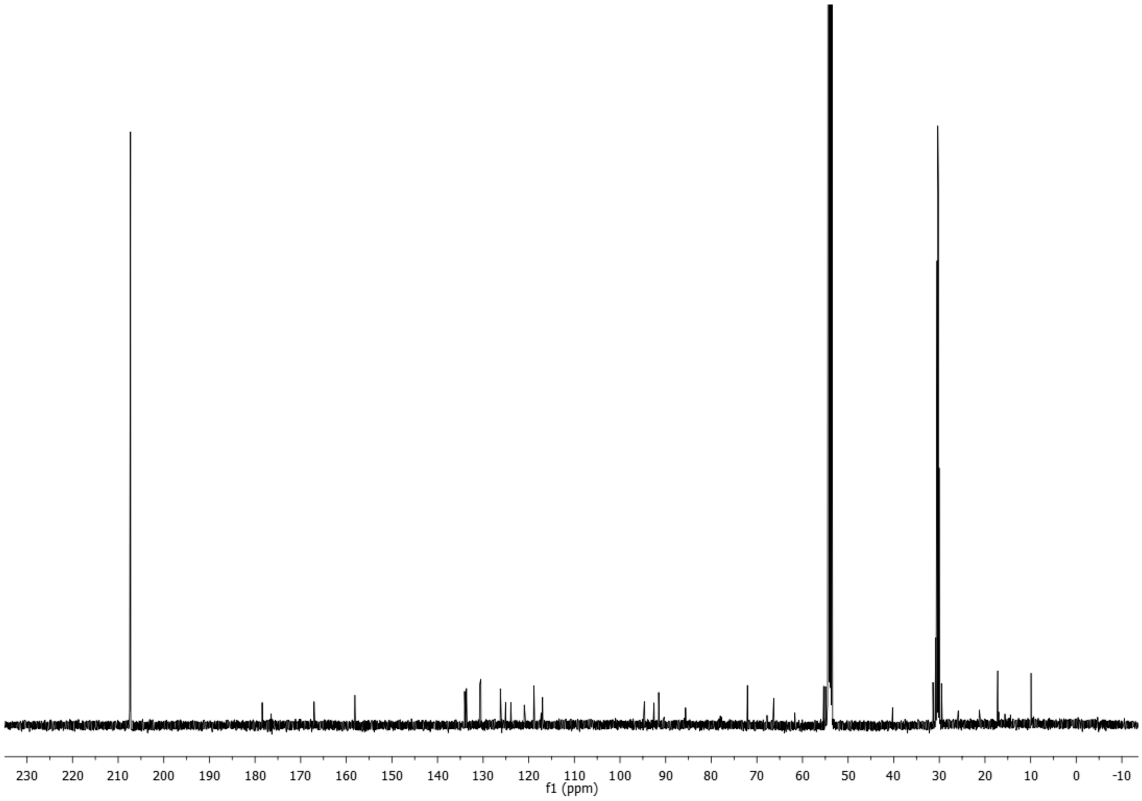

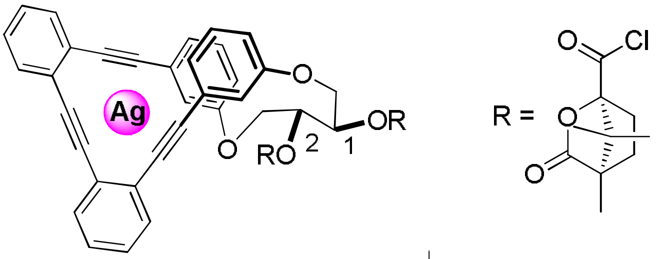

m,m-(*1´S,4´R,P,1R,2R*)-**4b_Ag**

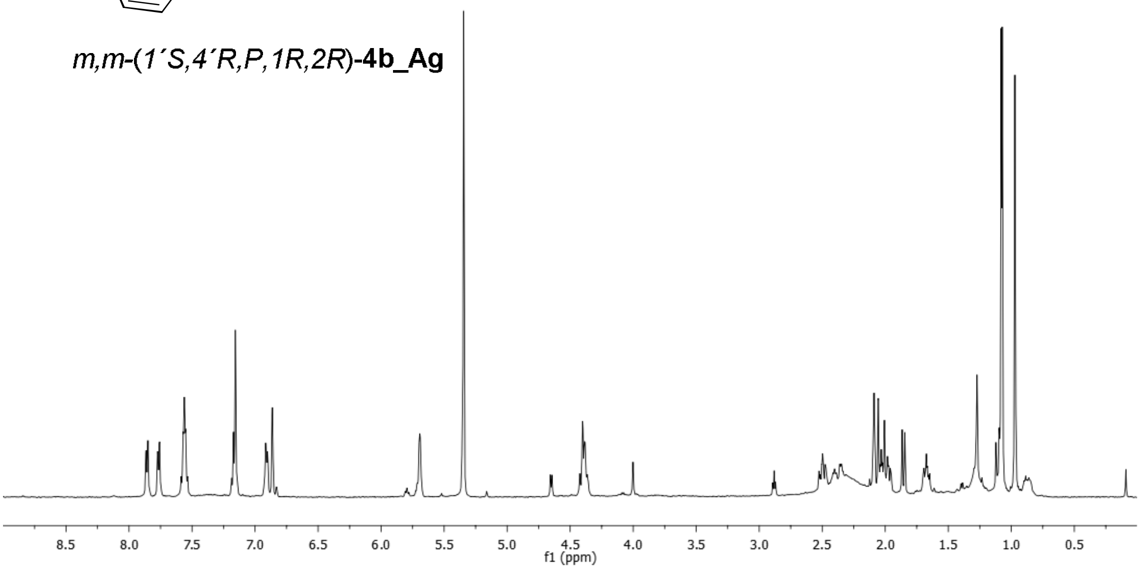

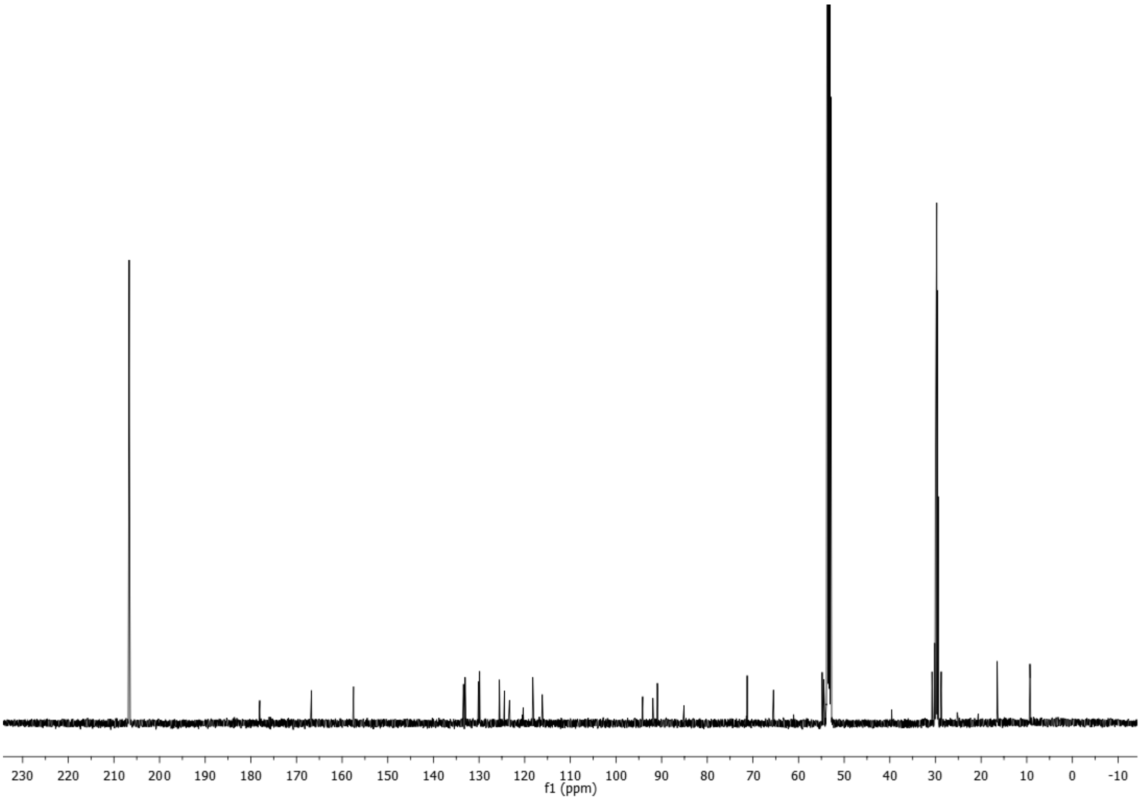

CD TITRATIONS OF COMPOUNDS 1-4 WITHAg(I)

Experimental

CD titrations were performed in a JASCO J-15 Spectropolarimeter with a 1.0 cm path-length quartz cell. Temperature was set at 10ºC using a Peltier JASCO PTC-348 WI to avoid evaporation of the solvent.

General procedure for the CD-titration of compounds **1**-**4**

Titrations of compounds **1-4** were carried out by addition of progressive quantities of a 2.5x10^{-4} M solution of $AgBF_4$ salt, which was commercially available, to a 2.5x10^{-5} M solution of the corresponding diol **1-3** or camphanoyl derivative **4a-4b**. The samples were prepared by solving 1.1 mg of compounds **1-4** in 4.7mL for **1-3** and 2.6 mL for **4a-4b** of a 95:5 mixture of CH_2Cl_2:acetone. To make the fitting of the kinetic constant easier, concentration of ligands **1-4** was kept constant during the titration. To ensure this, 2.5x10^{-5}M solution of compounds **1-4** was used as solvent to prepare the $AgBF_4$ solution. The fitting was carried out with DynaFit program (v. 4.06019), which has been previously used to study guest-host complexation equilibria.[9]

CD spectra and Dynafit results of the titrations of compounds **1**-**4**

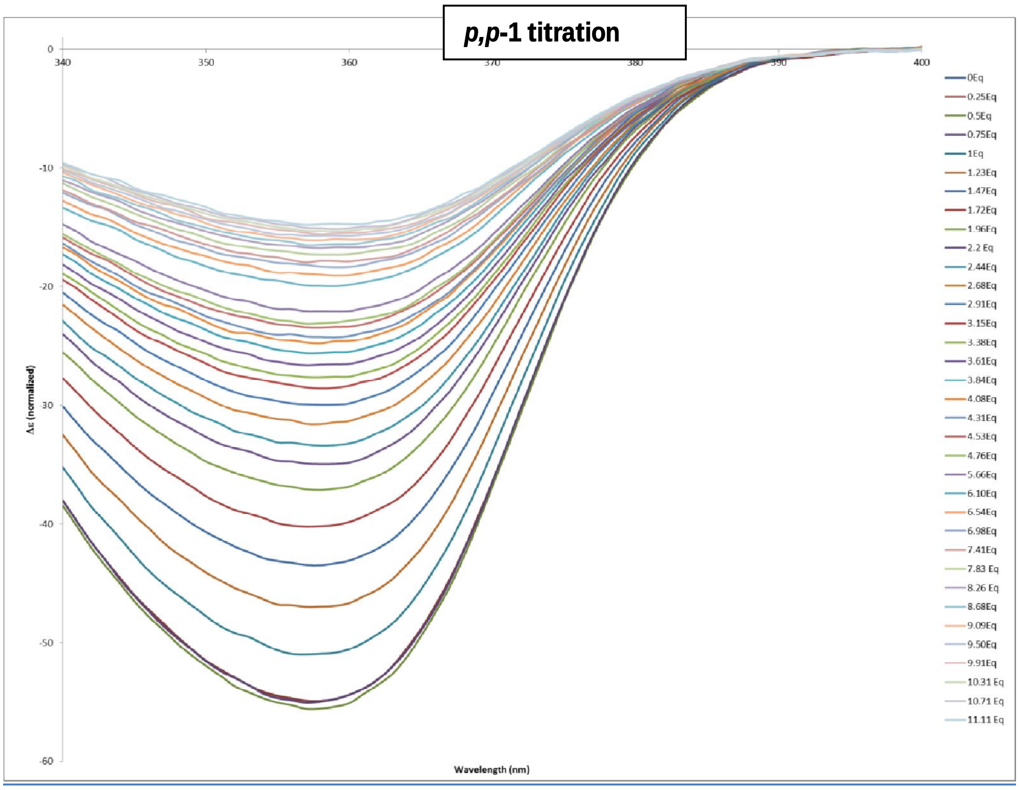

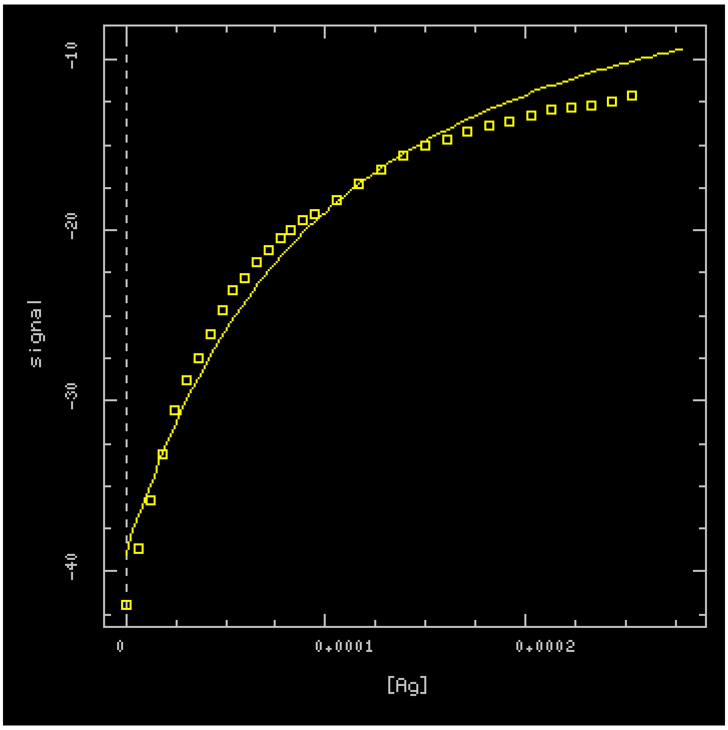

OptimizedParameters

No.	Par# Set	Initial	Final	Std. Error	CV (%)	Low	Low P (%)	High	High P (%)
**#1**	K	20000	**12211.3**	635.516	5.20				
**#2**	r(L)	-1.8e+006	**-1.56e+006**	27746.2	1.78	-1.6e+006	95	-1.5e+006	95

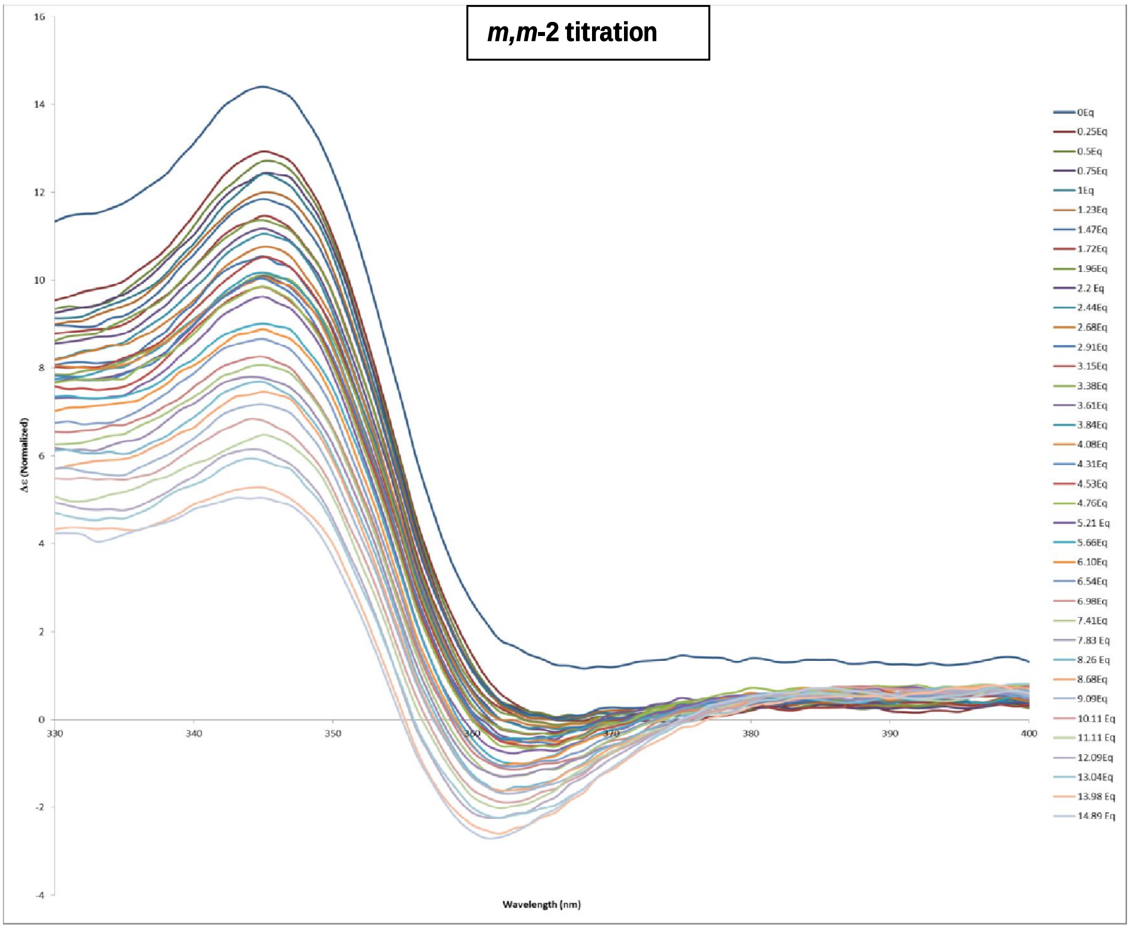

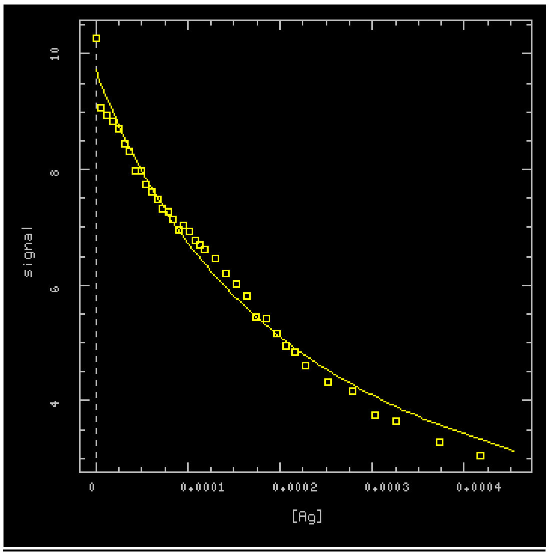

Optimized Parameters

No.	Par#Set	Initial	Final	Std. Error	CV (%)	Low	Low P (%)	High	High P (%)
**#1**	K	20000	**4805.17**	160.957	3.35				
**#2**	r(L)	400000	**388846**	3613.53	0.93	381630	95	396182	95

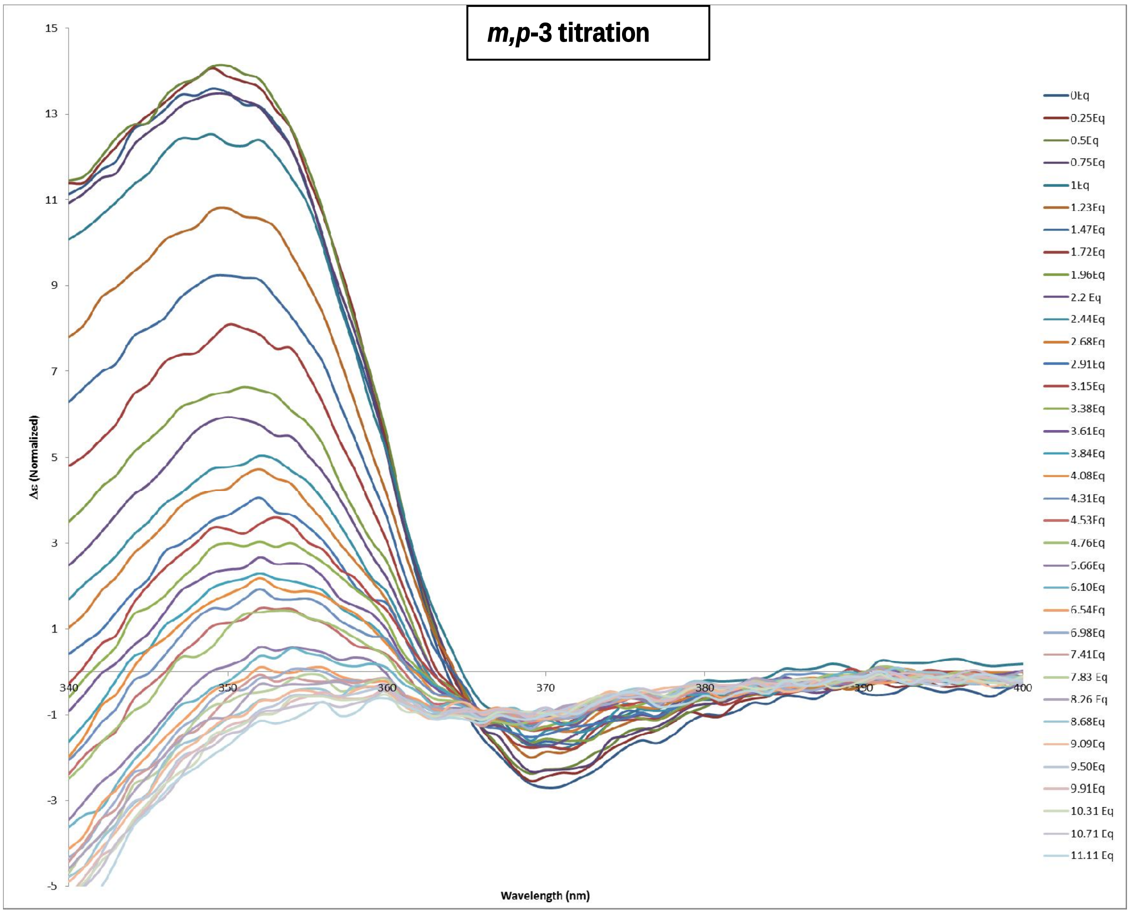

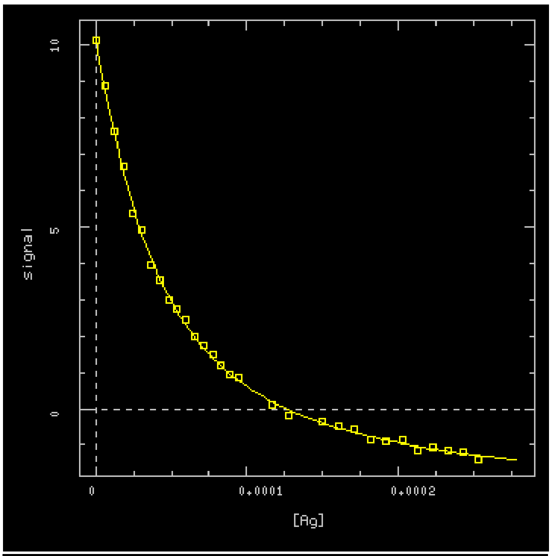

OptimizedParameters

No.	Par#Set	Initial	Final	Std. Error	CV (%)	Low	Low P (%)	High	High P (%)
#1	K	20000	**35925.9**	1064.2	2.96				
#2	r(L)	445200	**408041**	2921.02	0.72	402047	95	414034	95
#3	r(LAg)	-56000	**-107024**	2801.09	2.62	-112878	95	-101342	95

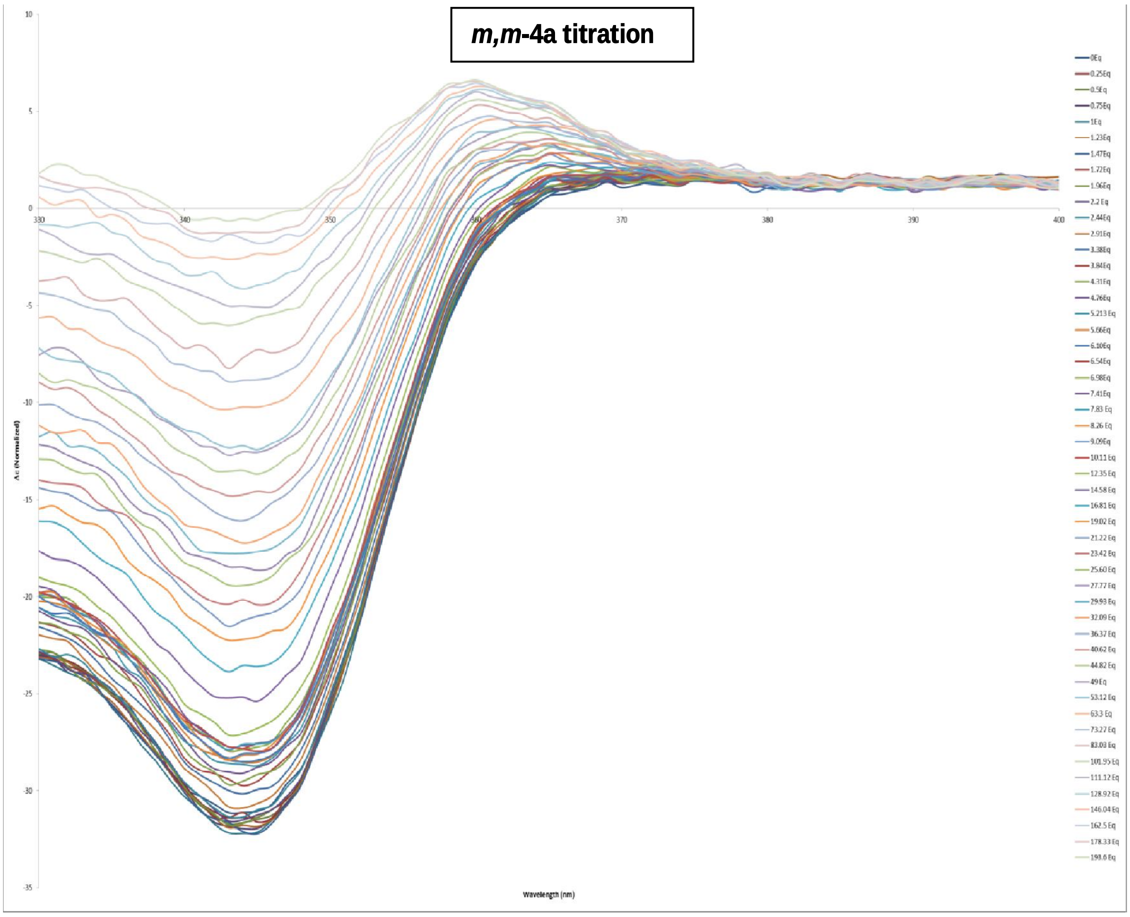

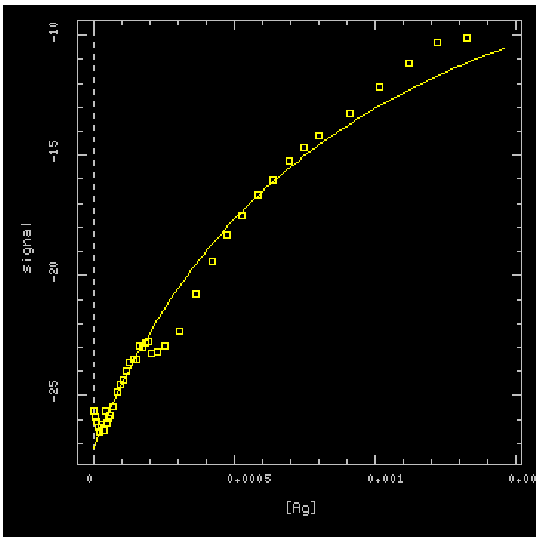

Optimized Parameters

No.	Par# Set	Initial	Final	Std. Error	CV (%)	Low	Low P (%)	High	High P (%)
**# 1**	K	400	**1098.99**	41.8904	3.81	1018.75	95	1183.72	95
**# 2**	r(L)	-1.06e+006	**-1.09e+006**	8229.9	0.76	-1.10525e+006	95	-1.07e+006	95

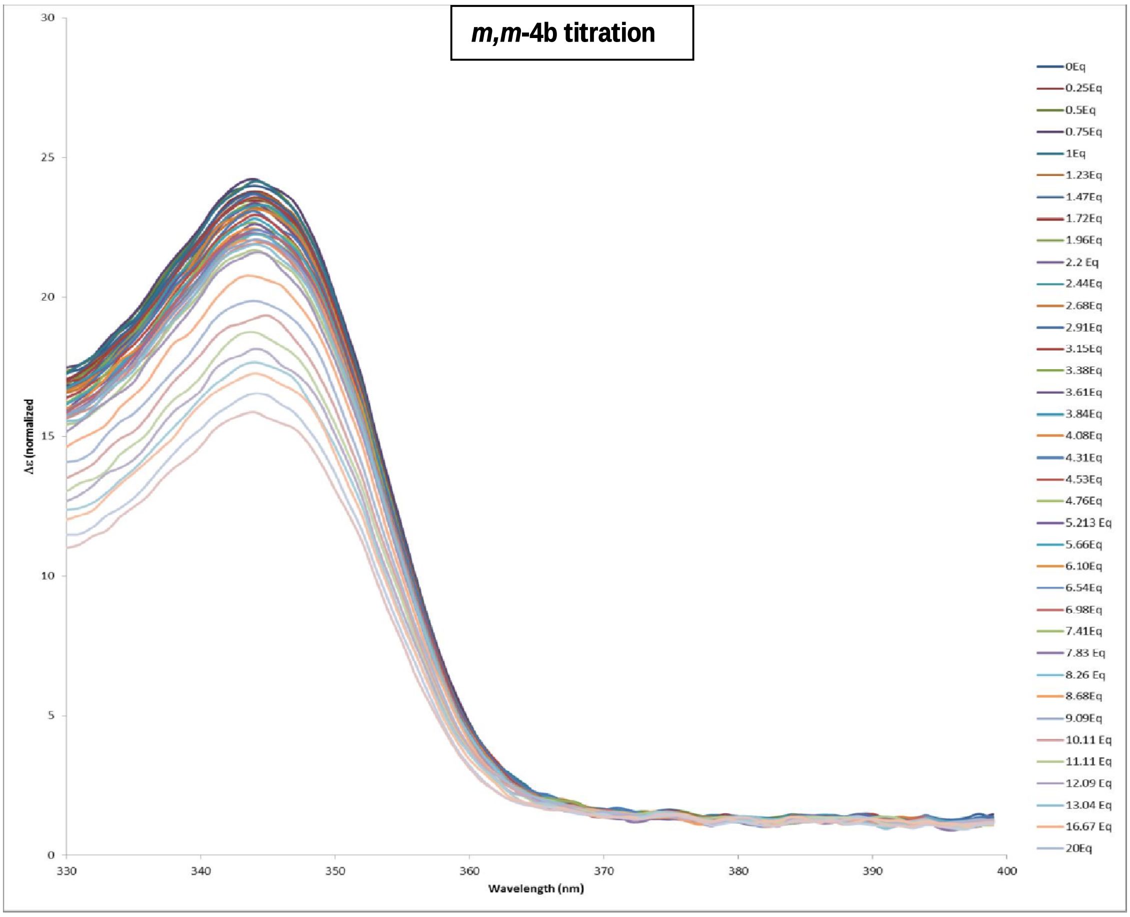

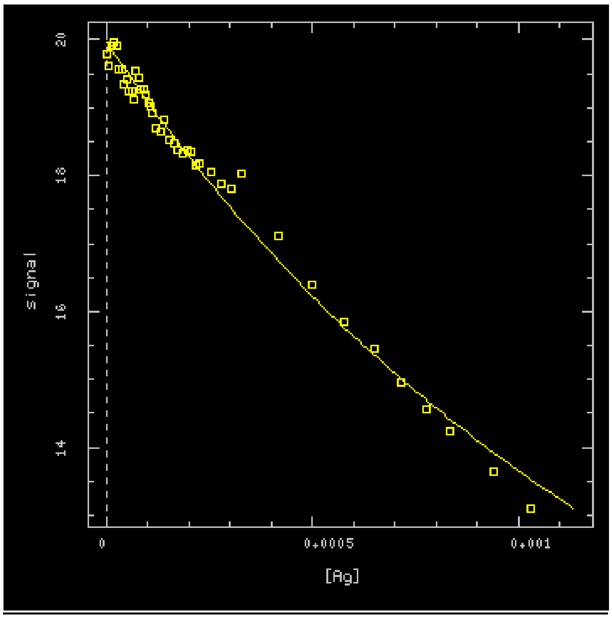

Optimized Parameters

No.	Par#Set	Initial	Final	Std. Error	CV (%)	Low	Low (%) P	High	High (%) P
**#1**	K	400	**465.577**	10.5973	2.28	444.548	95	486.964	95
**#2**	r(L)	960000	**798499**	1924.88	0.24	794636	95	802384	95

THEORETICAL CALCULATIONS

Molecular mechanics (MM) conformational search have been performed for molecules **1**, **2** and **3**. Conformers within 5 kcal/mol were all optimized at B3LYP/6-31g* level.

Populated conformers have been optimized also within the framework of polarizable continuum model approximation (PCM), checking that all structures correspond to minima and evaluating the Gibbs free energy.

From all structures thus obtained, a new optimization has been performed in presence of Ag(I).

The time-dependent DFT (TD-DFT) method has been used to calculate Absorption and CD spectra at the same level of approximation, iefpcm B3LYP/6-31G*.

All calculations have been performed with Gaussian09 package.[10]

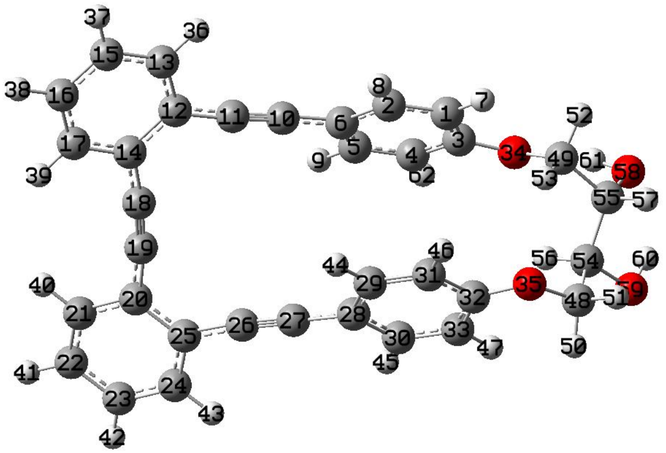

Conformational study of compound *p,p*-1 (1S,2S)

From all geometries found by Molecular Mechanics, structures within 5 Kcal/mol have been optimized at b3lyp/6-31g* level in vacuo

	Kcal/mol	pop (E)	4,3,34,49	3,34,49,55	34,49,55,54	48,54,55,49	35,48,54,55	32,35,48,54	31,32,35,48	54,55,58,61	55,54,59,60	
**1**	**0.00**	***84.7%***	**-155**	**154**	**-64**	**-70**	**70**	**75**	**-175**	**80**	**-37**	**P**
**24**	**1.54**	***6.3%***	**-161**	**69**	**61**	**-167**	**53**	**70**	**-163**	**-92**	**40**	**P**
**22**	**1.90**	***3.4%***	**156**	**-90**	**66**	**-170**	**-176**	**-75**	**-71**	**41**	**160**	**M**
**16**	**1.91**	***3.4%***	**-164**	**70**	**55**	**-166**	**60**	**69**	**-161**	**46**	**-154**	**P**
**11**	**2.64**	***1.0%***	**-160**	**61**	**43**	**-169**	**170**	**-75**	**-15**	**44**	**174**	**P**
**9**	**3.05**	***0.5%***	**-10**	**-163**	**87**	**-59**	**85**	**-165**	**-9**	**-161**	**-47**	**M**
26	3.56	*0.2%*	-15	-121	97	-60	-65	162	-164	-42	80	M
27	3.74	*0.2%*	-164	160	-52	-71	65	80	-180	-42	-150	P
3	3.99	*0.1%*	-57	-89	-179	-65	-51	131	-156	177	-41	P
13	4.18	*0.1%*	-3	-119	71	-80	-177	-86	-61	-38	169	P
4	4.36	*0.1%*	-71	-67	177	-68	-65	127	-145	-39	79	P
17	4.37	*0.1%*	-151	141	-57	-61	175	-87	-68	79	-42	P
21	4.45	*0.0%*	-74	-86	180	-65	-11	-76	5	176	-41	M
5	5.22	*0.0%*	-156	143	-42	-46	-50	138	-152	-45	73	M
8	5.58	*0.0%*	-47	-138	165	-84	67	-129	-21	-172	-32	M
7	5.59	*0.0%*	-44	-140	169	-87	59	-106	-8	-176	-31	M
29	6.31	*0.0%*	-157	156	-51	-77	61	84	180	-69	-75	P
28	6.95	*0.0%*	-156	141	-45	-46	-43	143	-157	-162	-50	M

Six conformers with not negligible populations have been optimized considering implicit solvent (iefpcm).

	Kcal	pop(E)	Kcal	pop(G)	4,3,34,49	3,34,49,55	34,49,55,54	48,54,55,49	35,48,54,55	32,35,48,54	31,32,35,48	54,55,58,61	55,54,59,60	
1	0.00	*55.4%*	0.00	*34.0%*	-158	155	-63	-71	69	76	-176	78	-37	**P**
24	0.47	*24.8%*	0.34	*19.1%*	-164	71	58	-166	56	71	-165	-94	40	**P**
16	0.74	*15.9%*	0.16	*26.0%*	-164	70	56	-167	59	70	-164	45	-162	**P**
9	2.09	*1.6%*	1.07	*5.5%*	-8	-164	86	-59	86	-165	-8	-158	-45	**M**
22	2.15	*1.5%*	0.51	*14.3%*	158	-90	66	-170	-179	-73	-62	41	162	**M**
11	2.52	*0.8%*	2.03	*1.1%*	-160	62	43	-170	168	-74	-17	44	174	**P**

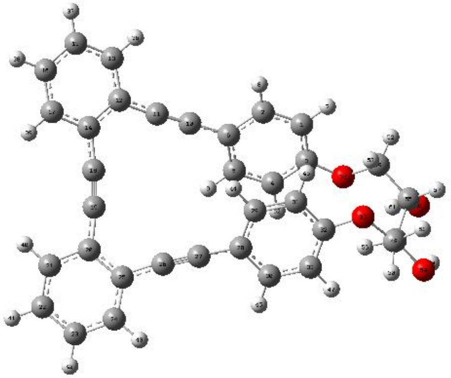

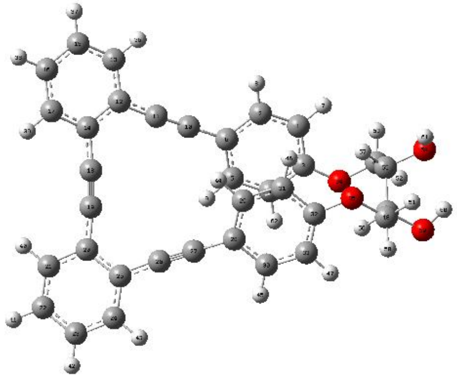

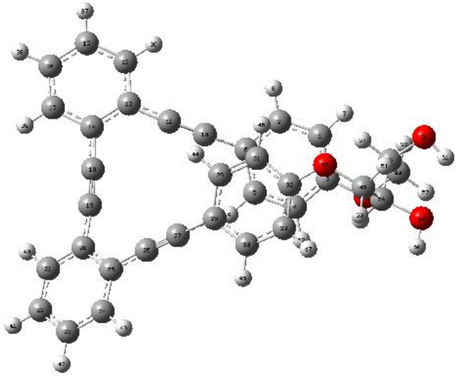

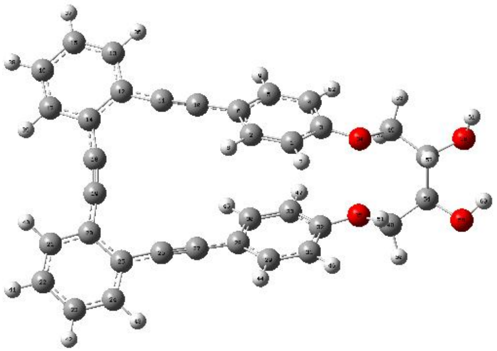

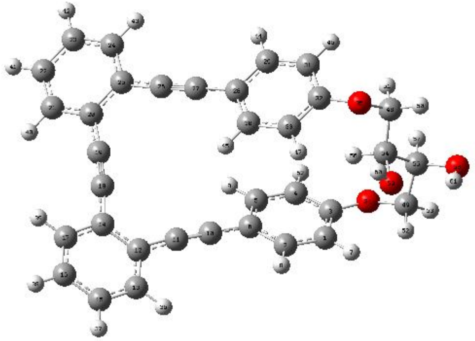

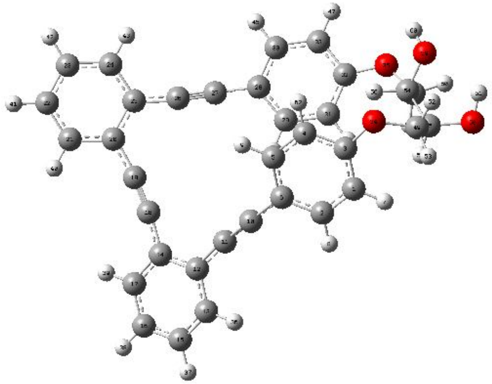

The structures thus obtained have been optimized with an Ag atom (iefpcm, b3lyp/6-31g*)

	Kcal	**pop(E)**	Kcal	**pop(G)**	4,3,34,49	3,34,49,55	34,49,55,54	48,54,55,49	35,48,54,55	32,35,48,54	31,32,35,48	54,55,58,61	55,54,59,60
1	0.00	*60.8%*	0.00	*57.9%*	-163	151	-67	-79	64	83	177	80	-31
22	0.82	*15.2%*	0.64	*19.8%*	178	-95	71	-170	-174	-69	-21	41	160
24	0.90	*13.2%*	1.43	*5.1%*	-173	75	51	-172	50	75	-173	-93	43
16	1.20	*8.0%*	1.15	*8.3%*	-173	75	50	-173	53	74	-173	46	-168
9	1.86	*2.6%*	1.13	*8.5%*	-3	-166	87	-54	87	-167	-4	-158	-46
11	3.30	*0.2%*	3.05	*0.3%*	-170	71	45	-173	149	-77	-8	46	-179

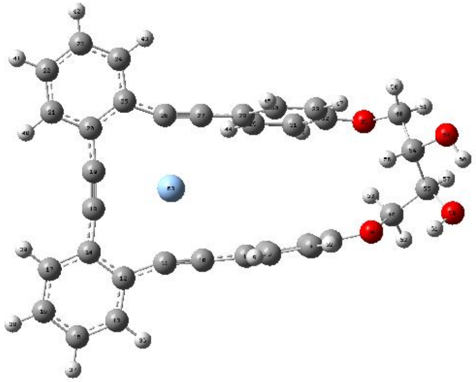
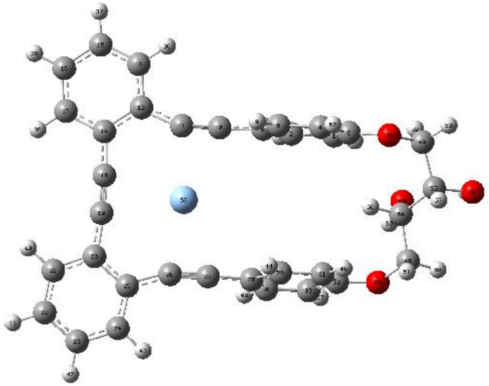
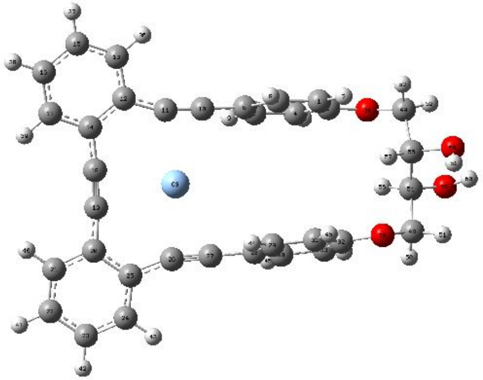
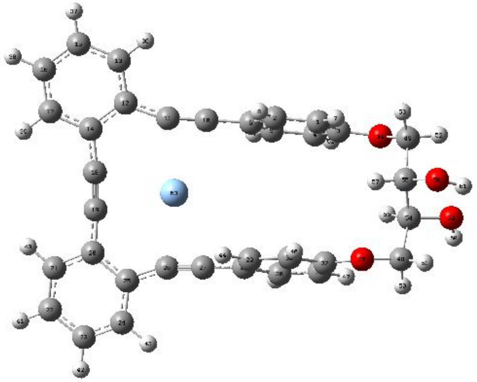
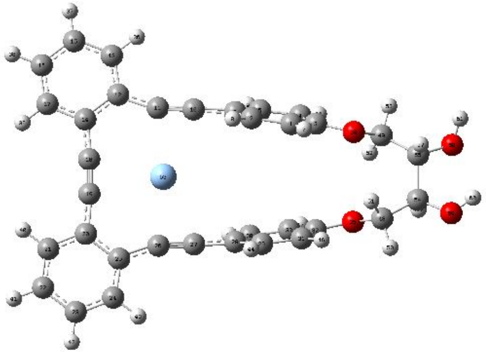
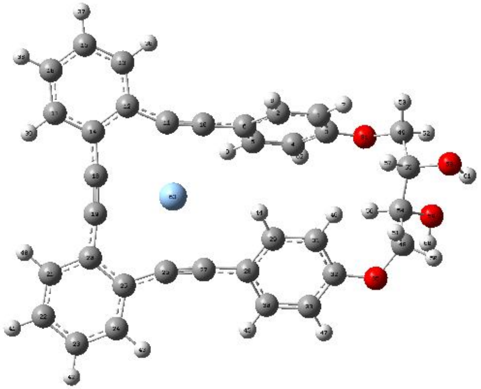

Calculated CD and Absorption spectra for *p,p*-**1** in CH_2Cl_2 (left) and in presence of Ag (right). The black trace is the average spectrum over calculated conformers. (average on energy). The spectra are calculated at about 30 nm higher wavelength than observed. In presence of Ag, the CD spectrum is very low (as observed).
All calculations have been done for configuration (1S,2S). Calculations suggest that the *P* conformation is favored .

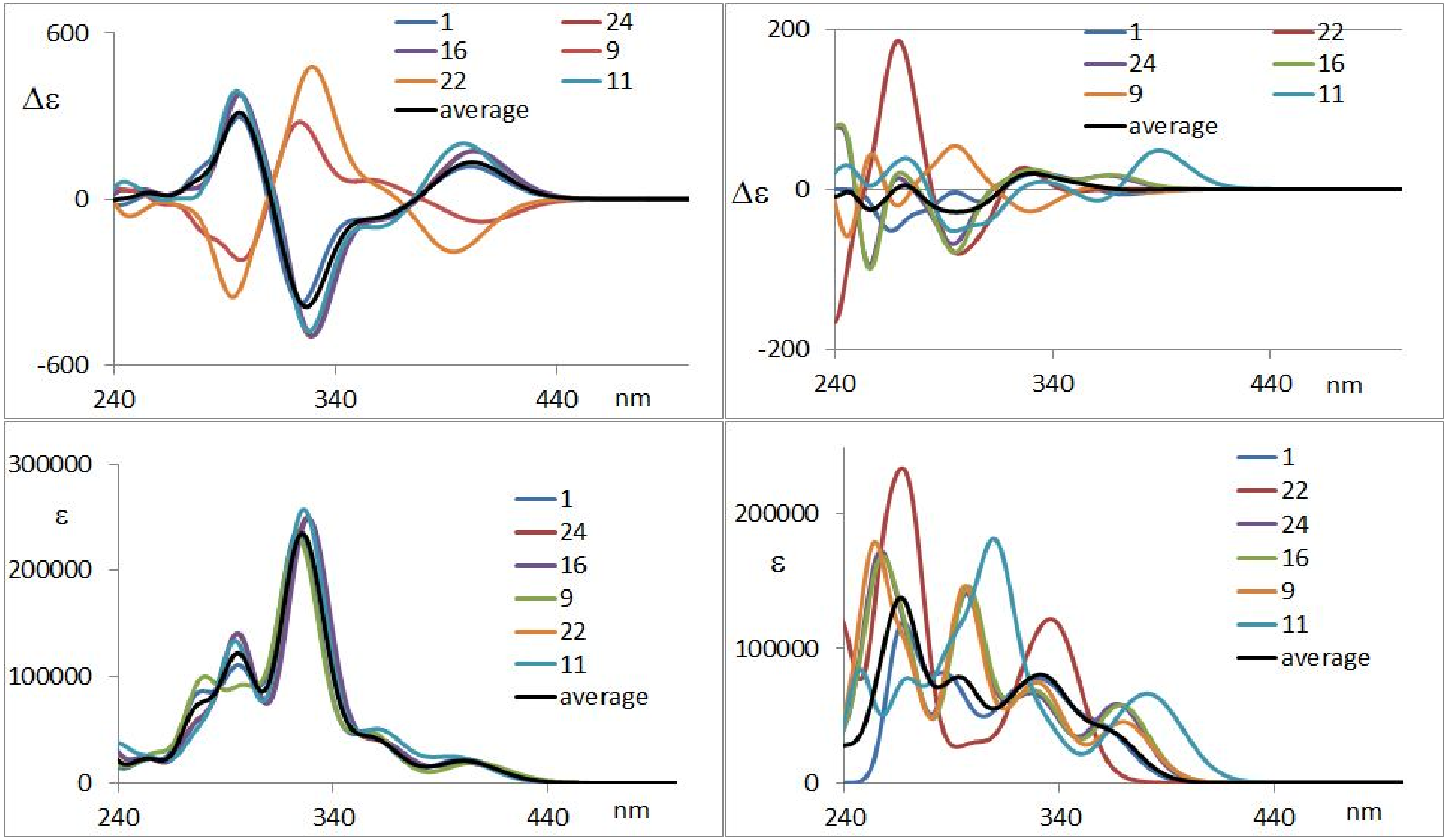

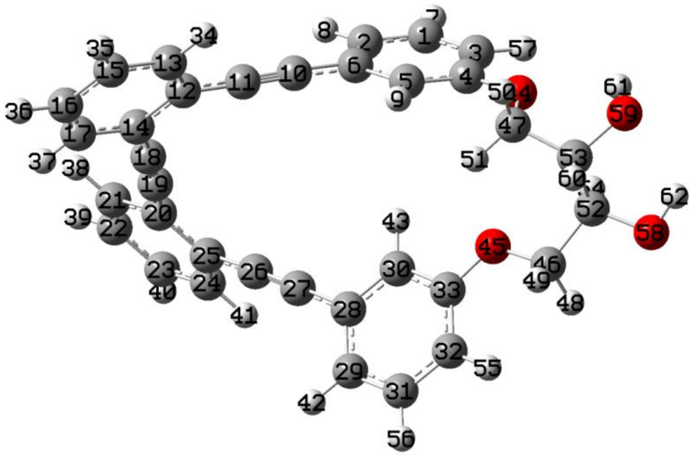

Conformational study of compound *m,m*-2 (*1S,2S*)

From all geometries found by MM, structures within 5 Kcal/mol have been optimized at b3lyp/6-31g* level in vacuo

conf	Kcal	pop (E)	5,4,44,47	4,44,47,53	44,47,53,52	46,52,53,47	45,46,52,53	33,45,46,52	30,33,45,46	52,53,59,61	53,52,58,62	helicity
**2**	**0.00**	***36.5%***	**8**	**160**	**-65**	**-64**	**76**	**175**	**-176**	**81**	**-40**	**P**
**1**	**0.18**	***27.1%***	**-176**	**77**	**74**	**-67**	**-63**	**165**	**-158**	**-39**	**80**	**M**
**17**	**0.65**	***12.2%***	**-9**	**-76**	**-178**	**-166**	**63**	**-153**	**155**	**167**	**39**	**M**
**4**	**0.97**	***7.1%***	**-175**	**173**	**77**	**-62**	**-61**	**155**	**-141**	**-41**	**79**	**M**
**31**	**1.06**	***6.1%***	**-3**	**76**	**61**	**-70**	**-59**	**172**	**-160**	**-38**	**79**	**P**
**3**	**1.37**	***3.6%***	**158**	**-164**	**67**	**-72**	**-174**	**-178**	**-5**	**-41**	**172**	**M**
**35**	**1.78**	***1.8%***	**-172**	**174**	**-81**	**66**	**-81**	**174**	**-172**	**96**	**96**	**M**
**9**	**2.17**	***0.9%***	**-173**	**77**	**53**	**-81**	**-173**	**172**	**-2**	**-38**	**170**	**M**
13	2.37	*0.7%*	180	-83	-32	-71	177	-90	-6	-35	-176	M
26	2.54	*0.5%*	114	-82	-173	-172	66	-92	150	160	42	P
11	2.56	*0.5%*	-152	71	73	-61	-170	-172	5	-45	172	P
39	2.57	*0.5%*	-158	69	53	-169	-174	-80	90	41	157	P
29	2.64	*0.4%*	15	-99	-169	-80	60	-171	161	167	-39	P
32	2.70	*0.4%*	-155	167	-72	70	180	177	8	90	173	P
6	2.82	*0.3%*	62	-163	-173	-65	74	-159	163	174	-43	P
7	2.89	*0.3%*	5	149	-48	-52	-60	163	1	-43	76	P

10	3.10	*0.2%*	170	-170	85	-64	82	-173	170	-161	-45	P
37	3.33	*0.1%*	-161	160	-77	61	179	166	-64	89	173	M
28	3.41	*0.1%*	-176	177	75	-65	-55	158	8	-154	-44	P
23	3.48	*0.1%*	170	-172	84	-62	80	-172	170	-38	67	P
5	3.64	*0.1%*	-61	-91	-180	-62	-42	169	-167	178	-42	M
8	3.64	*0.1%*	-151	157	-38	-54	-173	-155	-4	-46	180	M
18	3.90	*0.1%*	-177	-81	-20	-65	178	-88	110	-40	178	P
22	3.94	*0.0%*	179	81	70	-67	-50	172	-168	-153	-44	M
16	3.96	*0.0%*	18	-86	-169	-52	-37	167	-159	174	-47	P
40	3.97	*0.0%*	158	-88	76	-169	60	74	9	-168	45	P
38	3.98	*0.0%*	-152	157	-50	-65	75	173	-174	-44	-154	M
33	4.06	*0.0%*	-168	172	-50	-66	68	81	178	-33	68	M
25	4.08	*0.0%*	2	-176	-173	-67	109	-88	121	174	-43	P
14	4.18	*0.0%*	90	-93	180	-64	-41	173	-168	178	-41	P
12	4.27	*0.0%*	-54	-99	-171	-71	74	-141	28	169	-42	M
15	4.46	*0.0%*	-74	-71	172	-66	-59	164	-157	-40	77	M
24	5.03	*0.0%*	0	162	-59	-41	-59	162	0	71	71	P
20	5.13	*0.0%*	81	-166	-175	-64	-52	108	33	178	-41	P
21	5.27	*0.0%*	3	157	-52	-51	-50	155	3	-163	-49	P
19	5.50	*0.0%*	-115	133	-56	-53	-50	169	-5	75	-42	M
36	7.38	*0.0%*	-134	137	-43	-54	-54	171	-5	-47	-163	M

Eight conformers with not negligible populations have been optimized considering implicit solvent (iefpcm).

	Kcal	pop (E)	Kcal	pop(G)	5,4,44,47	4,44,47,53	44,47,53,52	46,52,53,47	45,46,52,53	33,45,46,52	30,33,45,46	52,53,59,61	53,52,58,62	
2	0.00	*43.5%*	0.00	*50.4%*	7	161	-64	-63	78	178	-178	78	-40	P
1	0.27	*27.8%*	1.07	*8.3%*	-177	77	75	-66	-61	166	-161	-39	78	M
17	0.88	*9.9%*	0.82	*12.7%*	-12	-75	-179	-165	64	-152	155	165	39	M
4	0.97	*8.4%*	0.50	*21.7%*	-176	174	80	-61	-59	155	-147	-41	76	M
31	1.23	*5.4%*	2.30	*1.0%*	-4	77	62	-71	-57	172	-161	-38	77	P
35	1.61	*2.9%*	2.23	*1.2%*	-172	174	-81	65	-81	174	-172	94	94	M
3	1.92	*1.7%*	1.52	*3.9%*	157	-164	69	-72	-176	-178	-5	-39	180	M
9	2.88	*0.3%*	2.34	*1.0%*	-173	78	54	-81	-176	172	-2	-36	177	M

The attribution of an helicity *P/M* in the table, does not take into account that structures can be highly distorted, helicity correlates with the sign of the first transition however important shifts in wavelength are obtained, and can be important in the average spectrum

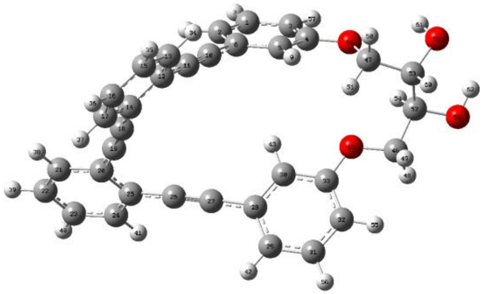
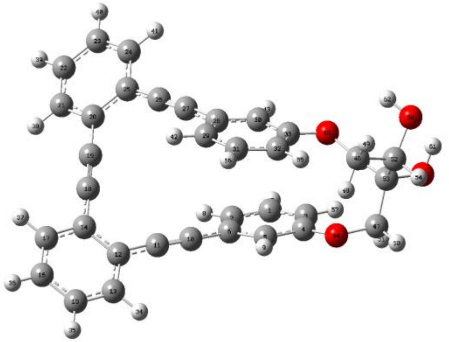
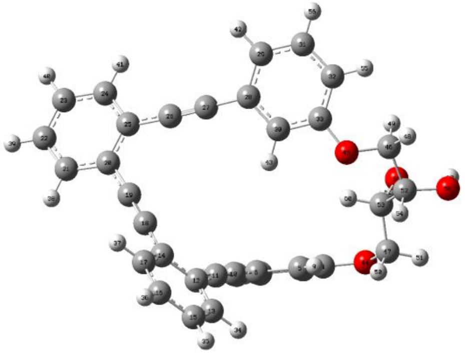
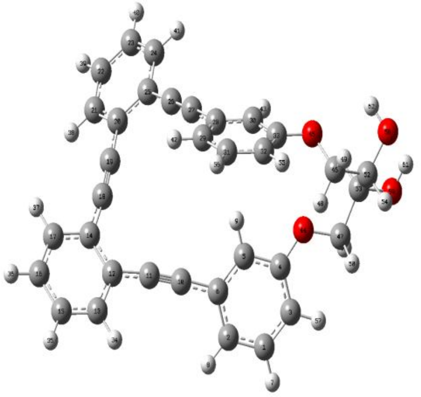

The structures thus obtained have been optimized with an Ag atom (iefpcm, b3lyp/6-31g*)

	Kcal	pop (E)	Kcal	pop(G)	5,4,44,47	4,44,47,53	44,47,53,52	46,52,53,47	45,46,52,53	33,45,46,52	30,33,45,46	52,53,59,61	53,52,58,62
2	0.00	*62.1%*	0.00	*60.6%*	6	162	-63	-67	75	-175	173	78	-38
31	0.85	*14.7%*	0.92	*12.7%*	-13	86	60	-77	-60	165	-160	-33	78
4	1.08	*10.1%*	1.52	*4.7%*	-162	165	82	-57	-58	162	-155	-42	74
1	1.30	*6.9%*	1.46	*5.1%*	-177	76	88	-61	-60	175	-166	-41	77
3	1.74	*3.3%*	1.47	*5.0%*	165	-157	75	-70	175	-167	-12	-38	-170
17	1.95	*2.3%*	1.03	*10.7%*	-15	-75	-173	-166	67	-136	156	159	38
9	2.89	*0.5%*	2.45	*1.0%*	-172	76	58	-77	-176	176	-2	-37	-178
35	3.50	*0.2%*	3.37	*0.2%*	-176	-178	-79	80	-79	-178	-176	92	92

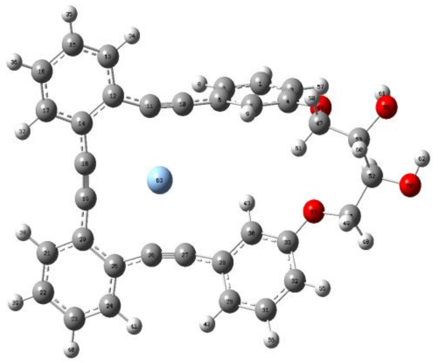

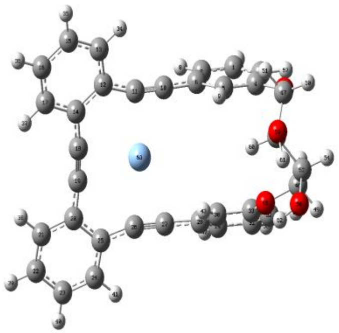

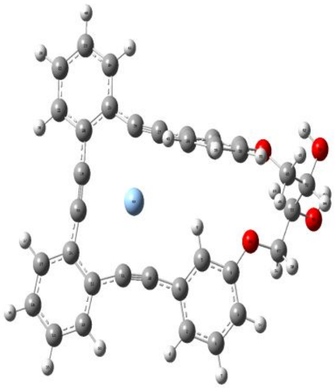

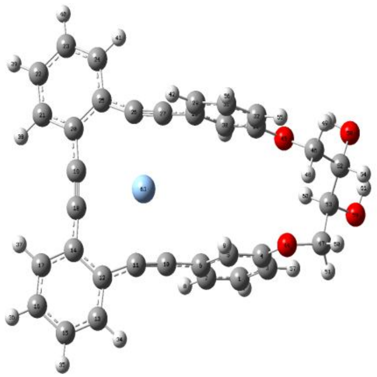

Calculated CD and Absorption spectra for *m,m*-**2** in CH_2Cl_2 (left) and in presence of Ag (right). The black trace is the average spectrum over calculated conformers. (average on energy). The spectra are calculated at about 30 nm higher than observed. All calculations have been done for configuration (*1S,2S*), the experimental spectra presented in the text concern the (*1R,2R*) enantiomer, as established by X-ray data. The average CD spectrum is weak due to the presence of both M and P conformers. The average CD spectrum obtained in absence of Ag has been superimposed in red on the spectra obtained in presence of Ag (right.top panel) for sake of comparison. Despite the approximated method used, we obtain an acceptable representation of the studied system: in particular notice the inversion of sign of CD spectrum in presence of Ag (top-right: compare the black trace for the Ag case with the red trace without Ag). The lowest energy calculated band is negative for (*1S,2S*) suggesting *M* helicity (in the table P and M classification does not take into account distortions: the two contributions seem to have similar populations, but the signal is dictated by *M* helicity).

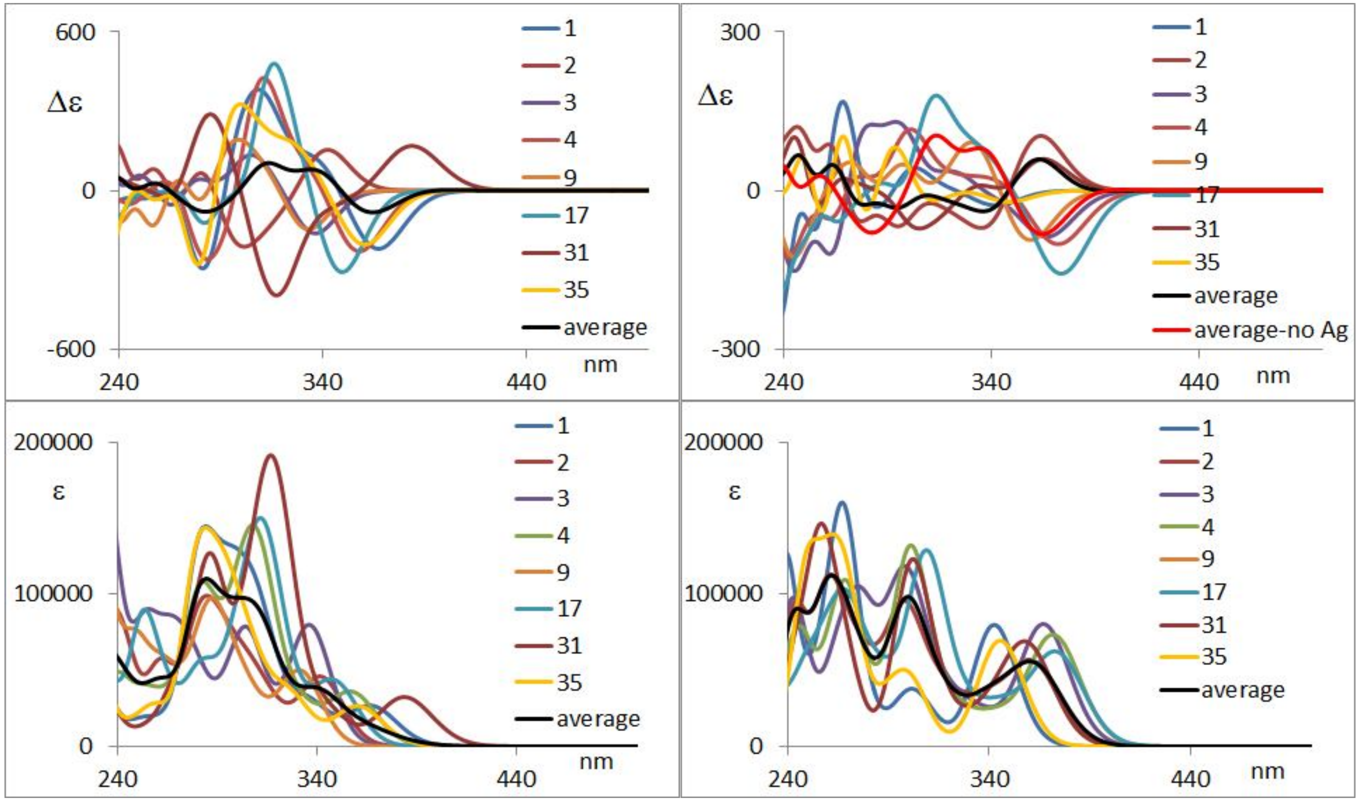

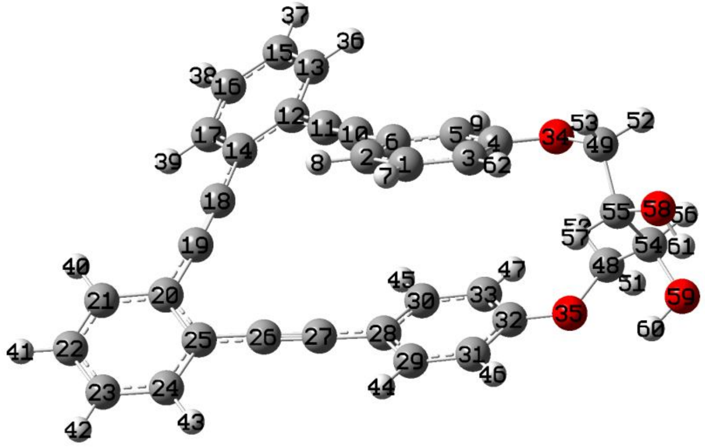

Conformational study of compound *p,m*-3 (*1S,2S*)

From all geometries found by MM, structures within 5 Kcal/mol have been optimized at b3lyp/6-31g* level in vacuo

	Kcal/mol	pop(E)	5,4,34,49	4,34,49,55	34,49,55,54	48,54,55,49	35,48,54,55	32,35,48,54	31,32,35,48	54,55,58,61	55,54,59,60	
**1**	**0.00**	**42.4%**	**7**	**155**	**-70**	**-82**	**57**	**77**	**3**	**84**	**-30**	**P**
**2**	**0.45**	**19.9%**	**-179**	**81**	**64**	**-78**	**-71**	**144**	**-160**	**-32**	**83**	**M**
**3**	**0.91**	**9.1%**	**-151**	**153**	**-65**	**-78**	**58**	**81**	**176**	**81**	**-33**	**M**
**4**	**0.98**	**8.1%**	**-9**	**-77**	**-168**	**-170**	**72**	**-86**	**-23**	**160**	**41**	**M**
**5**	**1.12**	**6.4%**	**-16**	**89**	**60**	**-80**	**-70**	**128**	**39**	**-31**	**83**	**P**
**6**	**1.42**	**3.8%**	**145**	**-90**	**74**	**-166**	**-171**	**-70**	**163**	**39**	**162**	**P**
**7**	**1.46**	**3.6%**	**174**	**179**	**72**	**-70**	**-66**	**132**	**-146**	**-37**	**81**	**M**
**8**	**1.62**	**2.8%**	**-7**	**-171**	**-176**	**-64**	**82**	**-126**	**-24**	**176**	**-43**	**M**
**9**	**2.14**	**1.1%**	**8**	**-101**	**73**	**-169**	**-170**	**-62**	**-48**	**42**	**161**	**M**
**10**	**2.50**	**0.6%**	**10**	**175**	**-163**	**-71**	**55**	**66**	**14**	**165**	**-44**	**P**
**11**	**2.78**	**0.4%**	**-166**	**-85**	**-19**	**-64**	**-178**	**-75**	**166**	**-41**	**-180**	**P**
12	2.89	0.3%	165	-158	63	-87	175	-94	123	-32	175	P
13	2.90	0.3%	-154	160	80	-57	-61	154	24	-43	78	P
14	3.15	0.2%	17	-96	-171	-72	78	-120	162	171	-41	P

15	3.39	0.1%	170	-166	61	-92	174	-91	9	-29	178	P
16	3.47	0.1%	-7	-158	-176	-59	-50	113	-152	180	-43	M
17	3.55	0.1%	178	-175	83	-54	88	-160	172	-48	-162	P
18	3.60	0.1%	176	-172	85	-54	86	-162	174	-161	-49	P
19	3.72	0.1%	9	153	-73	-77	151	-80	-41	83	-34	P
20	3.95	0.1%	177	-174	83	-53	85	-160	173	-41	65	P
21	4.19	0.0%	86	-108	-172	-83	65	-110	-175	169	-37	P
22	4.20	0.0%	102	-95	175	-172	39	59	-157	170	44	P
23	4.32	0.0%	-152	155	-66	-73	148	-87	-56	80	-37	M
24	4.36	0.0%	2	160	-56	-88	50	79	3	-48	-96	P
25	4.58	0.0%	15	-117	60	-85	-178	-98	-73	-36	170	M
26	4.60	0.0%	-171	171	-48	-76	53	84	174	-41	-144	M
27	4.66	0.0%	116	-86	159	-76	-70	136	-146	-34	81	P
28	4.66	0.0%	106	-118	-178	-68	-53	117	32	180	-39	P
29	4.75	0.0%	180	83	56	-78	-55	159	-171	-143	-39	M
30	5.03	0.0%	9	166	-51	-46	-39	143	30	74	-45	P
31	5.04	0.0%	-160	150	-52	-72	169	-105	-71	-36	-172	M
32	5.06	0.0%	-13	-144	173	-57	-60	116	-148	-45	76	M
33	5.17	0.0%	27	113	-60	-73	179	-103	-52	-37	180	P
34	5.34	0.0%	8	163	-43	-45	-47	145	33	-45	72	P
35	5.83	0.0%	-69	174	-60	-55	-44	119	-148	78	-42	M
36	6.09	0.0%	-6	-162	-175	-44	-63	119	-148	175	72	M
37	6.72	0.0%	-139	159	-31	-45	-45	148	-154	-45	72	M
38	6.81	0.0%	8	163	-44	-46	-39	145	28	-163	-51	P
39	6.95	0.0%	8	163	-42	-45	-41	145	-150	-51	-162	P
40	7.48	0.0%	7	168	-59	-25	-56	151	29	76	73	P

Eleven conformers with not negligible populations have been optimized considering implicit solvent (ief-pcm).

	Kcal/mol	pop(E)	Kcal/mol	pop(G)	5,4,34,49	4,34,49,55	34,49,55,54	48,54,55,49	35,48,54,55	32,35,48,54	31,32,35,48	54,55,58,61	55,54,59,60	
1	0.00	39.0%	0.00	11.9%	5	156	-69	-83	57	77	3	82	-30	P
2	0.34	22.1%	-0.30	19.7%	-180	82	65	-78	-69	145	-161	-33	80	M
3	0.58	14.5%	0.10	10.1%	-157	156	-63	-76	61	80	179	79	-34	M
4	1.04	6.7%	-0.23	17.4%	-11	-76	-168	-170	73	-87	-22	159	40	M
5	1.11	6.0%	1.00	2.2%	-16	90	61	-81	-70	127	39	-31	81	P
7	1.16	5.5%	-0.18	16.0%	172	-180	74	-70	-65	133	-148	-37	78	M
6	1.50	3.1%	0.42	5.8%	145	-91	75	-166	-172	-69	161	38	160	P
8	1.83	1.8%	-0.14	15.0%	-8	-170	-178	-64	84	-126	-23	-176	-41	M
9	2.22	0.9%	1.20	1.6%	8	-101	74	-170	-171	-62	-42	41	159	M
11	3.09	0.2%	2.28	0.3%	-165	-86	-21	-64	180	-74	164	-39	-171	P
10	3.20	0.2%	2.51	0.2%	11	173	-163	-70	55	66	14	169	-44	P

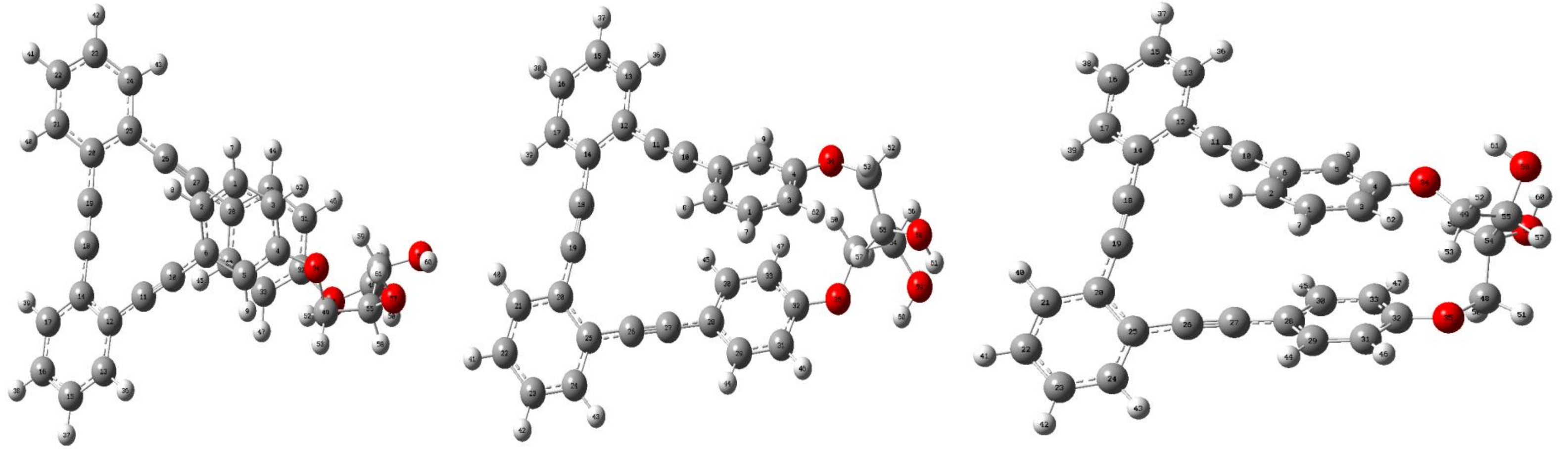

The structures thus obtained have been optimized with an Ag atom (iefpcm, b3lyp/6-31g*)

	Kcal/mol	pop(E)	Kcal/mol	pop(G)	5,4,34,49	4,34,49,55	34,49,55,54	48,54,55,49	35,48,54,55	32,35,48,54	31,32,35,48	54,55,58,61	55,54,59,60
3	0.00	76.8%	0.00	69.0%	-171	176	-59	-69	69	74	-174	77	-37
2	1.07	12.6%	0.68	21.9%	172	86	77	-70	-66	153	-165	-37	78
7	1.24	9.4%	1.35	7.1%	-177	171	77	-67	-62	141	-154	-38	76
6	2.93	0.5%	2.86	0.6%	171	-96	64	-169	176	-71	159	42	169
1	3.16	0.4%	2.67	0.8%	-3	167	-70	-94	48	79	2	84	-18
11	3.58	0.2%	3.58	0.2%	-165	-85	-25	-68	172	-78	154	-36	-167
4	4.27	0.1%	3.90	0.1%	-3	-82	-160	-172	76	-89	-11	152	42
8	4.37	0.0%	3.02	0.4%	-6	-169	-177	-63	87	-125	-15	-176	-42
5	4.57	0.0%	4.24	0.1%	-12	98	63	-86	-77	102	33	-26	87
10	5.55	0.0%	4.66	0.0%	13	166	-164	-75	50	67	8	172	-42
9	6.06	0.0%	5.27	0.0%	13	-110	74	-168	-164	-61	-27	41	148

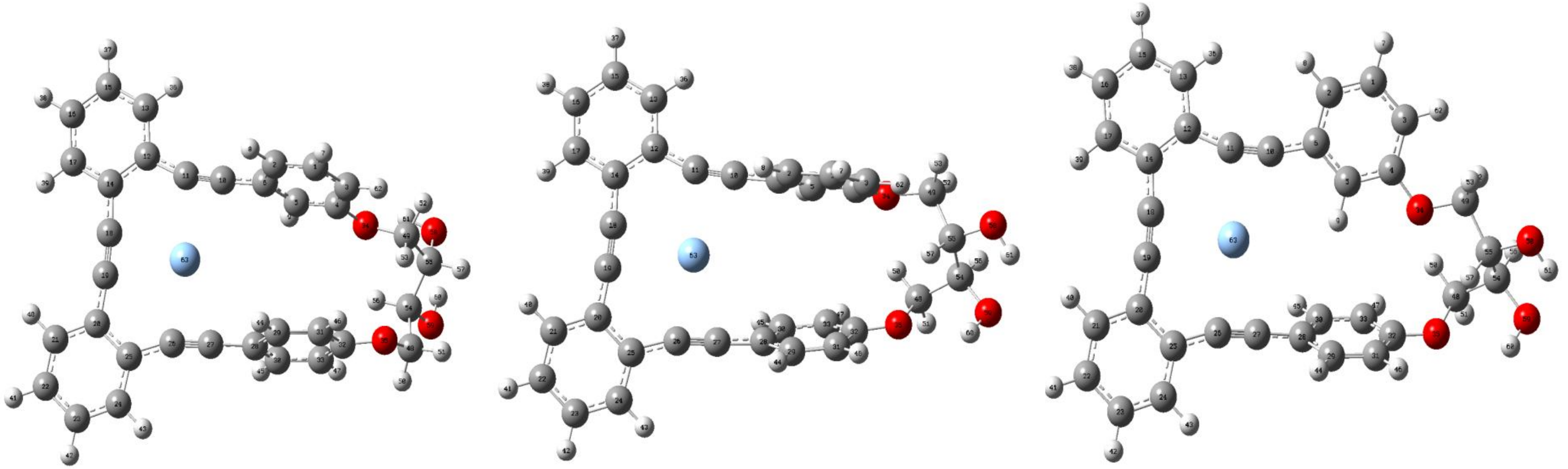

Calculated CD and Absorption spectra for *p,m*-**3** in CH_2Cl_2 (left) and in presence of Ag (right). The black trace is the average spectrum over calculated conformers. (average on energy). The spectra are calculated at about 30 nm higher than observed. All calculations have been done for configuration 1S,2S. The

average CD spectrum is very low due to the presence of both M and P conformer. The average CD spectrum obtained in absence of Ag has been superimposed in red on the spectra obtained in presence of Ag (right.top panel) for sake of comparison. Obviously, populations of the different conformers cannot be certain from a DFT calculations, however an inversion of sign of CD spectrum in presence of Ag is obtained.

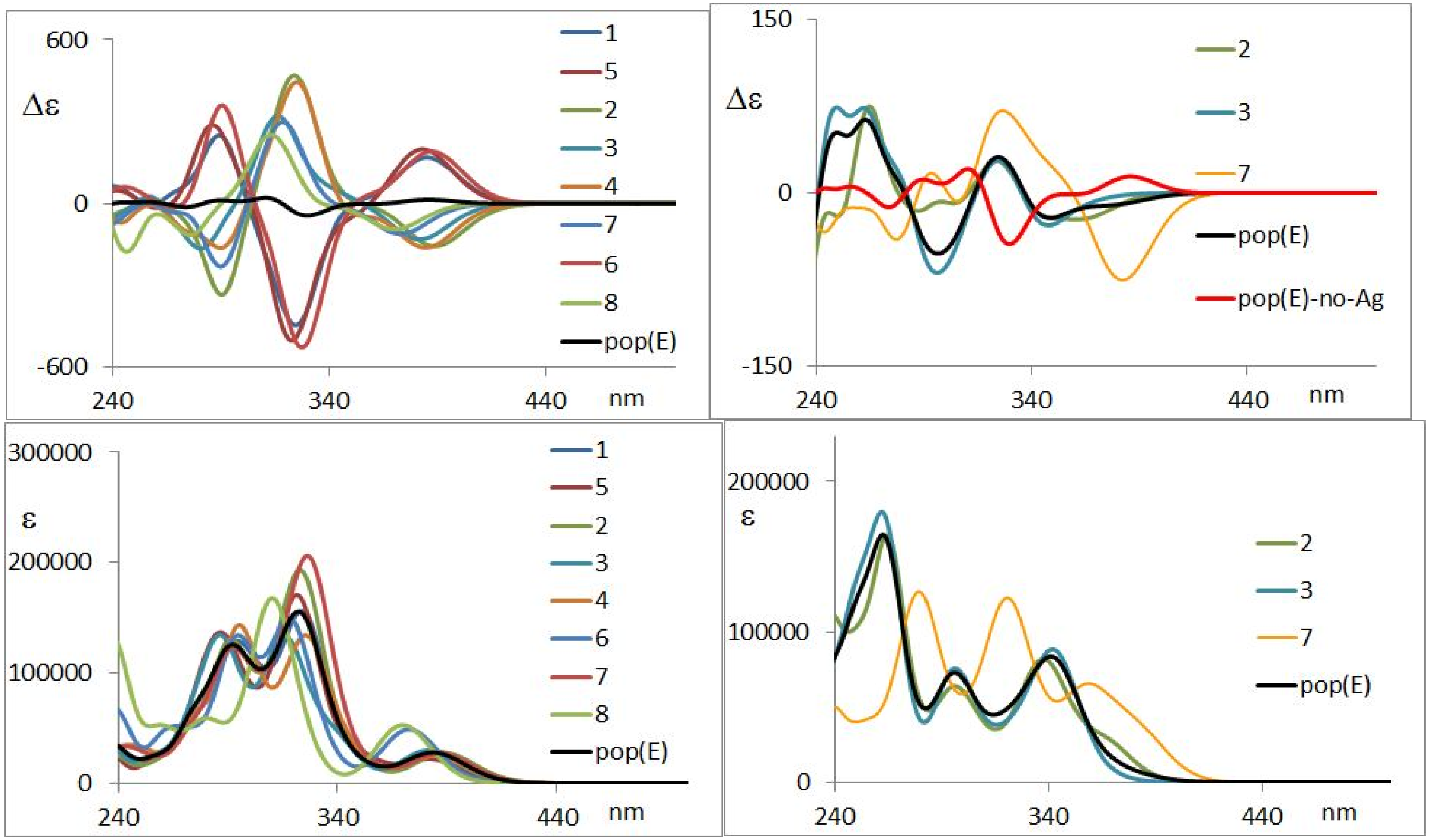

REFERENCES:

1. E. G. Rochow, *Inorg. Synth.*1960, **6**, 218.

2. N. Fuentes, A. Martín-Lasanta, L. Álvarez de Cienfuegos, R. Robles, D. Choquesillo-Lazarte, J. M. García-Ruiz, A. J. Mota, L. Martínez-Fernández, I. Corral, D. J. Cárdenas, M. Ribagorda, M. C. Carreño and J. M. Cuerva, *Angew. Chem. Int. Ed.*, 2012, ***51***, 13036-13040.

3.Bruker, APEX2 Software, V2013.2, Bruker AXS Inc., Madison, Wisconsin, USA, 2013.

4.G. M. Sheldrick, *ActaCrystallogr., Sect. A: Found. Crystallogr.*, 2008, ***64***, 112–122.

5. E. Castiglioni, S. Abbate and G. Longhi, *Appl. Spectrosc.*, 2010, ***64***, 1416–1419

6. B. Valeur, M. N. Berberan-Santos, *Molecular Fluorescence. Principles and Applications*, 2nd ed., Wiley-VCH: Weinheim (Germany), 2012

7.J. Lakowicz, *Principles of Fluorescence Spectroscopy*, 3rd ed., Springer-Verlag: New York, 2006

8.. J. Olmsted,*J. Phys. Chem. 1979*, **83**, 2581–2584

9. T. Gasa, J. Spruell, W. Dichtel, T. Srensen, D. Philp, J. Stoddart, P. Kuzmič,*Chem. Eur. J.*, 2009, **15**, 106–116

10. M. J. Frisch, G. W. Trucks, H. B. Schlegel, G. E. Scuseria,M. A. Robb, J. R. Cheeseman, G. Scalmani, V. Barone, B. Mennucci, G. A. Petersson, H. Nakatsuji, M. Caricato,X. Li, H. P. Hratchian, A. F. Izmaylov, J. Bloino, G. Zheng,J. L. Sonnenberg, M. Hada, M. Ehara, K. Toyota, R. Fukuda,J. Hasegawa, M. Ishida, T. Nakajima, Y. Honda, O. Kitao,H. Nakai, T. Vreven, J. A. Montgomery, Jr., J. E. Peralta,F. Ogliaro, M. Bearpark, J. J. Heyd, E. Brothers, K. N. Kudin,V. N. Staroverov, R. Kobayashi, J. Normand, K. Raghavachari,A. Rendell, J. C. Burant, S. S. Iyengar, J. Tomasi, M. Cossi,N. Rega, J. M. Millam, M. Klene, J. E. Knox, J. B. Cross,V. Bakken, C. Adamo, J. Jaramillo, R. Gomperts, R. E. Stratmann,O. Yazyev, A. J. Austin, R. Cammi, C. Pomelli, J. W.Ochterski, R. L. Martin, K. Morokuma, V. G. Zakrzewski, G. A.Voth, P. Salvador, J. J. Dannenberg, S. Dapprich, A. D. Daniels,. Farkas, J. B. Foresman, J. V. Ortiz, J. Cioslowski and D. J.Fox, Gaussian 09 Revision D.01, Gaussian Inc. Wallingford CT, 2009.
